# Pyrones Identified as LuxR Signal Molecules in *Photorhabdus* and Their Synthetic Analogues Can Alter Multicellular Phenotypic Behavior of *Bacillus atropheaus*

**DOI:** 10.1021/acsomega.1c05508

**Published:** 2021-11-22

**Authors:** Aobha Hickey, Leticia M. Pardo, F. Jerry Reen, Gerard P. McGlacken

**Affiliations:** ^†^School of Chemistry, Analytical and Biological Chemistry Research Facility, ^‡^School of Microbiology, ^§^Synthesis and Solid State Pharmaceutical Centre, University College Cork, Cork T12 YN60, Ireland

## Abstract

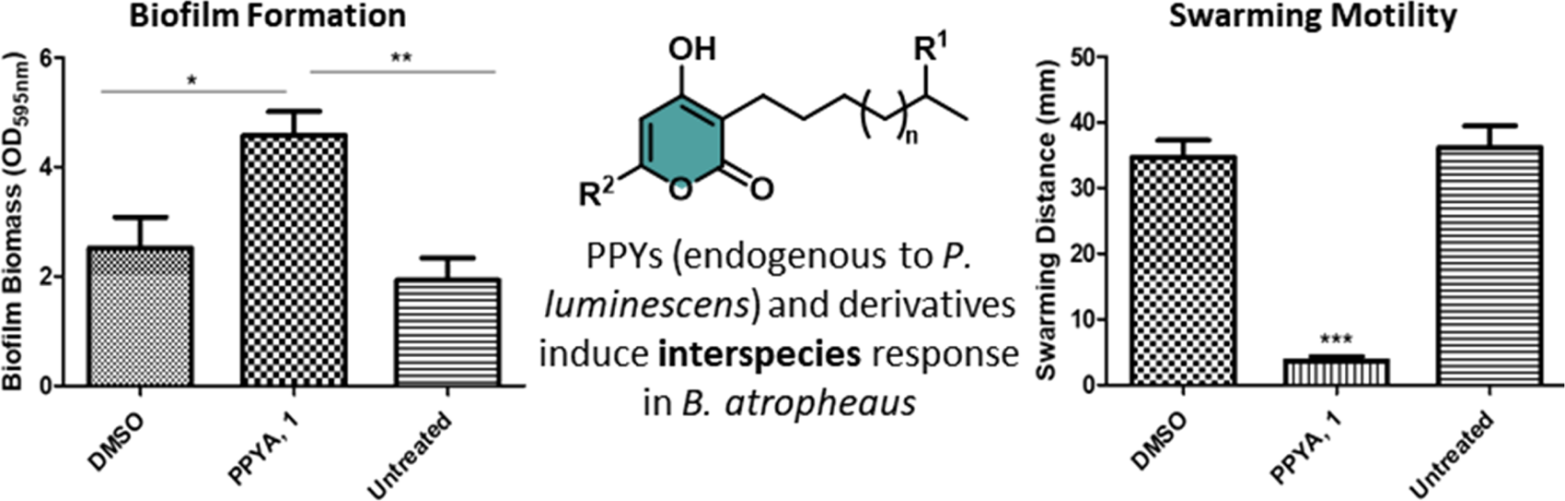

Individual bacteria communicate by the release and interpretation of small molecules, a phenomenon known as quorum sensing (QS). We hypothesized that QS compounds extruded by *Photorhabdus* could be interpreted by *Bacillus*—a form of interspecies communication. We interrogate the structure–activity relationship within the recently discovered pyrone QS network and reveal the exquisite structural features required for targeted phenotypic behavior. The interruption of QS is an exciting, nonbiocidal approach to tackling infection, and understanding its nuances can only be achieved by studies such as this.

## Introduction

Bacteria can coordinate their collective behavior through an elaborate communication network.^1^ This remarkable realization has offered new insights into the complexity of microbial infections.^1^ Cooperation of/within microbial consortia is governed by the extrusion and perception of small-molecule signals (called autoinducers—AIs), a phenomenon known as quorum sensing (QS). Thus, bacteria monitor their external environment and can significantly alter gene expression to act as a single multicellular organism if required.^2,3^ These interactions yield the capacity to accomplish tasks that are futile when performed by an individual bacterium^4^—typically bioluminescence, secondary metabolite synthesis, and perhaps, most importantly, biofilm formation and virulence factor production.^5−8^ Through this ability to coordinate behavior and form biofilms, QS allows bacteria to become more potent pathogens, less susceptible to antibiotic treatments, and often facilitates the evolution of antibiotic resistance.^9,10^

**Figure 1:**
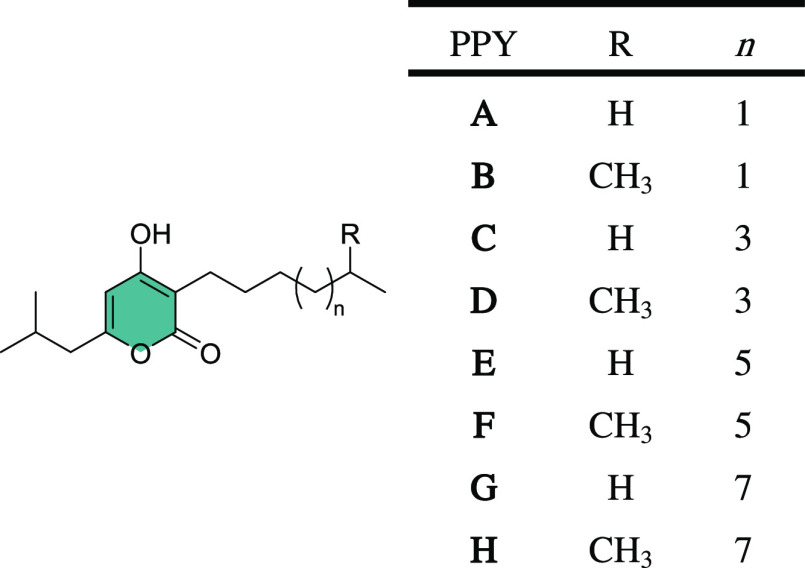
Photopyrones (PPYs) A–H isolated from Photorhabdus luminescens.

The prototypical QS model in Gram-negative bacteria is comprised of a LuxI-type AI synthase and a LuxR-type receptor, with *N*-acyl homoserine lactones (AHLs) being the most predominant class of AIs generated and received by these proteins.^11^ Although not fully understood, systems lacking any LuxI-like AHL synthase can still encompass proteins with homology to LuxR-type receptors, namely LuxR orphans or solos.^12,13^ Seminal reports by Heermann and co-workers explicitly target the concept that non-AHL producing bacteria can employ these receptors in the detection of other endogenously synthesized compounds and operate a QS signaling pathway.^14,15^ Critically, this group unearthed “photopyrones” (PPYs) participating in the QS network of the Gram-negative pathogen *Photorhabdus luminescens* through an orphan LuxR receptor. The quorum in this case activates a signaling cascade that transcribes the operon responsible for the *Photorhabdus* clumping factor (Pcf)—a biofilm-like phenotype that plays a vital role in the pathogenicity of these bacteria.^14,16,17^ These findings encompass the first demonstration of 2-pyrones, specifically 3-alkyl-4-hydroxy-6-isobutyl-2*H*-pyran-2-ones (Figure 1), acting as signaling molecules in any bacterial strain.^14^

Although the findings specified the detection of endogenously produced PPY signals, it is also known that orphan receptors can be used to respond to exogenous signals, produced by coinhabiting microorganisms, for example.^15,18,19^ Following on from our work describing the interspecies and interkingdom activity of the alkyl hydroxyquinolone signals 2-heptyl-4-quinolone (HHQ) and *Pseudomonas* quinolone signal (PQS) produced by *Pseudomonas aeruginosa*,^20−26^ this report describes interspecies behavioral control exerted by the *P. luminescens* pyrone signals on a model organism, *Bacillus atropheaus* subtilis var. niger (globigii) termed *B. atropheaus* hereafter. Importantly, this Gram-positive, aerobic bacterium utilizes QS in the formation of biofilms,^27^ making it a suitable model for analysis of signal-based interference with cell–cell communication. Entomopathogenic *Photorhabdus* and *Bacillus* species have demonstrated a close inter-relationship, working synergistically in some infections^28,29^ and exchanging toxin systems in others.^30^ The emerging interspecies and interkingdom role in cell–cell communication signals and the close relationship between *Photorhabdus* and *Bacillus* species led us to investigate the possibility that 2-pyrones, similar to those produced by the former, could exert behavioral control over the latter.

A bacterial biofilm is a polymeric matrix structure that can grow on living or inert surfaces.^31^ Its formation involves a complex multistage process, underpinned by a signaling-based communication system that spans from the initial attachment phase through to maturation and dispersion. This lifestyle is adopted by up to 80% of infections in humans,^32^ frequently allowing the bacterial colony to circumvent the host immune system and increase resistance to antibacterial agents.^10^ Swarming is also governed by a complex QS-based communication system, which directs multiple aspects of behavior, including head to tail connections between individual bacterial cells in this and other *Bacillus* species.^33^ Both of these essential phenotypes, which are linked to persistence, virulence, and antibiotic resistance in bacteria, were investigated using *B. atropheaus*.^34,35^

## Results and Discussion

**Figure 2:**
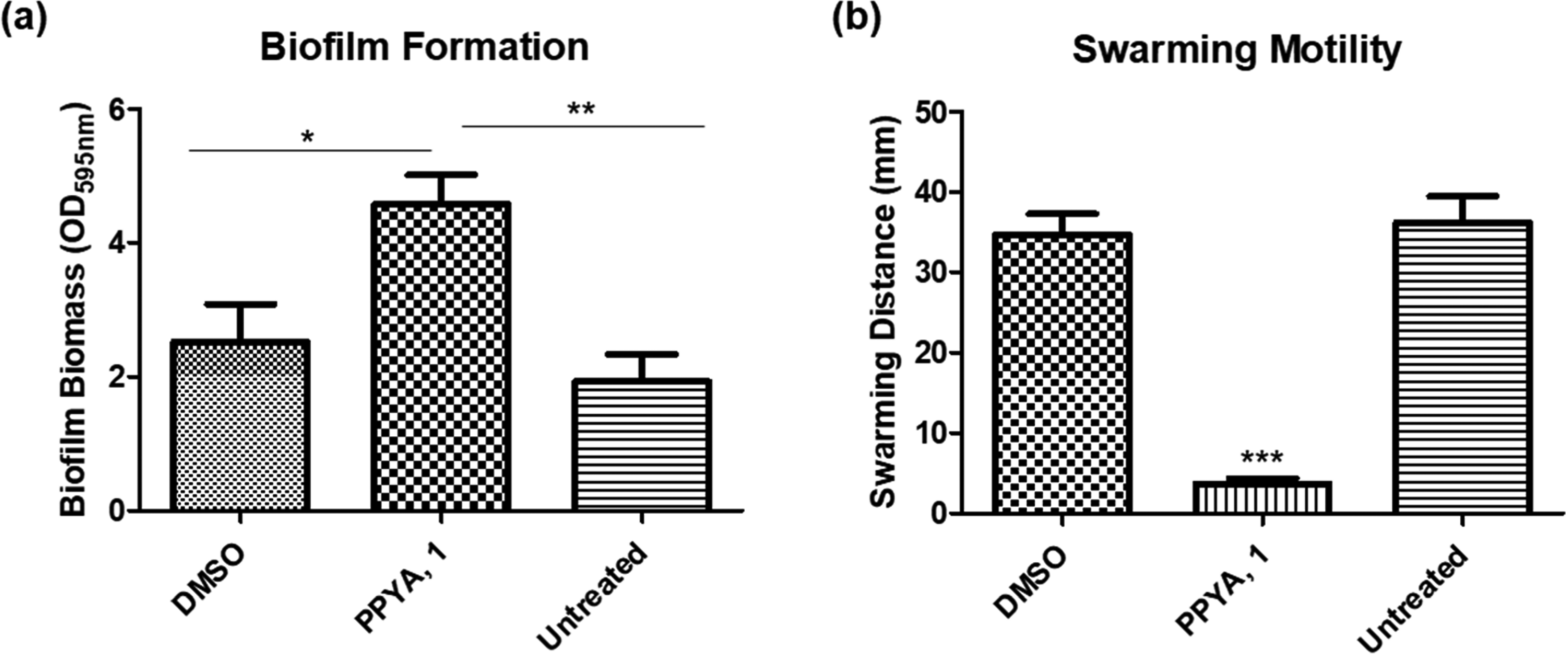
(a) Biofilm formation in *B. atropheaus* in the presence **PPYA** at 50 μM in DMSO presented as Abs_595nm_ following crystal violet staining. (b) Swarming motility of *B. atropheaus* with **PPYA** or carrier control. Data presented are the average (±standard error of the mean (SEM)) of three independent biological replicates. Statistical analysis was performed by one-way analysis of variance (ANOVA) with Bonferroni post hoc corrective testing (*p ≤ 0.05; **p ≤ 0.005; ***p ≤ 0.001).

The simplest of the natural signals, isolated from *Photorhabdus*, **PPYA**, (**1**) was initially synthesized and tested for biofilm and swarming motility altering activity.^36,37^ Addition of **PPYA 1** (50 μM) to media prior to inoculation with *B. atropheaus* cultures led to a dramatic increase in attached biofilm biomass when compared with dimethyl sulfoxide (DMSO) and untreated controls (Figure 2a). We reasoned that a low micromolar concentration would be in the physiological range and consistent with previous studies investigating the interspecies role of other quorum sensing molecules.^20,38,39^ In contrast, swarming motility was significantly repressed in the presence of **PPYA**, pyrone **1** (Figure 2b). This was independent of any growth-related effects, as determined by visual analysis of the biofilm formed, indicating a shift toward stronger pellicle formation at the liquid–air interface (SI, Figure S1). The addition of a reduced concentration of compound **1** (10 μM) did not elicit a response from *B. atropheaus* at the same cell seeding density, indicating dose-dependency to the effects observed (SI, Figure S1). The influence of **1** on biofilm formation and swarming motility in *B. atropheaus* has extensive potential implications. This suggests that **1** has the propensity to induce interspecies activity beyond its inherent role in the signal-producing *Photorhabdus* species.

**Scheme 1:**
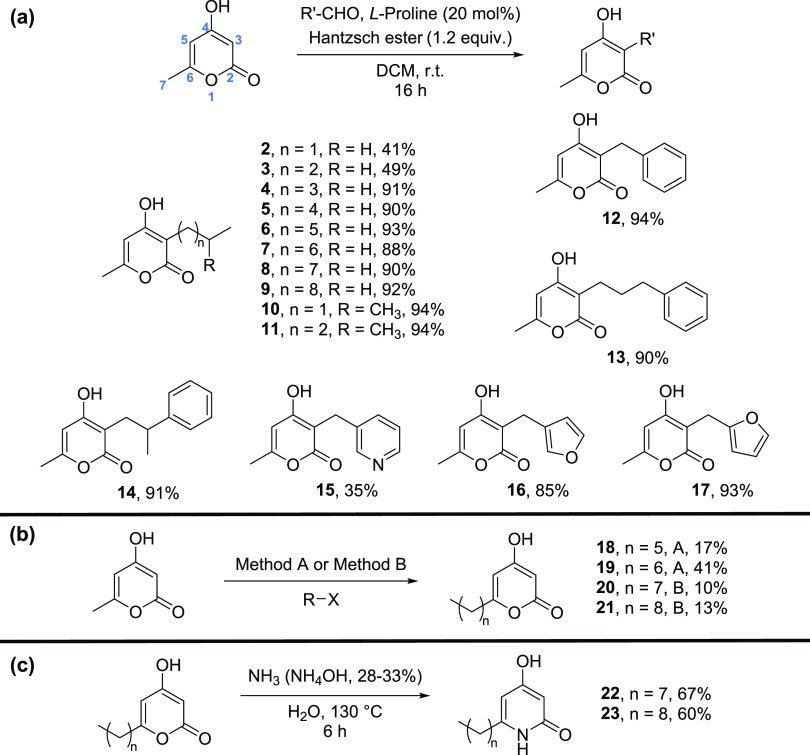
(a–c) 2-Pyrone and 2-Pyridinone Derivatives Synthesized The Hantzsch ester used here is 1,4-dihydro-2,6-dimethyl-3,5-pyridinedicarboxylate. Method A: (i) hexamethyldisilazane (HMDS) (3 mL/mmol), N_2_, 80 °C, 1 h and (ii) tetrahydrofuran (THF), *n*-BuLi (1.25 equiv), alkyl bromide (2.3 equiv), −78 °C–rt, 16 h. Method B: (i) *N*,*N*,*N*′,*N*′-tetramethylethylenediamine (TMEDA) (1.0 equiv), THF/hexamethylphosphoramide (HMPA) (5:1), *n*-BuLi (2.4 equiv), 0 °C, N_2_ and (ii) alkyl iodide (1.8 equiv), 0 °C–rt, 16 h (see the SI for details).

Following on from these promising results, we looked at preparing a suite of analogues (known and novel) to probe the structure–activity relationship. Diversification at C3 and C6 was targeted, along with some modification of the pyrone core (Scheme 1).^36,37^ We then investigated the impact of these analogues on biofilm and swarming properties in *B. atropheaus*.

**Figure 3:**
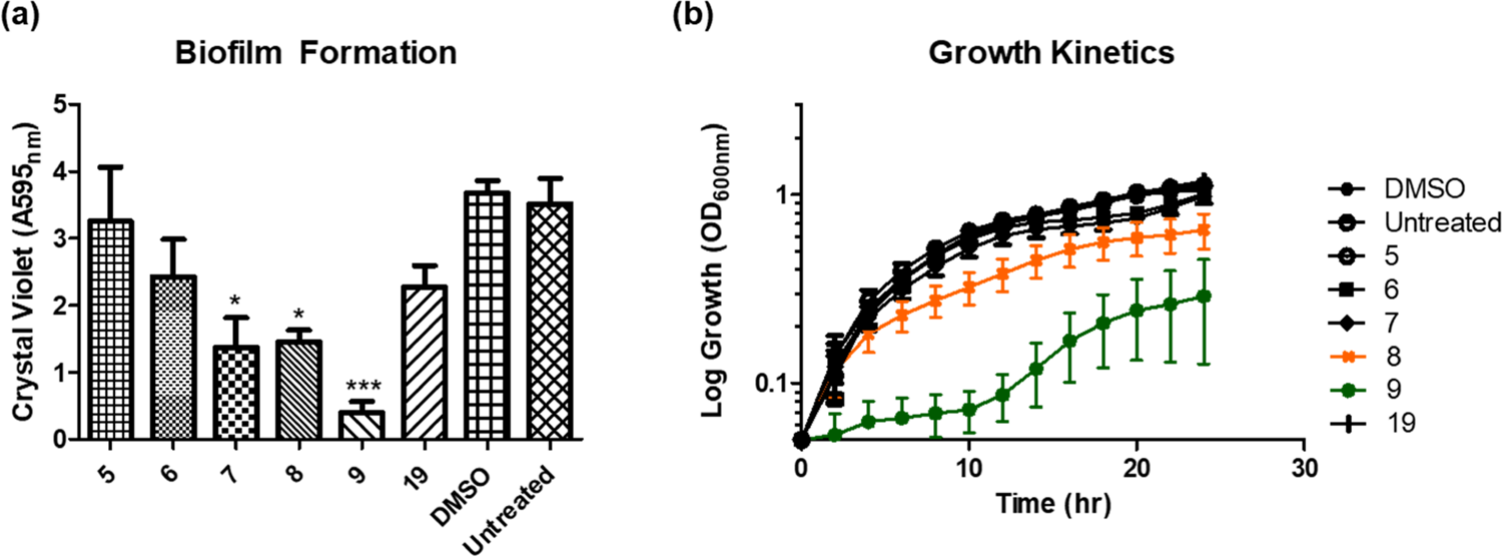
(a) Antibiofilm activity of pyrone derivative compounds at 50 μM in DMSO presented as Abs_595nm_ following crystal violet staining. Assays were performed in 24-well plates. (b) Growth curve analysis of *B. atropheaus* in the presence of pyrone derivatives (see the SI for details). All data presented are the average (±SEM) of at least three independent biological replicates. Statistical analysis was performed by one-way ANOVA with Bonferroni multiple comparison post hoc corrective testing (*p ≤ 0.05; ***p ≤ 0.001).

Based on the trend toward enhanced biofilm formation observed with the native pyrone signal **PPYA 1**, we examined the impact of our 2-pyrone derivatives on the ability of *B. atropheaus* to form biofilms and attach to the surface of multiwell plates. We started with analogues bearing a methyl group at C6 (**2**–**17**), which are relatively easy to synthesize. When tested at 50 μM, the following compounds exhibited antibiofilm activity: **7**, **8**, and **9**—in direct contrast to the activity of the natural pyrone signal **1** (SI, Figure S2). Although **6** led to a reduction in biofilm formation, this was not statistically significant when tested in 24-well plates (Figure 3a). It should be noted that all compounds exhibiting antibiofilm activity, contain a long alkyl chain at C3 (C_7_–C_10_). Subsequent validation analysis of lead derivatives through dose–response studies at 10, 30, and 50 μM confirmed the biofilm limiting activity of these compounds (SI, Figure S3).

To determine whether the influence on biofilm was simply a reflection of growth inhibition, *B. atropheaus* was grown in the presence of each compound and investigated temporally. In terms of growth kinetics profiling, while compounds **8** and **9** had a growth-limiting effect on *B. atropheaus*, growth was not comparably affected in the presence of pyrones **6** and **7** (Figure 3b). This clearly delineates the biofilm formation and growth, at least in compounds **6** and **7**. We, therefore, propose these compounds as lead compounds for antibiofilm activity, potentially working through interference with signaling mechanisms. None of the other compounds, with shorter or longer (linear, branched alkyl chains, aryl, heterocyclic) groups at C3 showed considerable antibiofilm activity. For the activity of all 17 analogues, see the SI.

**Figure 4:**
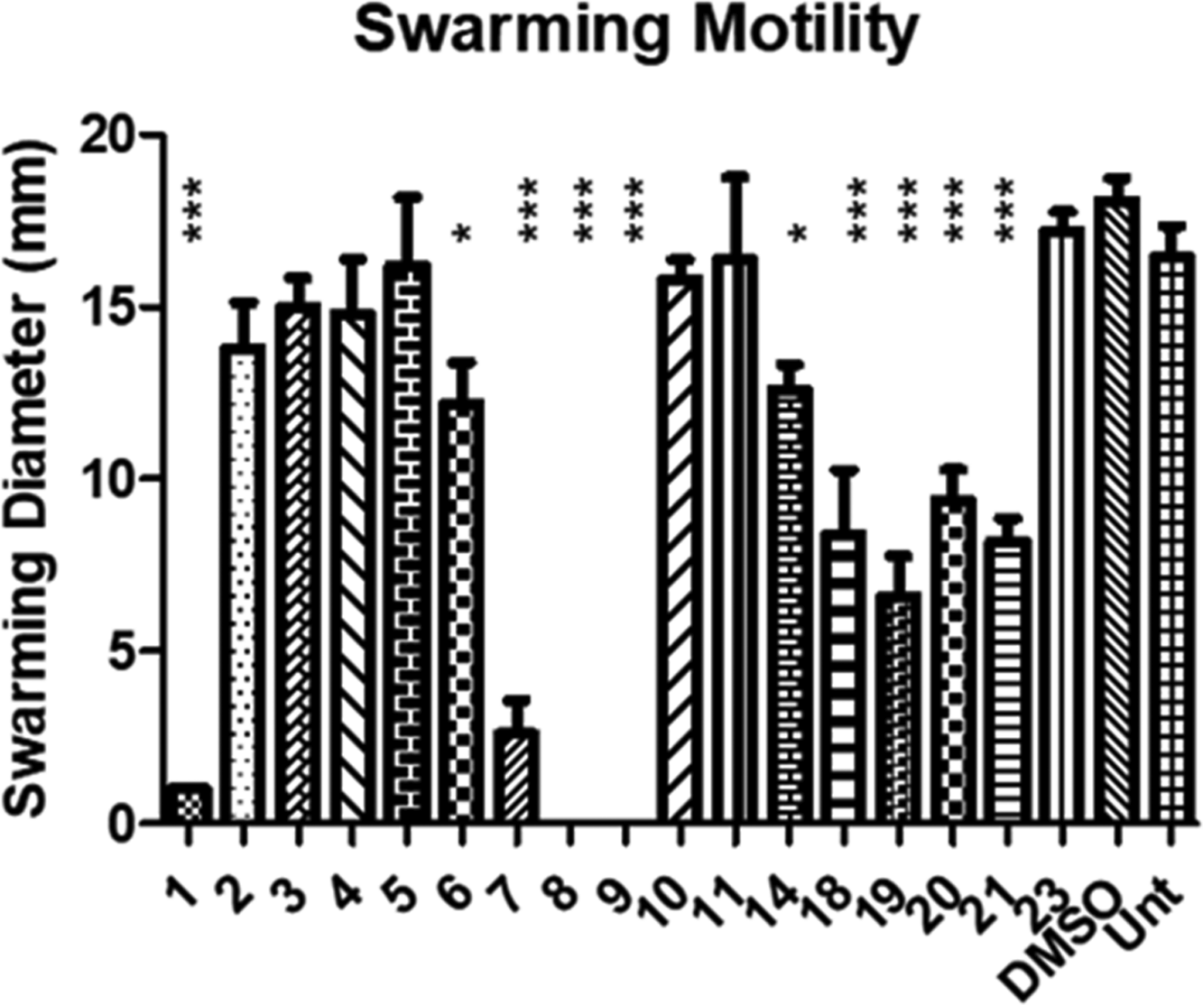
Swarming motility of B. atropheaus with 50 μM compound or carrier control (see the SI for details). All data presented are the average (±SEM) of at least three independent biological replicates. Statistical analysis was performed by one-way ANOVA with Bonferroni post hoc corrective testing (*p ≤ 0.05, ***p ≤ 0.001).

The ability of specific pyrone analogues to interfere with biofilm formation in *B. atropheaus* led us to examine the impact on swarming motility. These phenotypes (biofilm formation and swarming motility) are linked, with both requiring coordinated communication between cells and the latter is critical in the initiation of the former.^40^ Interference with this highly complex behavior would result in a less competitive organism at the community level. Pyrones **8** and **9** abolished swarming activity in *B. atropheaus* on semisolid agar, while pyrones **1** and **7** also strongly suppressed swarming motility, although not to the same extent (Figure 4). Based on the kinetic growth profiles, the absence of swarming motility in the presence of **9** could simply be attributed to growth antagonism rather than specific interference with the multicellular behavior. However, the absence of swarming activity in the presence of other pyrone compounds indicates a more behavioral mechanism. The trend here is similar to that in the antibiofilm test, i.e., pyrones with long alkyl chains at C3 suppress swarming. However, in this case, the naturally occurring **PPYA** (**1**) trended with the analogues.

**Scheme 2:**
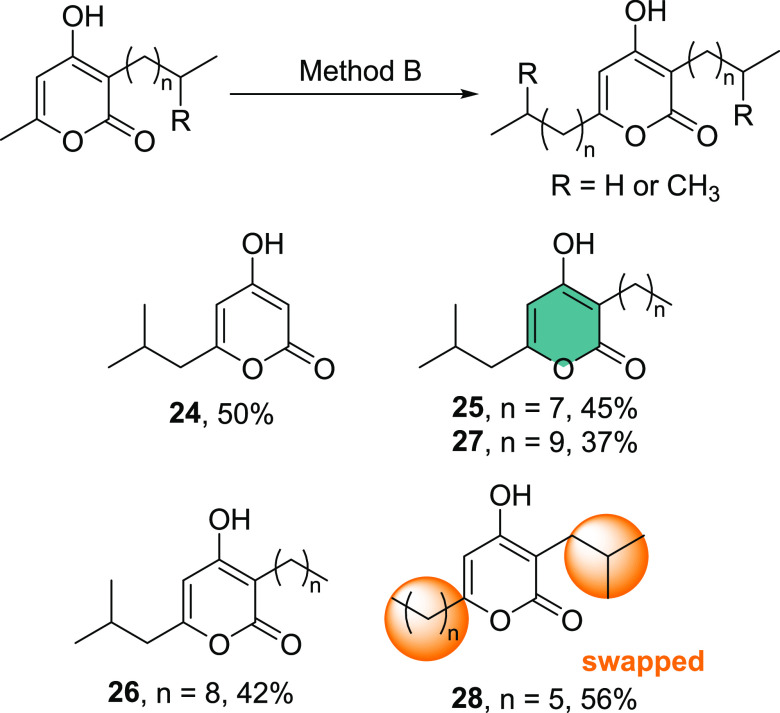
2-Pyrone Derivatives Synthesized for Further Structure–Activity Relationship Studies Method B: (i) TMEDA (1.0 equiv), THF/HMPA (5:1), *n*-BuLi (2.4 equiv), 0 °C, N_2_ and (ii) alkyl iodide (1.8 equiv), 0 °C–rt, 16 h (see the SI for details).

In terms of biofilm formation, the simpler synthetic pyrones discussed so far, possessing a methyl group at C6, showed contrasting effects on biofilm formation, relative to the naturally occurring **PPYA** signal (with an *i*-Bu group at C6). Even compound **5**, which is otherwise identical, gave a dramatically different phenotype. Thus, we needed to ascertain the importance of the C6 group. First, we synthesized the C6-*i*-Bu compound **24**, without any alkyl group at C3, to examine the properties of this analogue (Scheme 2). We then took the best performing C3-alkylated derivatives, **7**, **8**, and **9**, and reacted them with 2-iodopropane to give compounds **25** (native, **PPYC**), **26** (non-native), and **27** (native, **PPYE**). Finally, we reversed the positions of the alkyl chains present in **PPYA**, placing the *i*-Bu group at C3 and the *n-*hexyl group at C6 (**28**). The compounds were then tested for their impact on biofilm formation and growth of *B. atropheaus* as described above.

**Figure 5:**
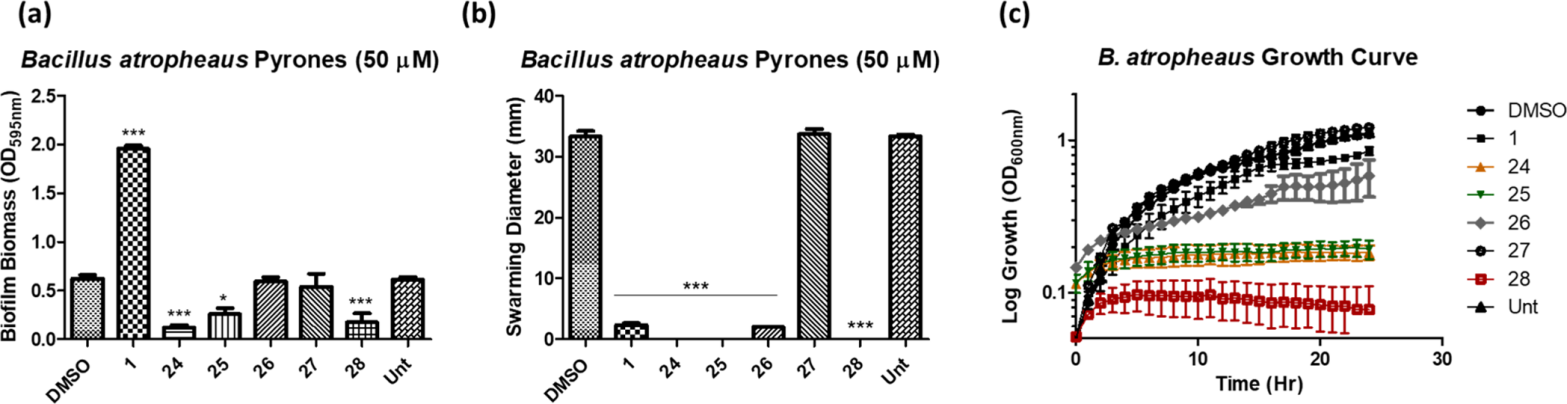
(a) Biofilm formation of B. atropheaus in the presence of derivative compounds **24**–**28**. (b) Swarming motility of B. atropheaus in the presence of derivative compounds **24**–**28**. (c) Growth curve analysis of B. atropheaus in the presence of derivative compounds **24**–**28** (see the SI for details). All data presented are the average (±SEM) of at least three independent biological replicates. Statistical analysis was performed by one-way ANOVA with Bonferroni post hoc corrective testing (*p ≤ 0.05, ***p ≤ 0.001).

While **1** again led to a notable increase in biofilm formation in *B. atropheaus*, none of the derivative compounds retained this activity (Figure 5a). Swarming motility was suppressed in the presence of **26** to the same extent as with **1** but was unaffected in the presence of **27**, which only differs by a CH_2_ group (Figure 5b). This is also consistent with the growth kinetics data, and thus, of the pyrone signals and derivative compounds tested, **26** was the only one that achieved swarming suppression activity comparable to **1**. However, it should be noted that the growth kinetics of *B. atropheaus* was affected in the presence of **26**, with the organism failing to reach the growth rate or final biomass achieved in the presence of the DMSO control. Compounds **24**, **25**, and **28** led to the abolition of growth on the plate, there was no evidence of colony initiation from the point of inoculation (Figure 5c). Remarkably, the bioactivity of compound **1** toward *B. atropheaus* appears to be entirely specific to the exact structural arrangement. The activity of the naturally occurring compounds **25** and **27** with respect to cell-clumping in *Photorhabdus* is as yet unknown.^14^ The data presented here suggest that they may play a distinct signaling role from the other native pyrones.

## Conclusions

In conclusion, some 25 natural^41^ and unnatural pyrones were synthesized.^42−44^ The naturally (in *P. luminescens*) occurring **PPYA** compound **1** was shown to enhance biofilm formation in *B. atropheaus*. In direct contrast, replacement of the isobutyl group at the C6 position with a simple methyl group gave compounds that induced antibiofilm activity. Indeed, antibiofilm activity was also noted for all other compounds, even those with an isobutyl group at C6 and differing from **1** by ±CH_2_ in the alkyl chain. Swarming inhibition activity appeared to require less structural specificity compared to the biofilm activity, although a requirement for specific chain length at C3 did emerge as critical for inhibition.

The mechanistic basis of the behavioral changes identified in response to the pyrone signals and their derivatives remains to be elucidated. A LuxR-type receptor–ligand interaction would be complex and likely multifaceted, considering the varied impacts of the PPY derivatives synthesized when compared to the native **PPYA** signal.

QS plays a role in biofilm development and swarming motility in *B. subtilis*,^33,45,46^ with AI-2 signaling recently reported eliciting a biofilm-dependent response through the LuxS receptor under specific environmental conditions.^47^ A LuxR-type receptor–ligand interaction, such as that described for the pyrones in *Photorhabdus*, would equally be complex and likely multifaceted. The activity and specificity of the natural pyrone signal **1** suggest that pyrones may have an interspecies communication role similar to that seen for the alkyl hydroxyquinolone signal molecules, HHQ and PQS, in *P. aeruginosa*, which also display similar specific structure–activity relationships.^20,21,26^ It is also worth noting that both HHQ and PQS modulate the behavior of a broad range of co-colonizing pathogens, yet the MvfR/PqsR receptor for these two QS molecules has not yet been identified outside of *P. aeruginosa*. The pyrone signal and derivatives presented in this report showed interspecies activity in the low micromolar range, eliciting a dose-dependent phenotypic response at 50 μM, in contrast to the nM range of endogenous signal activity observed in *P. luminescens*.^14^ This is consistent with the activity range of the HHQ and PQS signals, which are active in the nM range against their native receptor (PqsR) in *P. aeruginosa*,^23^ yet require concentrations in the 10–100 μM range to elicit an interspecies response.^20,21,26^ An important consideration here is the degree to which these compounds are naturally soluble in assays or indeed within the particular microbial community or ecosystem within which they are found. As an analogy, the solubility of both HHQ and PQS is significantly enhanced by endogenous biosurfactants called rhamnolipids, which are produced by *P. aeruginosa*.^48^ It is unclear as yet whether a similar phenomenon underpins the biological activity of photopyrones at the interspecies level as reported here, or indeed their interaction with solo LuxR receptor proteins.

Deciphering the breadth of signal-mediated interactions that underpins microbial communication is an important endeavor as we attempt to understand the “community networks” that sustain microbiomes in health and disease.^49^ The pyrone derivative described here may have applications against other pathogenic bacteria, which possess a LuxR solo protein, and this will be the focus of further studies. When this is the case, an extensive investigation of pyrone production in microbial communities would be warranted, particularly in light of the network of orphan LuxR proteins that exist in pathogenic organisms. The key finding of this study, the capacity for pyrones to elicit behavioral changes in a Gram-positive organism at a low micromolar concentration, points to the role of this new class of molecular signal in the interspecies interactome. Elucidating that role, and the extent to which it governs virulence and pathogenesis in competing organisms will require an interdisciplinary approach.

## Experimental Section

### Biology

#### Biofilm Formation Assays

*B. atropheaus* was inoculated from −80 °C stock onto tryptic soy agar (TSA) and incubated at 30 °C overnight. A colony was inoculated into 5 mL of tryptic soy broth (TSB) and incubated with shaking overnight at 30 °C. The culture was transferred to fresh TSB media with a starting OD_600nm_ of 0.05, combined with either a pyrone compound or DMSO control, and added to multiwell plates (200 μL into 96-well plates, 1 mL into 24-well plates). The plates were incubated static overnight at 30 °C and developed using the crystal violet assay as described previously.^22^

#### Swarming Motility Assays

Motility agar plates consisting of TSB with 0.3% w/v agar were allowed to air dry in a laminar flow for 30 min, after which time a fresh colony of *B. atropheaus* grown on TSA was spotted onto the center of the motility plate using a pipette tip. The plates were incubated statically at 30 °C and the zonal swarming diameter was measured. To facilitate screening of a large number of compounds, 6-well plates (Sarstedt), with each well containing 4 mL of motility agar, were used. Carrier and untreated controls were included in all experiments.

#### Growth Kinetics Assays

*B. atropheaus* was prepared for growth analysis following the protocol described for biofilm formation (vide supra). Once transferred into fresh TSB at a starting OD_600nm_ of 0.05, 200 μL was inoculated into a honeycomb plate and placed on the BioScreen C reader. Measurements (OD_600nm_) were taken every 30 min, with shaking for 10 s prior to data capture. DMSO and carrier controls were included in each experiment.

### Chemistry

#### General Considerations

Solvents and reagents were used as obtained from commercial sources and without purification, with the exception of THF, which was freshly distilled from sodium/benzophenone under nitrogen. All syntheses and spectra were run at the University College Cork. Melting points were measured in the Thomas Hoover Capillary Melting Point apparatus. Infrared spectra were recorded on a PerkinElmer Fourier transform infrared (FT-IR) spectrometer as thin films in dichloromethane (DCM). Column chromatography was carried out using 60 Å (35–70 μm) silica. Thin-layer chromatography (TLC) was carried out on precoated silica gel plates (Merck 60 PF254) and the developed plates were visualized under UV light. High-resolution precise mass spectra (HRMS) were recorded on a Waters LCT Premier time-of-flight liquid chromatography–mass spectrometry (TOF LC–MS) instrument in University College Cork. Samples were run in the electrospray ionization (ESI) mode using 50% acetonitrile–water containing 0.1% formic acid as the eluent; the samples were made up to a concentration of ca. 1 mg/mL. Nuclear magnetic resonance (NMR) samples were run in deuterated chloroform (CDCl_3_), deuterated dimethyl sulfoxide ((CD_3_)_2_SO), or deuterated methanol (CD_3_OD), as specified. ^1^H-NMR (500 MHz) and ^1^H-NMR (300 MHz) spectra were recorded on Bruker Avance 500 and Bruker Avance III 300 NMR spectrometers, respectively, in the proton coupled mode using tetramethylsilane (TMS) as the internal standard. ^13^C-NMR (125 MHz) and ^13^C-NMR (75 MHz) spectra were recorded on Bruker Avance 500 and Bruker Avance III 300 NMR spectrometers, respectively, in proton decoupled mode at 300 K using TMS as the internal standard. Chemical shifts (δ) are expressed as parts per million (ppm), positive shift being downfield from TMS; coupling constants (*J*) are expressed in hertz (Hz). Splitting patterns in ^1^H-NMR spectra are designated as s (singlet), bs (broad singlet), d (doublet), dd (doublet of doublets), dt (doublet of triplets), t (triplet), and m (multiplet). Elemental analysis was performed at the Microanalysis Laboratory, National University of Ireland, Cork, using PerkinElmer 240 and Exeter Analytical CE440 elemental analyzers.

#### Representative Procedure for Reductive Alkylation at C3 of 2-Pyrones

To a round bottom flask in open air was added 2-pyrone (1.0 equiv), the corresponding aldehyde (3.0 equiv), diethyl 1,4-dihydro-2,6-dimethyl-3,5-pyridinedicarboxylate (1.2 equiv), and DCM (15 mL/mmol). l-Proline (20 mol %) was then added and the sides of the flask were rinsed again with DCM. The resulting reaction mixture was allowed to stir vigorously for 16 h at rt. The reaction mixture was then concentrated under reduced pressure. The crude product was purified by column chromatography (hexane/EtOAc: 9:1 to 7:3).^36^

#### Representative Procedure for Electrophilic Substitution at C7 of 6-Alkyl-4-hydroxy-2*H*-pyran-2-ones

##### Method A

A Schlenk tube was heated under vacuum and refilled with N_2_ three times. 2-Pyrone (1.0 equiv) and HMDS (3 mL/mmol) were added, and the resulting reaction mixture was heated to 80 °C under N_2_ for 1 h. The solution was allowed to cool and HMDS was removed under reduced pressure. THF (3 mL/mmol) was then added, and the solution was cooled to −78 °C. *n*-BuLi (1.25 equiv) was added carefully over 15 min, and the solution was stirred for 1 h. Alkyl bromide (2.3 equiv) was then added over 10 min and the solution was allowed to warm gradually to rt, and then stirred for 16 h. The reaction was then quenched with 6 M HCl until pH ∼ 2 and the solvent was concentrated under reduced pressure. The residual mass was dissolved in ethyl acetate (10 mL) and washed with brine (2 × 10 mL). The combined organic extracts were dried over MgSO_4_, filtered, and concentrated under reduced pressure. The crude product was purified by column chromatography (hexane/EtOAc: 1:1).^50^

##### Method B

A Schlenk tube was heated under vacuum and refilled with N_2_ three times. 2-Pyrone (1.0 equiv) was added, followed by THF (2.77 mL/mmol), and the resulting white suspension was stirred at rt for 5 min. TMEDA (1.0 mmol, 1.0 equiv) and HMPA (0.55 mL/mmol) were added, and the resulting pale-yellow reaction mixture was cooled to 0 °C for 30 min. *n*-BuLi (2.4 equiv) was added dropwise for over 10 min giving a deep red reaction mixture that was stirred further for 1 h at 0 °C, followed by the dropwise addition of the corresponding alkyl iodide (1.8 equiv). The orange reaction mixture was warmed to rt and stirred under N_2_ for 16 h. The reaction was acidified with 4 M HCl until pH ∼ 2–3 and was then extracted with Et_2_O (3 × 10 mL). The combined organic layers were washed with H_2_O (3 × 10 mL) and brine (10 mL), dried over MgSO_4_, filtered, and concentrated under reduced pressure. The crude product was purified by column chromatography (hexane/EtOAc: 7:3).^36^

#### Representative Procedure for the Synthesis of 6-Alkyl-4-hydroxy-pyridin-2(1*H*)-ones

The corresponding pyrone (1.0 equiv), ammonia (5 mL/mmol), and water (2 mL/mmol) were heated at 130 °C for 6 h. The reaction was cooled to rt and diluted with water (4 mL/mmol). The mixture was acidified to pH ∼ 1 with 0.5 M HCl and the resulting precipitate was filtered and dried under vacuum.^51^

## Supporting Information Available

Characterization data of all compounds, supplementary figures and tables and copies of NMR spectra (PDF)
