# Supplementary material for: Pyrones Identified as LuxR Signal Molecules in Photorhabdus and Their Synthetic Analogues Can Alter Multicellular Phenotypic Behavior of Bacillus atropheaus
Source: ACS Omega. 2021 Nov 22;6(48):33141–8. doi: 10.1021/acsomega.1c05508 (PMC8655920; doi:10.1021/acsomega.1c05508)
Supplement: Supplementary file 1 — ao1c05508_si_001.pdf [file ao1c05508_si_001.pdf]

Supporting Information for:

Pyrones Identified as LuxR Signal Molecules in *Photorhabdus* and Their Synthetic Analogues Can Alter Multicellular Phenotypic Behavior of *Bacillus atropheaus*

Aobha Hickey,[a] Leticia M. Pardo,[a] F. Jerry Reen*[b,c] and Gerard P. McGlacken*[a,c]

[a]School of Chemistry, Analytical and Biological Chemistry Research Facility, [b]School of Microbiology, [c]Synthesis and Solid State Pharmaceutical Centre, University College Cork, Ireland.

**g.mcglacken@ucc.ie, *j.reen@ucc.ie*

Contents

Synthesis and Characterisation of 2-Pyrones and 2-Pyridones S2

Biological Figures and Tables S11

^{1}H and ^{13}C NMR Spectra S14

References S42

Synthesis and Characterisation of 2-Pyrones and 2-Pyridones

Synthesis of 3-alkyl-4-hydroxy-6-methyl-2*H*-pyran-2-ones

Representative procedure for reductive alkylation at C3 of 2-pyrones

To a round bottom flask in open air was added 2- pyrone (1.0 equiv.), corresponding aldehyde (3.0 equiv.), diethyl 1,4-dihydro-2,6-dimethyl-3,5-pyridinedicarboxylate (1.2 equiv.) and DCM (15 mL/mmol). *L*-proline (20 mol%) was then added and the sides of the flask were rinsed again with DCM. The resulting reaction mixture was allowed to stir vigorously for 16 h at r.t. The reaction mixture was then concentrated under reduced pressure. The crude product was purified by column chromatography (hexanes:EtOAc 9:1 to 7:3).[1]

4-Hydroxy-6-methyl-3-propyl-2*H*-pyran-2-one (2)[1]

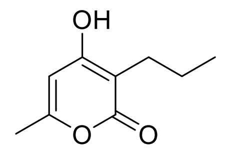

White crystalline solid (0.035 g, 41%); m.p. 148–151 °C (lit.[1] 148–151 °C); ^{1}H NMR (300 MHz, $CDCl_3$) δ: 6.20 (s, 1H), 2.50–2.35 (m, 2H), 2.22 (s, 3H), 1.63–1.43 (m, 2H), 0.94 (t, *J* = 7.4 Hz, 3H) ppm; ^{13}C NMR (75 MHz, $CDCl_3$) δ: 168.1, 166.9, 159.9, 103.1, 101.5, 24.9, 21.3, 19.7, 13.9 ppm; *m/z* (ES+): 169 ($(M+H)^+$ 100%).

3-Butyl-4-hydroxy-6-methyl-2*H*-pyran-2-one (3)

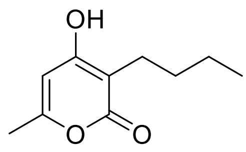

White crystalline solid (0.045 g, 49%); m.p. 130–133 °C (lit.[2] 132–133 °C); IR (film) ν_{max} 3583, 1650, 1575, 1447, 1292, 1127 cm^{-1}; ^{1}H NMR (300 MHz, $CDCl_3$) δ: 9.73 (bs, 1H), 6.18 (s, 1H), 2.56–2.35 (m, 2H), 2.22 (s, 3H), 1.61–1.20 (m, 4H), 0.91 (t, *J* = 7.2 Hz, 3H) ppm; ^{13}C NMR (75 MHz, $CDCl_3$) δ: 168.5, 167.5, 159.8, 103.4, 101.8, 30.2, 22.74, 22.66, 19.6, 14.0 ppm; HRMS (ESI-TOF) *m/z*: $[M+H]^+$ calcd for $C_{10}H_{15}O_3$: 183.1016; found: 183.1010.

4-Hydroxy-6-methyl-3-pentyl-2*H*-pyran-2-one (4)

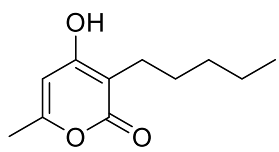

Off-white solid (0.179 g, 91%); m.p. 132–136 °C; IR (film) ν_{max} 3208, 1648, 1573, 1447, 1274, 1128 cm^{-1}; ^{1}H NMR (300 MHz, $CDCl_3$) δ: 10.60 (bs, 1H), 6.27 (s, 1H), 2.55–2.35 (m, 2H), 2.22 (s, 3H), 1.59–

1.20 (m, 6H), 0.87 (t, *J* = 6.9 Hz, 3H) ppm; ^{13}C NMR (75 MHz, $CDCl_3$) δ: 168.6, 167.7, 159.8, 103.4, 101.9, 31.8, 27.8, 23.0, 22.6, 19.7, 14.1 ppm; HRMS (ESI-TOF) *m/z*: $[M+H]^+$ calcd for $C_{11}H_{17}O_3$: 197.1211; found: 197.1208.

3-Hexyl-4-hydroxy-6-methyl-2*H*-pyran-2-one (5)[1]

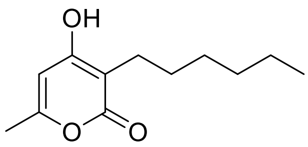

White solid (0.376 g, 90%); m.p. 115-118 °C (lit.[1] 120–121 °C); ^{1}H NMR (300 MHz, $CDCl_3$) δ: 10.52 (bs, 1H), 6.27 (s, 1H), 2.55–2.36 (m, 2H), 2.22 (s, 3H), 1.61–1.18 (m, 8H), 0.86 (t, *J* = 6.6 Hz, 3H) ppm; ^{13}C NMR (75 MHz, $CDCl_3$) δ: 168.6, 167.6, 159.8, 103.4, 101.9, 31.8, 29.3, 28.0, 23.0, 22.7, 19.6, 14.1 ppm; *m/z* (ES-): 209 ($(M-H)^-$ 100%).

3-Heptyl-4-hydroxy-6-methyl-2*H*-pyran-2-one (6)[1]

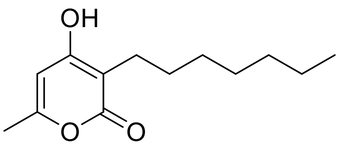

Off-white solid (0.210 g, 93%); m.p. 119–124 °C; ^{1}H NMR (300 MHz, $CDCl_3$) δ: 10.38 (bs, 1H), 6.24 (s, 1H), 2.50–2.41 (m, 2H), 2.22 (s, 3H), 1.60–1.14 (m , 10H), 0.86 (t, *J* = 6.7 Hz, 3H) ppm; ^{13}C NMR (75 MHz, $CDCl_3$) δ: 168.5, 167.6, 159.7, 103.9, 101.8, 31.9, 29.6, 29.3, 28.1, 23.0, 22.7, 19.6, 14.1 ppm; *m/z* (ES+): 225 ($(M+H)^+$ 16%).

4-Hydroxy-6-methyl-3-octyl-2*H*-pyran-2-one (7)[3]

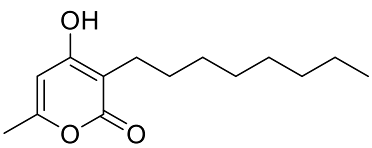

White solid (0.211 g, 88%); m.p. 103–105 °C (lit.[3] 103–104.5 °C); ^{1}H NMR (300 MHz, $CDCl_3$) δ: 10.47 (bs, 1H), 6.25 (s, 1H), 2.56–2.34 (m, 2H), 2.22 (s, 3H), 1.61–1.15 (m, 12H), 0.86 (t, *J* = 6.7 Hz, 3H) ppm; ^{13}C NMR (75 MHz, $CDCl_3$) δ: 168.5, 167.5, 159.7, 103.4, 101.8, 31.9, 29.7, 29.6, 29.3, 28.1, 23.0, 22.7, 19.6, 14.1 ppm; *m/z* (ES+): 239 ($(M+H)^+$ 98%).

4-Hydroxy-6-methyl-3-nonyl-2*H*-pyran-2-one (8)

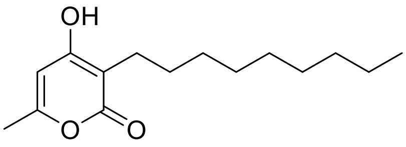

White solid (0.227 g, 90%); m.p. 93–96 °C; IR (film) ν_{max} 3410, 1670, 1594, 1448, 1249, 1129 cm^{-1}; ^{1}H NMR (300 MHz, $CDCl_3$) δ: 10.49 (bs, 1H), 6.26 (s, 1H), 2.55–2.32 (m, 2H), 2.22 (s, 3H), 1.61–1.11 (m, 14H), 0.86 (t, *J* = 6.7 Hz, 3H) ppm; ^{13}C NMR (75 MHz, $CDCl_3$) δ: 168.5, 167.5, 159.7, 103.4, 101.8, 31.9, 29.7 (2 × C), 29.6, 29.4, 28.1, 23.0, 22.7, 19.6, 14.1 ppm; HRMS (ESI-TOF) *m/z*: $[M+H]^+$ calcd for $C_{15}H_{25}O_3$: 253.1804; found: 253.1797; Anal. calcd for $C_{15}H_{24}O_3$: C, 71.39; H, 9.59; found: C, 71.53; H, 9.55.

3-Decyl-4-hydroxy-6-methyl-2*H*-pyran-2-one (9)[3]

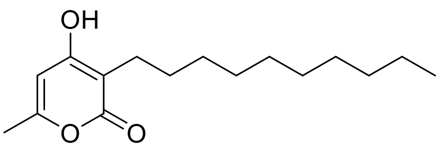

White solid (0.246 g, 92%); m.p. 103–105 °C (lit.[3] 101.5–102.5 °C); ^{1}H NMR (300 MHz, $CDCl_3$) δ: 10.73 (bs, 1H), 6.29 (s, 1H), 2.55–2.34 (m, 2H), 2.22 (s, 3H), 1.59–1.15 (m, 16H), 0.87 (t, J = 6.7 Hz, 3H) ppm; ^{13}C NMR (75 MHz, $CDCl_3$) δ: 168.6, 167.8, 159.7, 103.4, 101.9, 31.9, 29.71, 29.67 (2 × C), 29.64, 29.4, 28.1, 23.0, 22.7, 19.6, 14.1 ppm; *m/z* (ES+): 267 ($(M+H)^+$ 100%).

4-Hydroxy-3-isobutyl-6-methyl-2*H*-pyran-2-one (10)[1]

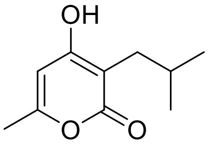

White solid (0.171 g, 94%); m.p. 144–148 °C (lit.[1] 143–148 °C); ^{1}H NMR (300 MHz, $CDCl_3$) δ: 10.34 (s, 1H), 6.18 (s, 1H), 2.28 (d, J = 7.3 Hz, 2H), 2.14 (s, 3H), 1.96–1.79 (m, 1H), 0.85 (d, J = 6.7 Hz, 6H) ppm; ^{13}C NMR (75 MHz, $CDCl_3$) δ: 168.8, 168.1, 159.9, 102.4, 101.8, 31.7, 27.5, 22.4 (2 × C), 19.6 ppm; *m/z* (ES+): 183 ($(M+H)^+$ 100%).

4-Hydroxy-3-isopentyl-6-methyl-2*H*-pyran-2-one (11)

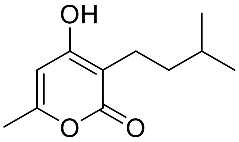

White crystalline solid (0.185 g, 94%); m.p. 149–155 °C; IR (film) ν_{max} 3429, 1669, 1578, 1450, 1249, 1131 cm^{-1}; ^{1}H NMR (300 MHz, $CDCl_3$) δ: 10.13 (bs, 1H), 6.22 (s, 1H), 2.50–2.37 (m, 2H), 2.22 (s, 3H), 1.68–1.49 (m, 1H), 1.47–1.29 (m, 2H), 0.92 (d, J = 6.6 Hz , 6H) ppm; ^{13}C NMR (75 MHz, $CDCl_3$) δ: 168.6, 167.6, 159.7, 103.6, 101.9, 37.0, 22.5 (2 × C), 21.8, 21.0, 19.7 ppm; HRMS (ESI-TOF) *m/z*: $[M+H]^+$ calcd for $C_{11}H_{17}O_3$: 197.1178; found: 197.1184.

3-Benzyl-4-hydroxy-6-methyl-2*H*-pyran-2-one (12)[1]

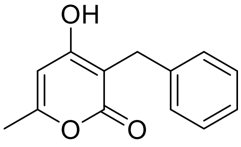

White solid (0.204 g, 94%); m.p. 167–170 °C (lit.[1] 156–158 °C); ^{1}H NMR (300 MHz, CD_3OD) δ: 7.29–7.01 (m, 5H), 6.02 (s, 1H), 3.69 (s, 2H), 2.19 (s, 3H) ppm; ^{13}C NMR (75 MHz, CD_3OD) δ: 168.5, 168.0, 162.3, 141.7, 129.4 (2 × C), 129.0 (2 × C), 126.8, 103.0, 101.4, 29.5, 19.5 ppm; *m/z* (ES+): 217 ($(M+H)^+$ 54%).

4-Hydroxy-6-methyl-3-(3-phenylpropyl)-2*H*-pyran-2-one (13)[4]

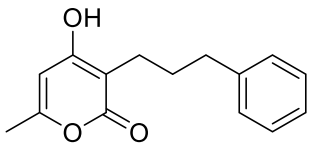

Off-white solid (0.220 g, 90%); m.p. 129–130 °C (lit.[4] 129–131 °C); ^{1}H NMR (300 MHz, $CDCl_3$) δ: 10.70 (bs, 1H), 7.36–6.98 (m, 5H), 6.25 (s, 1H), 2.84–2.37 (m, 4H), 2.16 (s, 3H), 1.98–1.69 (m, 2H) ppm; ^{13}C NMR (75 MHz, $CDCl_3$) δ: 168.6, 167.8, 160.1, 142.5, 128.3 (2 × C), 128.2 (2 × C), 125.6, 102.9, 101.9, 35.9, 29.7, 23.1, 19.7 ppm; *m/z* (ES+): 245 ($(M+H)^+$ 100%).

4-Hydroxy-6-methyl-3-(2-phenylpropyl)-2*H*-pyran-2-one (14)

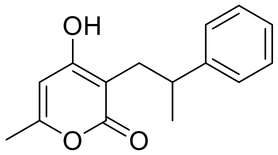

White solid (0.223 g, 91%); m.p. 184–186 °C; IR (film) ν_{max} 3389, 1641, 1575, 1445, 1257, 1122 cm^{-1}; ^{1}H NMR (300 MHz, $CD_3OD/CDCl_3$) δ: 7.34–7.05 (m, 5H), 5.90 (d, *J* = 0.8 Hz, 1H), 3.22–3.04 (m, 1H), 2.73–2.55 (m, 2H), 2.18 (d, *J* = 0.8 Hz, 3H), 1.23 (d, *J* = 7.0 Hz, 3H) ppm; ^{13}C NMR (75 MHz, $CD_3OD/CDCl_3$) δ: 168.4, 167.7, 161.0, 148.0, 128.6 (2 × C), 127.6 (2 × C), 126.4, 102.0, 101.2, 38.8, 32.3, 21.3, 19.7 ppm; HRMS (ESI-TOF) *m/z*: $[M+H]^+$ calcd for $C_{15}H_{17}O_3$: 245.1178; found: 245.1170.

4-Hydroxy-6-methyl-3-(pyridin-3-ylmethyl)-*2H*-pyran-2-one (15)

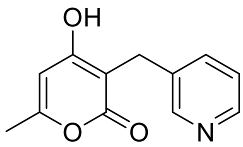

Compound **10** precipitated out of the reaction mixture, was collected *via* suction filtration and recrystallised from hot DCM/MeOH to yield a white crystalline solid (0.076 g, 35%); m.p. 202–205 °C; IR (film) ν_{max} 3382, 1683, 1661, 1587, 1448, 1270, 1120 cm^{-1}; ^{1}H NMR (300 MHz, $(CD_3)_2SO$) δ: 8.54–8.24 (m, 2H), 7.57 (dt, *J* = 7.8, 1.9 Hz, 1H), 7.26 (dd, *J* = 7.8, 4.8 Hz, 1H), 6.04 (s, 1H), 3.59 (s, 2H), 2.16 (s, 3H) ppm; ^{13}C NMR (75 MHz, $(CD_3)_2SO$) δ: 166.4, 165.2, 161.2, 149.9, 147.5, 136.6, 136.0, 123.8, 100.3, 100.1, 26.2, 19.8 ppm; HRMS (ESI-TOF) *m/z*: $[M+H]^+$ calcd for $C_{12}H_{12}NO_3$: 218.0817; found: 218.0810; Anal. calcd for $C_{12}H_{11}NO_3$: C, 66.35; H, 5.10; N, 6.45; found: C, 65.06; H, 5.18; N, 6.73.

3-(Furan-3-ylmethyl)-4-hydroxy-6-methyl-2*H*-pyran-2-one (16)

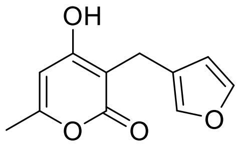

Beige crystalline solid (0.175 g, 85%); m.p. 187–189 °C; IR (film) ν_{max} 3401, 1663, 1619, 1572, 1448, 1262, 1124, 1018 cm^{-1}; ^{1}H NMR (300 MHz, $(CD_3)_2SO$) δ: 11.29 (bs, 1H), 7.48 (t, *J* = 1.6 Hz, 1H), 7.34 (d, *J* = 0.5 Hz, 1H), 6.31 (d, *J* = 0.9 Hz, 1H), 6.02 (d, *J* = 0.7 Hz, 1H), 3.37 (s, 2H), 2.15 (s, 3H) ppm;

^{13}C NMR (75 MHz, $(CD_3)_2SO$) δ: 165.3, 164.6, 160.3, 142.7, 139.1, 123.1, 111.4, 100.0, 99.8, 19.3, 18.1 ppm; HRMS (ESI-TOF) *m/z*: $[M+H]^+$ calcd for $C_{11}H_{11}O_4$: 207.0657; found: 207.0653.

3-(Furan-2-ylmethyl)-4-hydroxy-6-methyl-2*H*-pyran-2-one (17)

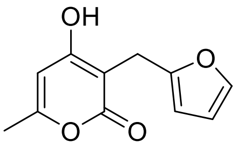

Yellow solid (0.194 g, 93%); m.p. 172–174 °C (lit.[2] 177–178 °C); IR (film) ν_{max} 3380, 1664, 1621, 1573, 1449, 1263, 1125, 1020 cm^{-1}; ^1H NMR (300 MHz, $(CD_3)_2SO$) δ: 7.43 (dd, *J* = 1.8, 0.8 Hz, 1H), 6.29 (dd, *J* = 3.1, 1.9 Hz, 1H), 6.03 (d, *J* = 0.9 Hz, 1H), 5.92 (dd, *J* = 3.1, 0.9 Hz, 1H), 3.58 (s, 2H), 2.16 (d, *J* = 0.7 Hz, 3H) ppm; ^{13}C NMR (75 MHz, $(CD_3)_2SO$) δ: 166.0, 164.4, 160.7, 153.3, 140.9, 110.3, 105.0, 99.8, 97.3, 21.7, 19.3 ppm; HRMS (ESI-TOF) *m/z*: $[M+H]^+$ calcd for $C_{11}H_{11}O_4$: 207.0657; found: 207.0656.

Synthesis of 6-alkyl-4-hydroxy-2*H*-pyran-2-ones

Representative procedure for electrophilic substitution at C7 of 6-alkyl-4-hydroxy-2H-pyran-2-ones

Method A: A Schlenk tube was heated under vacuum and refilled with N_2 three times. 2-Pyrone (1.0 equiv.) and HMDS (3 mL/mmol) were added, and the resulting reaction mixture was heated to 80°C under N_2 for 1 hour. The solution was allowed to cool and the HMDS removed under reduced pressure. THF (3 mL/mmol) was then added, and the solution cooled to -78°C. *n*-BuLi (1.25 equiv.) was added carefully over 15 min., and the solution stirred for 1 hour. Alkyl bromide (2.3 equiv.) was then added over 10 min. and the solution allowed to warm gradually to r.t., then stirred for 16 hours. The reaction was then quenched with 6 M HCl until pH ~ 2 and the solvent was concentrated under reduced pressure. The residual mass was dissolved in ethyl acetate (10 mL) and washed with brine (2 × 10 mL). The combined organic extracts were dried over MgSO4, filtered and concentrated under reduced pressure. The crude product was purified by column chromatography (hexanes:EtOAc 1:1).[5]

4-Hydroxy-6-octyl-2*H*-pyran-2-one (20)[6]

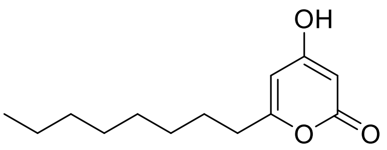

White solid (0.046 g, 10%); m.p. 56–59 °C (lit.[6] 57–59 °C); ^1H NMR (300 MHz, $CDCl_3$) δ: 10.91 (br s, 1H), 5.97 (d, *J* = 1.5 Hz, 1H), 5.57 (d, *J* = 1.8 Hz, 1H), 2.47 (t, *J* = 7.5 Hz, 2H), 1.64

(t, J = 7.2 Hz, 2H), 1.41-1.14 (m, 10H), 0.88 (t, J = 6.7 Hz, 3H). ppm; ^{13}C NMR (75 MHz, $CDCl_3$) δ: 172.5, 168.2, 167.5, 101.2, 89.8, 33.7, 31.8, 29.2, 29.1, 28.9, 26.7, 22.6, 14.1 ppm; *m/z* (ES-): 223 ($(M-H)^-$ 14%).

4-Hydroxy-6-nonyl-2*H*-pyran-2-one (21)[7]

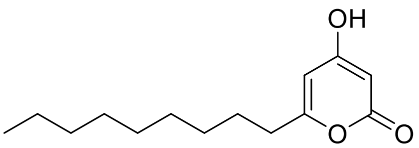

White solid (0.060 g, 13%); m.p. 79–80 °C (lit.[7] 78–79 °C); ^{1}H NMR (300 MHz, $CDCl_3$) δ: 10.83 (br s, 1H), 5.97 (d, J = 1.3 Hz, 1H), 5.58 (d, J = 1.7 Hz, 1H), 2.47 (t, J = 7.4 Hz, 2H), 1.64 (t, J = 7.4 Hz, 2H), 1.43–1.14 (m, 12H), 0.88 (t, J = 6.4 Hz, 3H) ppm; ^{13}C NMR (75 MHz, $CDCl_3$) δ: 172.4, 168.1, 167.5, 101.2, 89.8, 33.7, 31.8, 29.4, 29.2 (2 × C), 28.9, 26.7, 22.6, 14.1 ppm; *m/z* (ES-): 237 ($(M-H)^-$ 100%).

Method B: A Schlenk tube was heated under vacuum and refilled with N_2 three times. 2-Pyrone (1.0 equiv.) was added, followed by THF (2.77 mL/mmol), the resulting white suspension was stirred at r.t. for 5 min. TMEDA (1.0 mmol, 1.0 equiv.) and HMPA (0.55 mL/mmol) were added, and the resulting pale-yellow reaction mixture was cooled to 0 °C for 30 min. *n*-BuLi (2.4 equiv.) was added dropwise over 10 min. giving a deep red reaction mixture that was stirred for a further 1 h at 0 °C, followed by the dropwise addition the corresponding alkyl iodide (1.8 equiv.). The orange reaction mixture was warmed to r.t. and stirred under N_2 for 16 h. The reaction was acidified with 4 M HCl until pH ~ 2–3 and was then extracted with Et_2O (3 × 10 mL). The combined organic layers were washed with H_2O (3 × 10 mL) and brine (10 mL), dried over $MgSO_4$, filtered and concentrated under reduced pressure. The crude product was purified by column chromatography (hexanes:EtOAc 7:3).[1]

3-Hexyl-4-hydroxy-6-isobutyl-2*H*-pyran-2-one (1)[1]

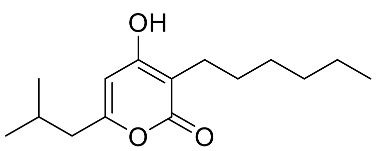

Pale yellow solid (0.062 g, 49%); m.p. 109–112 °C; ^{1}H NMR (300 MHz, $CDCl_3$) δ: 10.49 (bs, 1H), 6.21 (s, 1H), 2.58–2.21 (m, 4H), 2.15–1.94 (m, 1H), 1.55–1.22 (m, 8H), 1.01–0.75 (m, 9H) ppm; ^{13}C NMR (75 MHz, $CDCl_3$) δ: 168.4, 167.4, 162.6, 103.5, 102.0, 42.6, 31.8, 29.1, 28.0, 26.2, 23.1, 22.7, 22.2 (2 × C), 14.1 ppm; *m/z* (ES+): 253 ($(M+H)^+$ 100%); Anal. calcd. for $C_{15}H_{24}O_3$: C, 71.39; H, 9.59; found: C, 71.32; H, 9.39.

6-Hexyl-4-hydroxy-2*H*-pyran-2-one (18)[8]

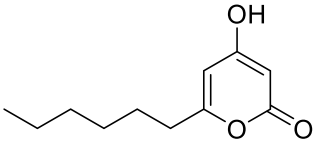

Yellow-orange solid (0.034 g, 17%); m.p. 44–46 °C (lit.[8] 56–57 °C); ^{1}H NMR (300 MHz, $CDCl_3$) δ: 6.00 (s, 1H), 5.58 (bs, 1H), 2.47 (t, *J* = 7.6 Hz, 2H), 1.76–1.53 (m, 2H), 1.40–1.19 (m, 6H), 0.88 (t, *J* = 6.7 Hz, 3H) ppm; ^{13}C NMR (75 MHz, $CDCl_3$) δ: 172.7, 168.4, 167.3, 101.3, 89.8, 33.6, 31.4, 28.6, 26.6, 22.4, 14.0 ppm; *m/z* (ES+): 197 ($(M+H)^+$ 70%).

6-Heptyl-4-hydroxy-2*H*-pyran-2-one (19)[9]

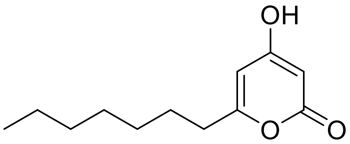

Yellow-orange solid (0.087 g, 41%); m.p. 68–70 °C (lit.[9] 72–73 °C); ^{1}H NMR (300 MHz, $CDCl_3$) δ: 10.63 (bs, 1H), 6.01 (s, 1H), 5.60 (bs, 1H), 2.48 (t, *J* = 7.3 Hz, 2H), 1.80–1.50 (m, 2H), 1.43–1.15 (m, 8H), 0.87 (t, *J* = 6.4 Hz, 3H) ppm; ^{13}C NMR (75 MHz, $CDCl_3$) δ: 172.8, 167.3, 166.9, 101.4, 89.8, 33.6, 31.6, 28.9 (2 × C), 26.7, 22.6, 14.0 ppm; m/z (ES+): 211 ($(M+H)^+$ 100%).

4-Hydroxy-6-isobutyl-2*H*-pyran-2-one (24)[1]

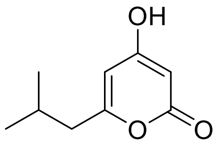

White solid (0.165 g, 58%); m.p. 103–106 °C (lit.[10] 106–108 °C); ^{1}H NMR (300 MHz, $CDCl_3$) δ: 11.13 (bs, 1H), 6.00 (d, *J* = 1.8 Hz, 1H), 5.60 (d, *J* = 1.8 Hz, 1H), 2.35 (d, *J* = 7.2 Hz, 2H), 2.15–1.94 (m, 1H), 0.95 (d, *J* = 6.6 Hz, 6H) ppm; ^{13}C NMR (75 MHz, $CDCl_3$) δ: 172.6, 168.5, 166.4, 102.4, 89.9, 42.8, 26.9, 22.2 (2 × C) ppm; *m/z* (ES-): 167 ($(M-H)^-$ 100%).

4-Hydroxy-6-isobutyl-3-octyl-2*H*-pyran-2-one (25)

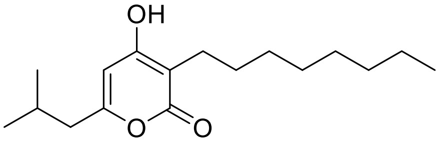

White solid (0.063 g, 45%); m.p. 105–107 °C; IR (film) ν_{max} 3089, 1663, 1569, 1433, 1273, 1130 cm^{-1}; ^{1}H NMR (300 MHz, $CDCl_3$) δ: 9.58 (bs, 1H), 6.13 (s, 1H), 2.57–2.37 (m, 2H), 2.30 (d, *J* = 7.2 Hz, 2H), 2.16–1.92 (m, 1H), 1.59–1.15 (m, 12H), 1.01–0.75 (m, 9H) ppm; ^{13}C NMR (75 MHz, $CDCl_3$) δ: 168.4, 167.1, 162.6, 103.5, 101.5, 42.6, 31.9, 29.7, 29.6, 29.4, 28.1, 26.9, 23.1, 22.7, 22.2 (2 × C), 14.1 ppm; HRMS (ESI-TOF) *m/z*: $[M+H]^+$ calcd. for $C_{17}H_{29}O_3$: 281.2111; found: 281.2113.

4-Hydroxy-6-isobutyl-3-nonyl-2*H*-pyran-2-one (26)

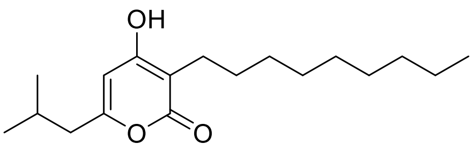

White solid (0.062 g, 42%); m.p. 98–100 °C; IR (film) ν_{max} 3044, 1663, 1569, 1433, 1265, 1130 cm^{-1}; ^1H NMR (300 MHz, $CDCl_3$) δ: 9.51 (bs, 1H), 6.12 (s, 1H), 2.57–2.37 (m, 2H), 2.30 (d, *J* = 7.2 Hz, 2H), 2.17–1.92 (m, 1H), 1.64–1.15 (m, 14H), 1.04–0.66 (m, 9H) ppm; ^{13}C NMR (75 MHz, $CDCl_3$) δ: 168.5, 167.3, 162.6, 103.5, 102.0, 42.6, 31.9, 29.74, 29.66 (2 × C), 29.4, 28.1, 26.9, 23.1, 22.7, 22.2 (2 × C), 14.1 ppm; HRMS (ESI-TOF) *m/z*: $[M+H]^+$ calcd. for $C_{18}H_{31}O_3$: 295.2268; found: 295.2268.

3-Decyl-4-hydroxy-6-isobutyl-2*H*-pyran-2-one (27)

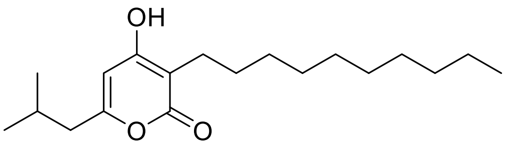

Pale yellow solid (0.057 g, 37%); m.p. 95–96 °C; IR (film) ν_{max} 3090, 1663, 1567, 1432, 1258, 1130 cm^{-1}; ^1H NMR (300 MHz, $CDCl_3$) δ: 9.08 (bs, 1H), 6.08 (s, 1H), 2.53–2.37 (m, 2H), 2.30 (d, *J* = 7.2 Hz, 2H), 2.16–1.96 (m, 1H), 1.60–1.12 (m, 16H), 0.99–0.79 (m, 9H) ppm; ^{13}C NMR (75 MHz, $CDCl_3$) δ: 168.4, 167.1, 162.6, 103.5, 101.9, 42.6, 31.9, 29.73, 29.71, 29.67, 29.66, 29.4, 28.1, 26.9, 23.1, 22.7, 22.2 (2 × C), 14.1 ppm; HRMS (ESI-TOF) *m/z*: $[M+H]^+$ calcd. for $C_{19}H_{33}O_3$: 309.2424; found: 309.2425.

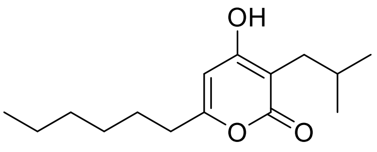

6-Hexyl-4-hydroxy-3-isobutyl-2*H*-pyran-2-one (28)

Pale yellow solid (0.071 g, 56%); m.p. 105–108 °C; IR (film) ν_{max} 3078, 1664, 1573, 1435, 1265, 1124 cm^{-1}; ^1H NMR (300 MHz, $CDCl_3$) δ: 10.01 (bs, 1H), 6.18 (s, 1H), 2.55–2.26 (m, 4H), 2.10–1.86 (m, 1H), 1.73–1.53 (m, 2H), 1.44–1.16 (m, 6H), 1.03–0.79 (m, 9H) ppm; ^{13}C NMR (75 MHz, $CDCl_3$) δ: 168.4, 167.5, 163.7, 102.4, 100.7, 33.5, 31.9, 31.4, 28.7, 27.5, 26.7, 22.45, 22.44 (2 × C), 14.0 ppm; HRMS (ESI-TOF) *m/z*: $[M+H]^+$ calcd. for $C_{15}H_{25}O_3$: 253.1798; found: 253.1800.

Synthesis of 6-alkyl-4-hydroxy-pyridin-2(1*H*)-ones

Representative procedure for the synthesis of 6-alkyl-4-hydroxy-pyridin-2(1H)-ones

The corresponding pyrone (1.0 equiv.), ammonia (5 mL/mmol) and water (2 mL/mmol) were heated at 130 °C for 6 hours. The reaction was cooled to r.t. and diluted with water (4 mL/mmol). The mixture was acidified to pH ~ 1 with 0.5 M HCl and the resulting precipitate was filtered and dried under vacuum.[11]

4-Hydroxy-6-octylpyridin-2(1*H*)-one (22)[12]

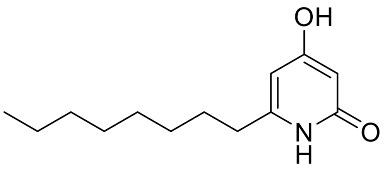

White solid (0.010 g, 67%); m.p. 174–176 °C; ^{1}H NMR (500 MHz, $(CD_3)_2SO$) δ: 10.86 (br s, 1H), 10.30 (br s, 1H), 5.58 (d, *J* = 2.2 Hz, 1H), 5.33 (d, *J* = 2.2 Hz, 1H), 2.33 (t, *J* = 7.6 Hz, 2H), 1.60-1.42 (m, 2H), 1.34-1.15 (m, 10H), 0.86 (t, *J* = 6.9 Hz, 3H) ppm; ^{13}C NMR (125 MHz, $(CD_3)_2SO$) δ: 167.9, 165.2, 150.5, 97.8, 96.4, 32.6, 31.7, 29.1, 29.0, 28.8, 28.5, 22.5, 14.4 ppm; *m/z* (ES+): 224 ($(M+H)^+$ 56%).

4-Hydroxy-6-nonylpyridin-2(1*H*)-one (23)[12]

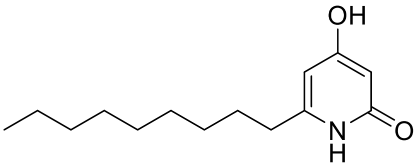

White solid (0.012 g, 60%); m.p. 168–170 °C; ^{1}H NMR (500 MHz, $(CD_3)_2SO$) δ: 10.87 (br s, 1H), 10.31 (br s, 1H), 5.59 (d, *J* = 1.9 Hz, 1H), 5.34 (d, *J* = 2.0 Hz, 1H), 2.35 (t, *J* = 7.6 Hz, 2H), 1.60–1.42 (m, 2H), 1.41–1.14 (m, 12H), 0.86 (t, *J* = 6.8 Hz, 3H) ppm; ^{13}C NMR (125 MHz, $(CD_3)_2SO$) δ: 167.9, 165.2, 150.5, 97.8, 96.4, 32.5, 31.7, 29.3, 29.11, 29.10, 28.8, 28.5, 22.5, 14.4 ppm; *m/z* (ES+): 238 ($(M+H)^+$ 82%).

Biological Figures and Tables

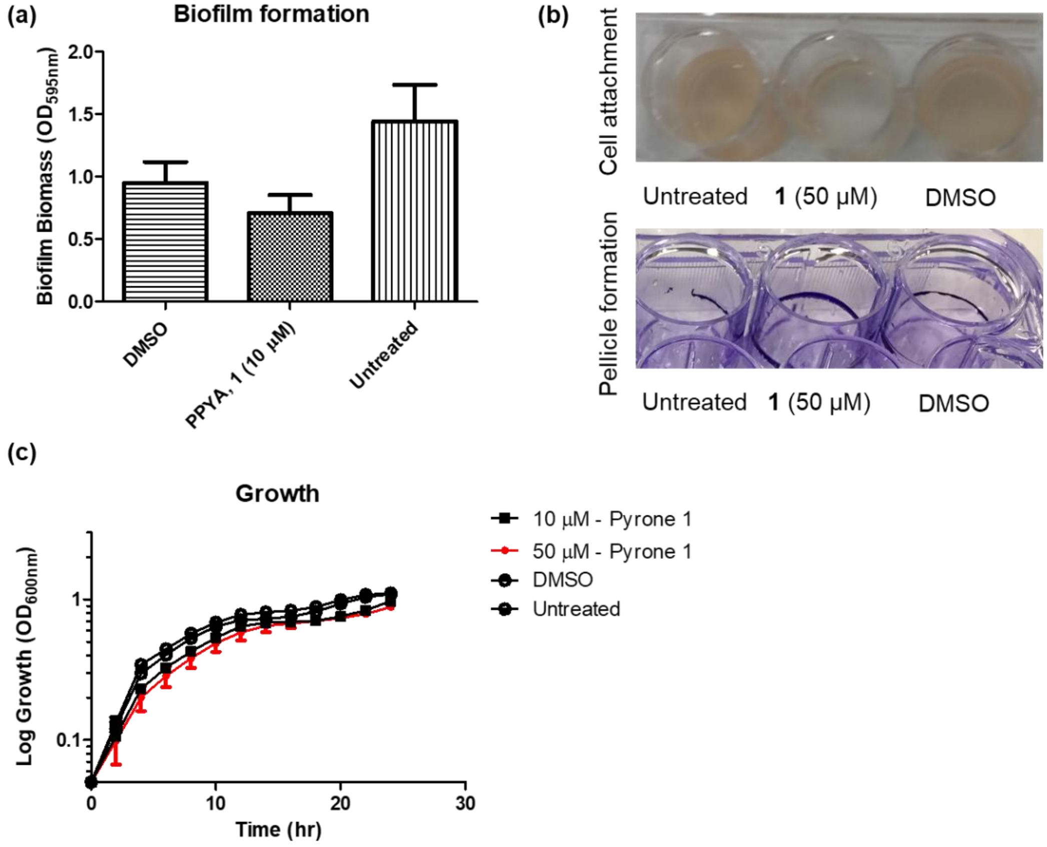

Figure S1 (a) *Biofilm formation of B. atropheaus in the presence of 10 µM* ***PPYA*** *or DMSO control. Data represents the average (+/- SEM) of three independent biological replicates.* ***(b)*** *Pellicle visualisation of B. atropheaus biofilms formed on 24-well plates in the presence of (left to right: untreated, 10 µM* ***PPYA****, DMSO) prior to quantification. Increased pellicle formation is evident in the* ***PPYA*** *wells when compared to the untreated and DMSO controls.* ***(c)*** *Growth kinetics of B. atropheaus in the presence of* ***PPYA*** *revealed no inhibitory activity suggesting that the phenotypes affected in the presence of this signal are the result of a mechanistic response.*

(a)

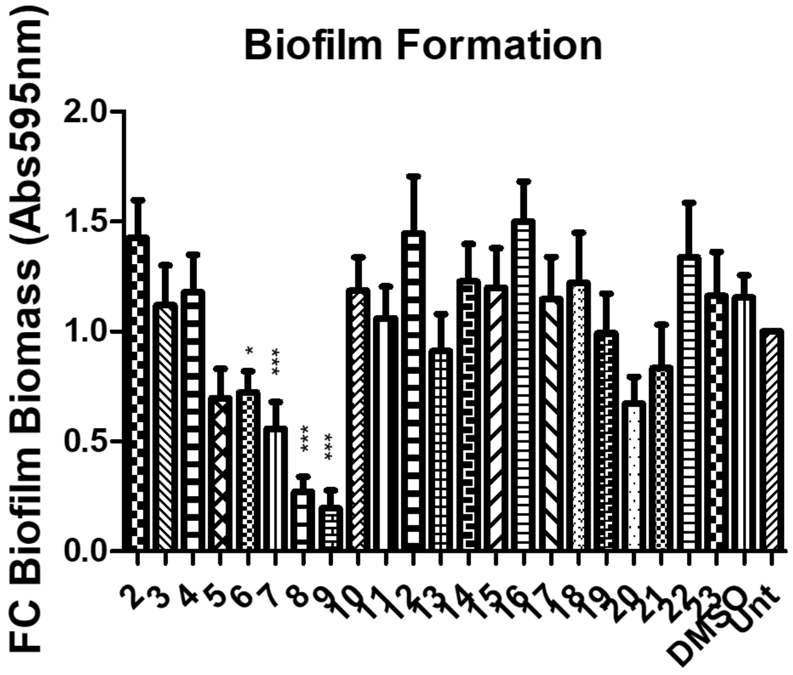

(b)

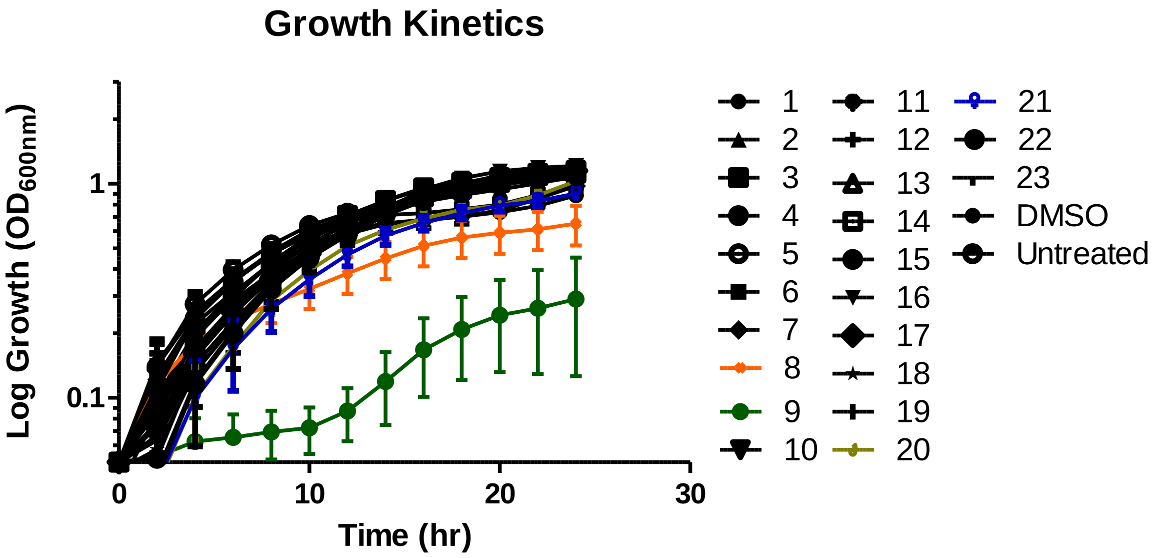

Figure S2 (a) *Biofilm formation of B. atropheaus in the presence of pyrone derivative compounds. Assays performed in 96-well microtitre plates. Data presented is the average (+/- SEM) of at least three independent biological replicates. Statistical analysis was performed by One way ANOVA with Bonferroni Multiple Comparison post-hoc corrective testing (* p≤0.05, *** p≤0.001).* ***(b)*** *Growth kinetics analysis of B. atropheaus in the presence of 50 µM pyrone signal or derivative compounds. Data presented is the average (+/- SEM) of three independent biological replicates.*

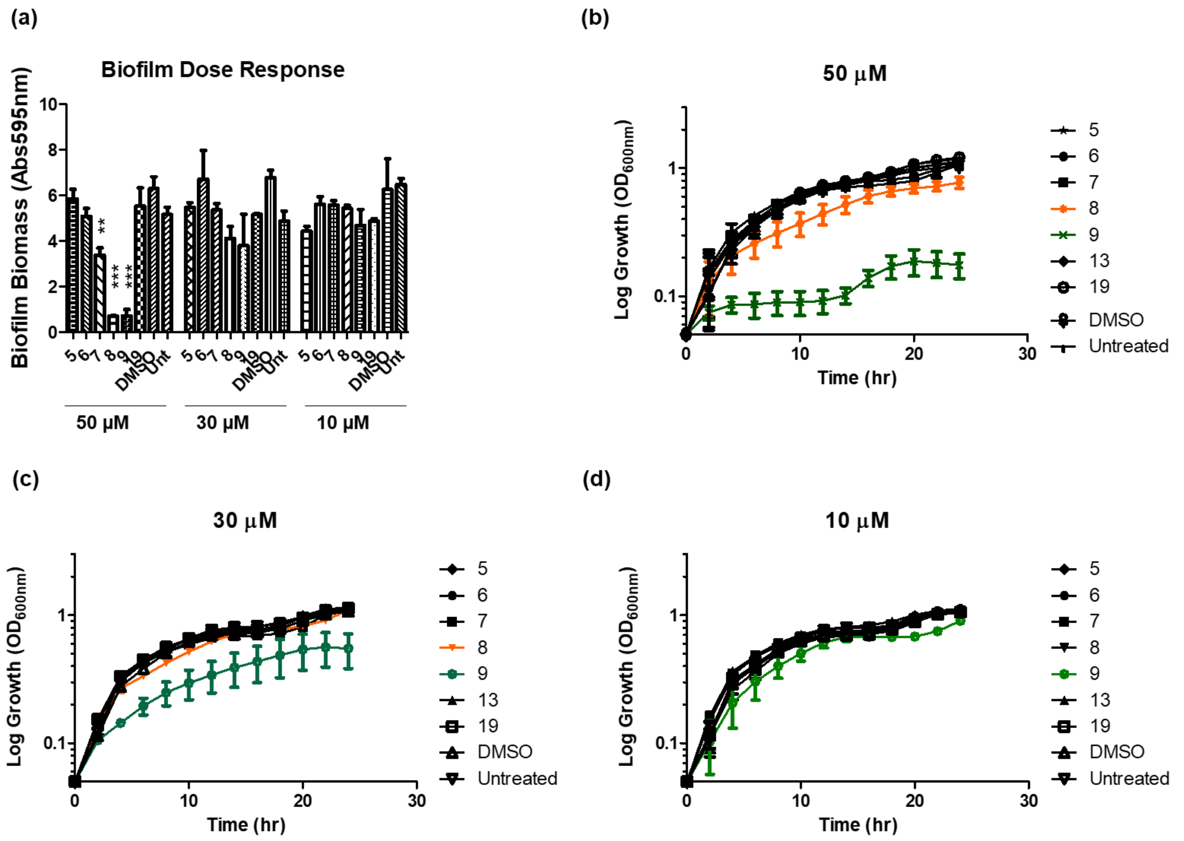

Figure S3 (a) *Biofilm dose response of B. atropheaus in the presence of 50, 30, or 10 µM pyrone derivatives. Data indicates a dose dependent response whereby antibiofilm activity is lost below a concentration of 50 µM.* ***(b-d)*** *Growth kinetic analysis of B. atropheaus in the presence of 50, 30, or 10 µM pyrone derivatives. All data presented is the average (+/- SEM) of at least three independent biological replicates. Statistical analysis was performed by One way ANOVA with Bonferroni Multiple Comparison post-hoc corrective testing (** p≤0.005, *** p≤0.001).*

^{1}H and ^{13}C NMR Spectra

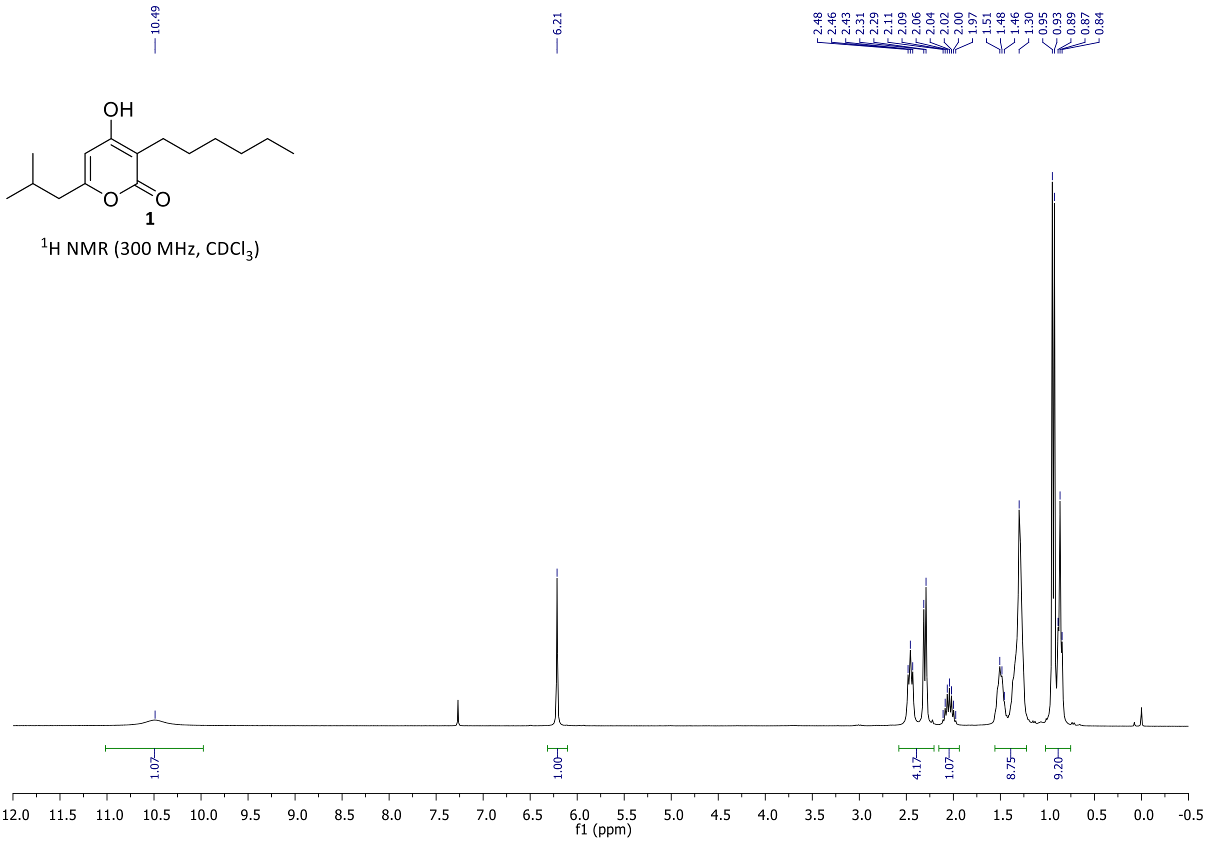

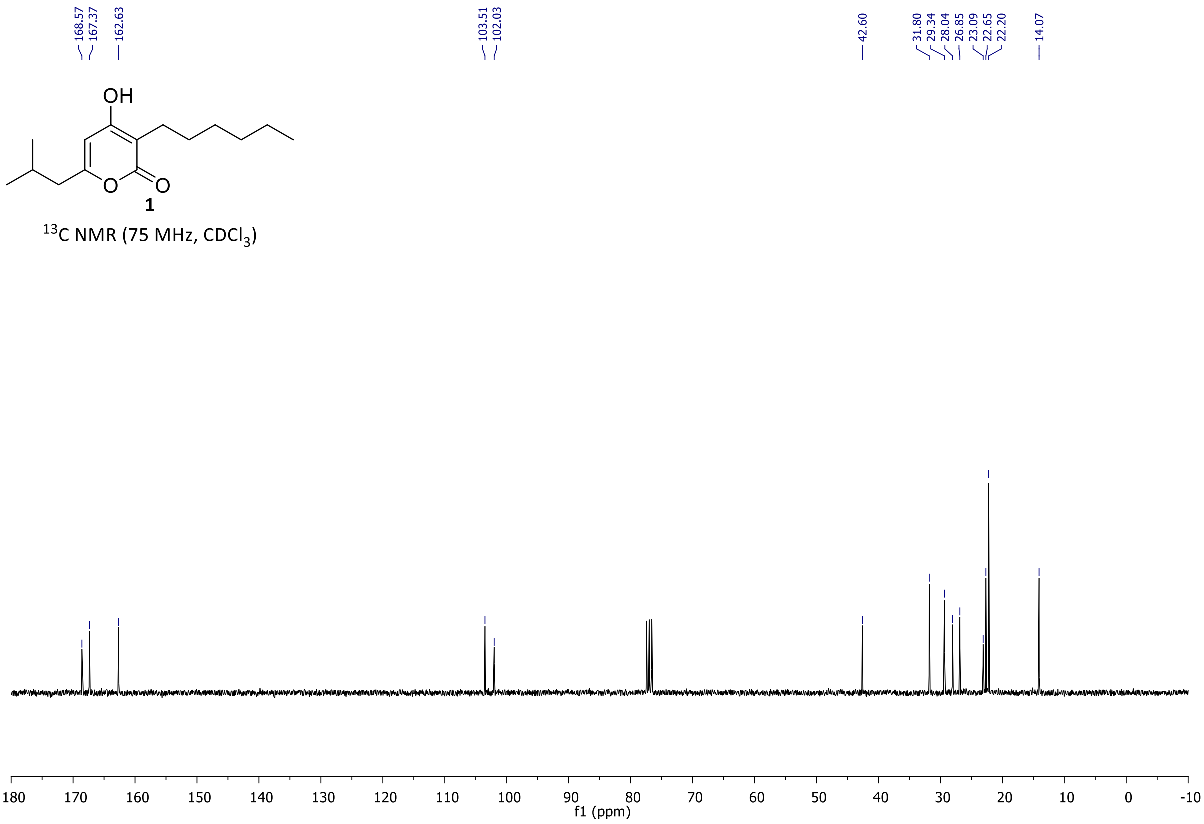

Figure S4: *^{1}H NMR (top) and ^{13}C NMR (bottom) spectra for compound **1**.*

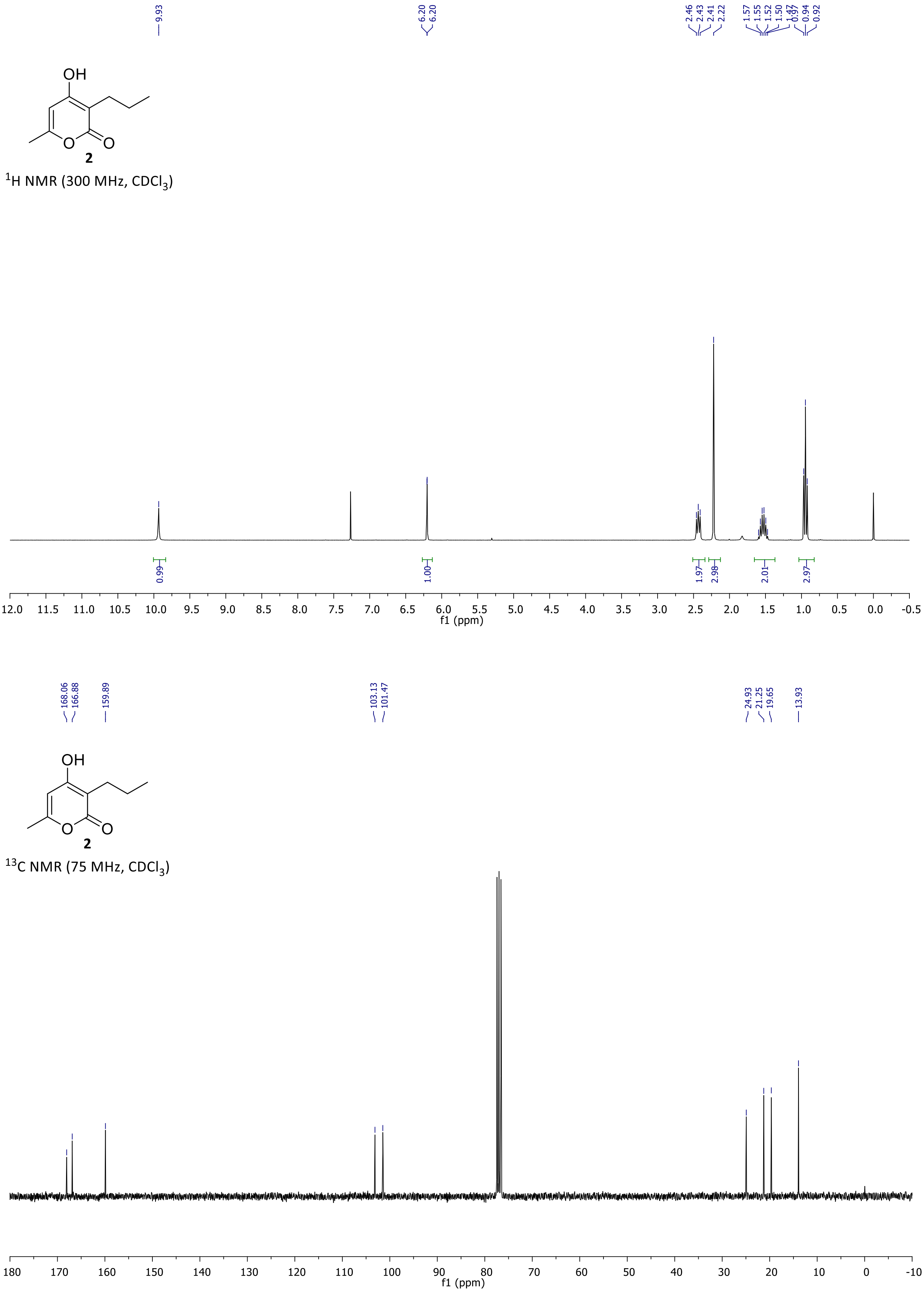

Figure S5: *^{1}H NMR (top) and ^{13}C NMR (bottom) spectra for compound **2**.*

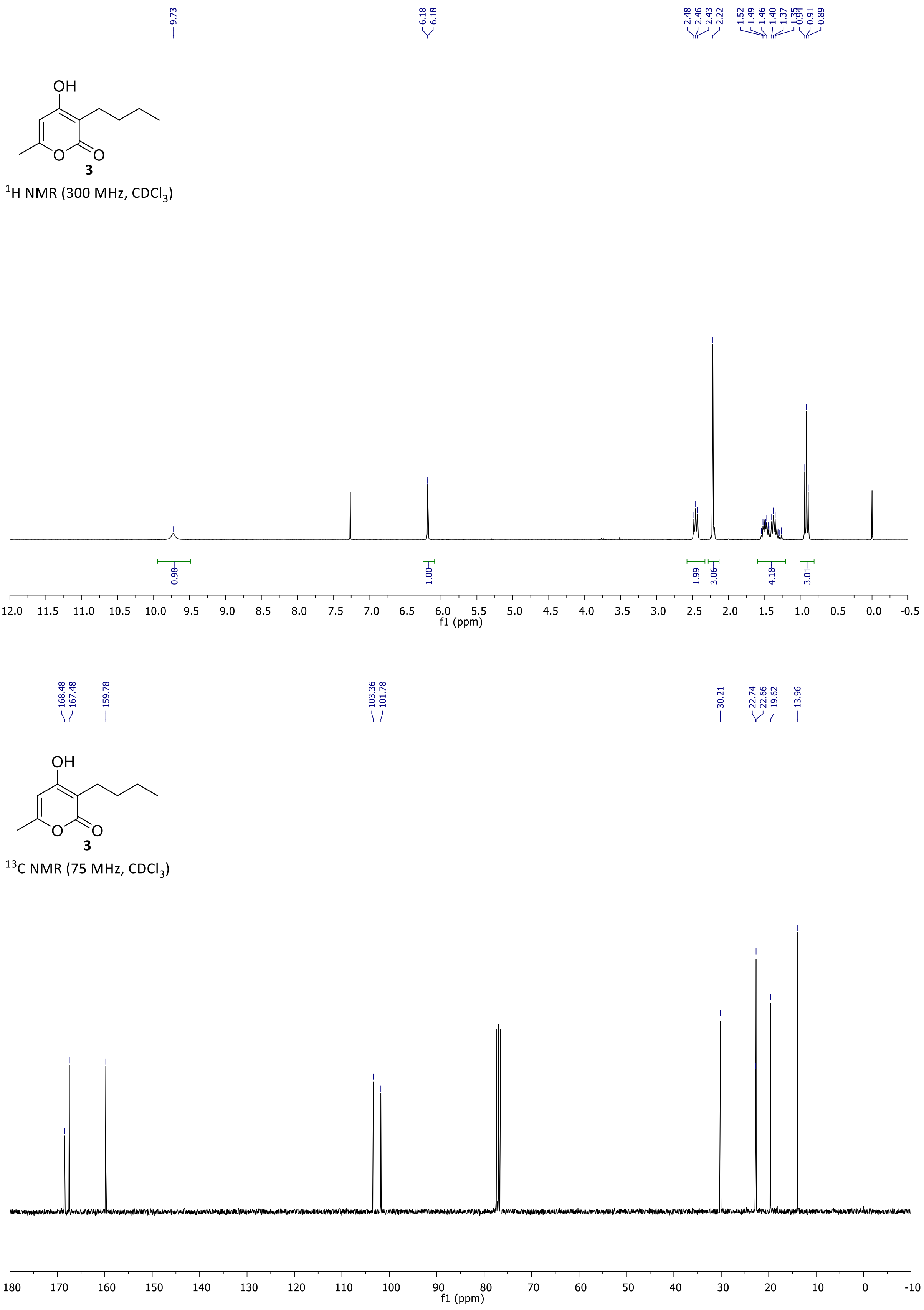

Figure S6: *^{1}H NMR (top) and ^{13}C NMR (bottom) spectra for compound **3**.*

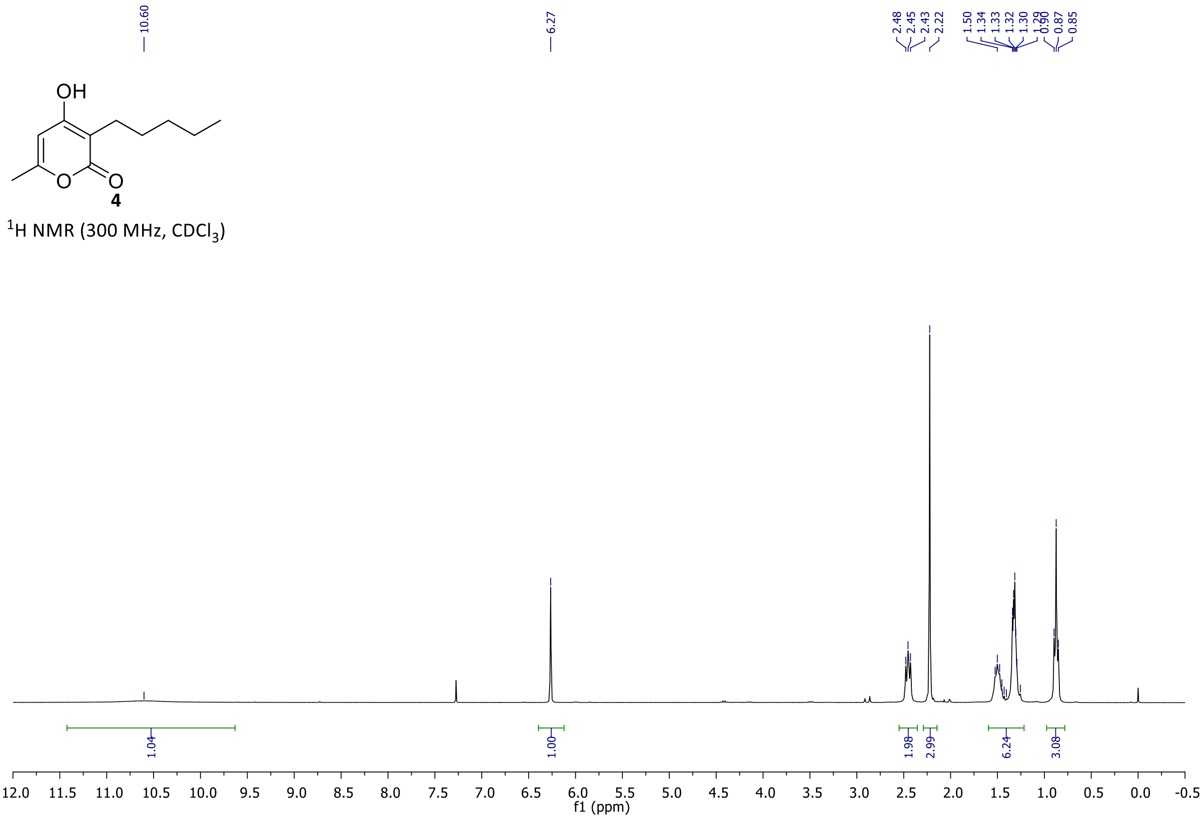

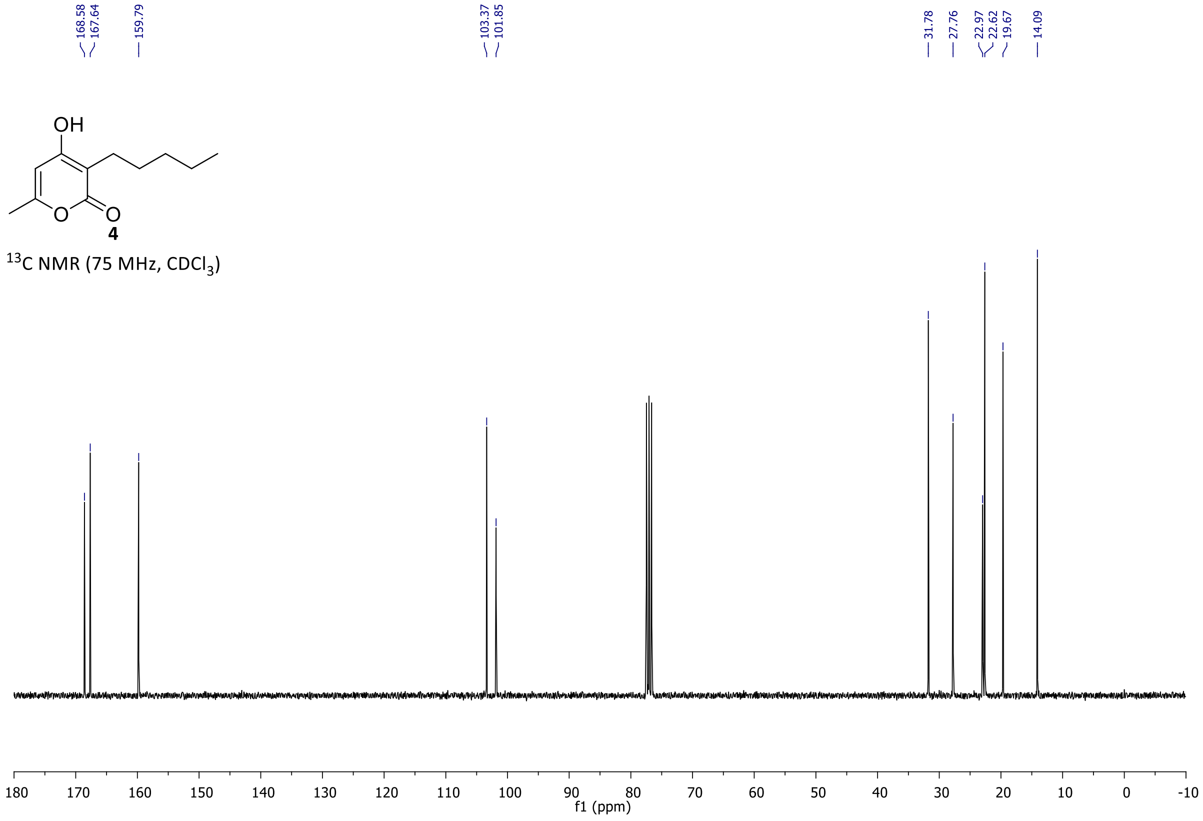

Figure S7: *^{1}H NMR (top) and ^{13}C NMR (bottom) spectra for compound* ***4****.*

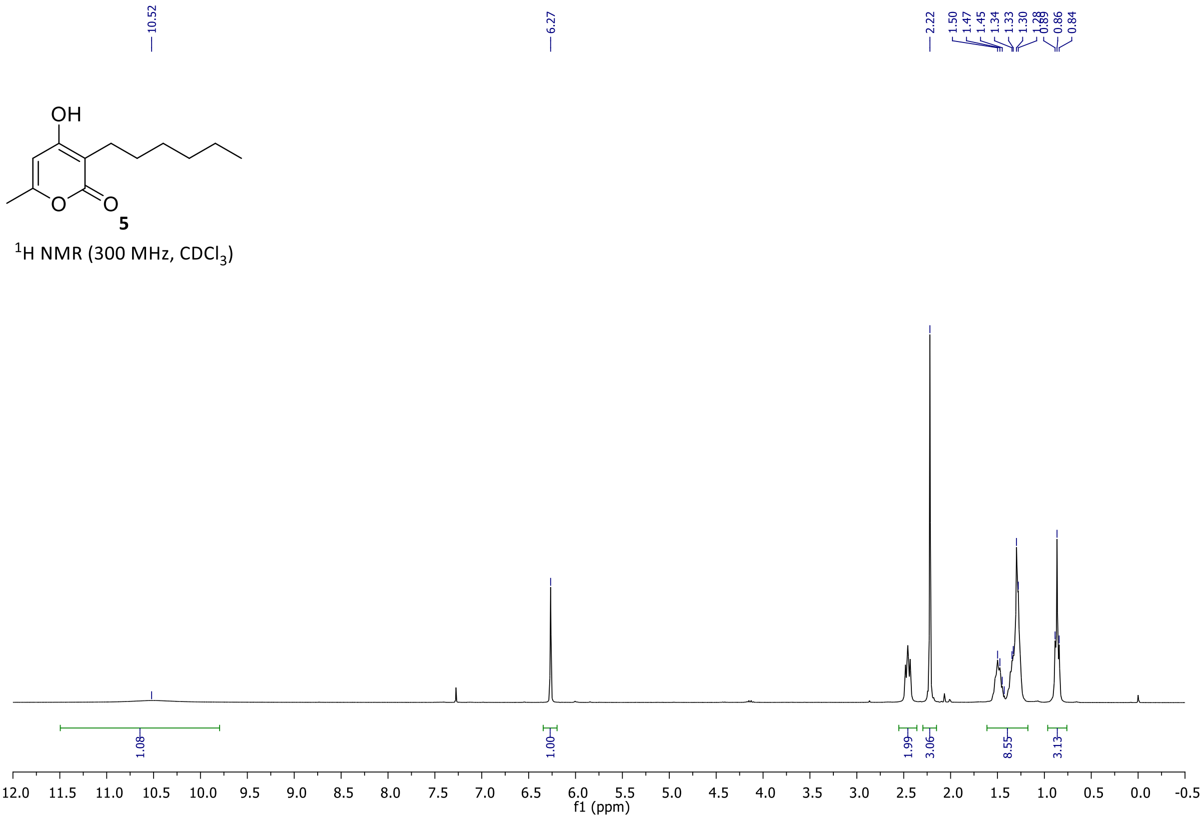

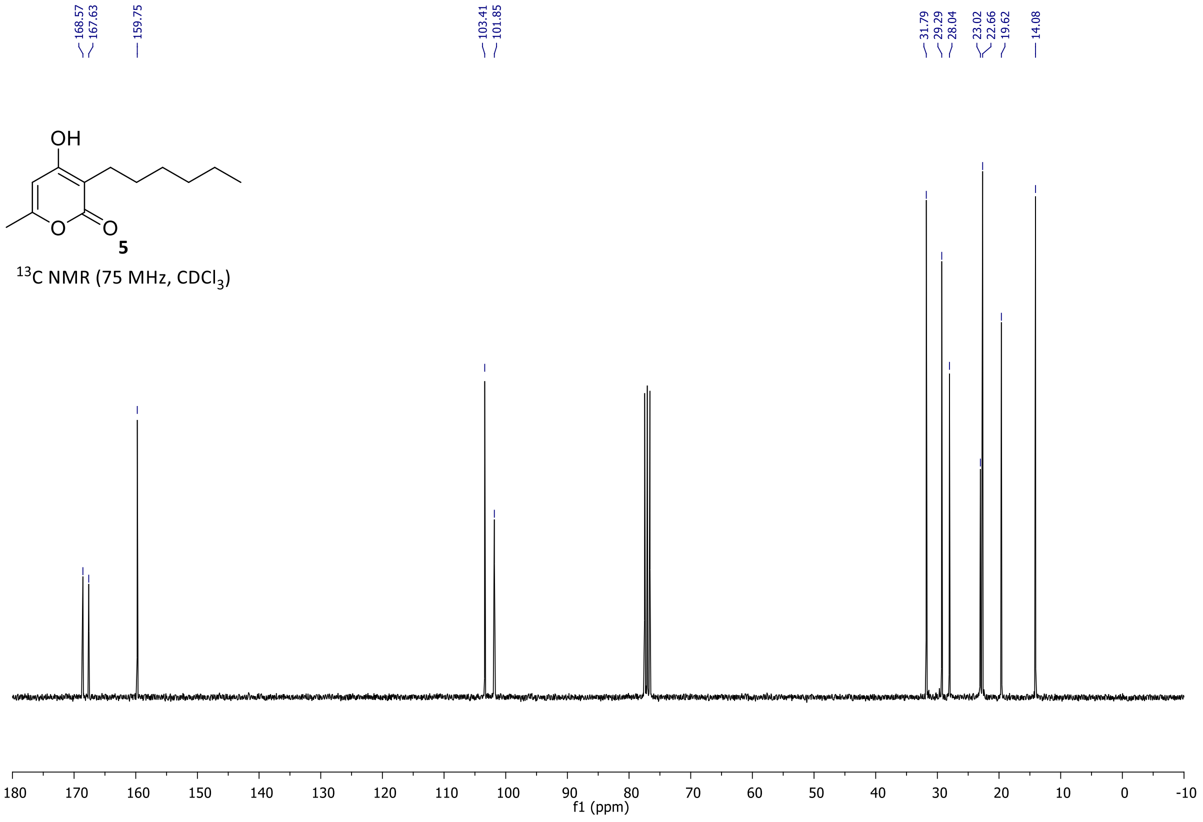

Figure S8: *^{1}H NMR (top) and ^{13}C NMR (bottom) spectra for compound **5**.*

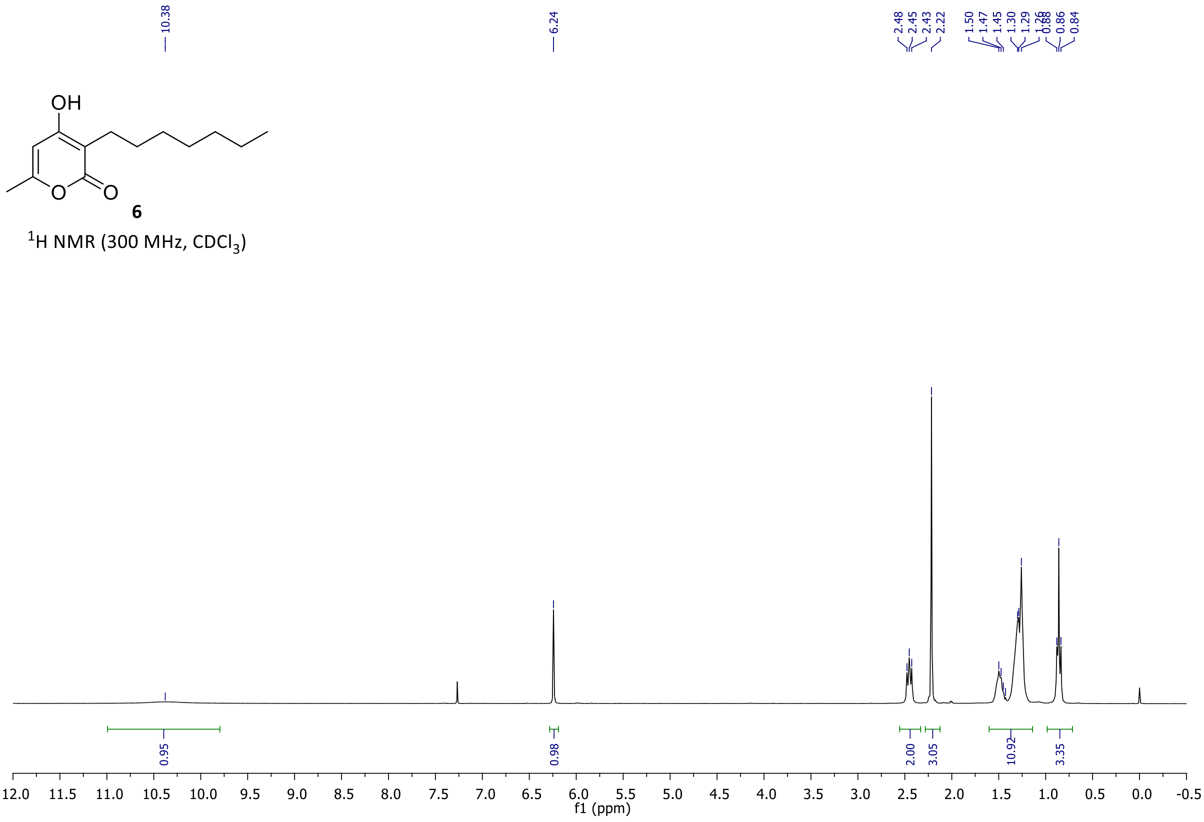

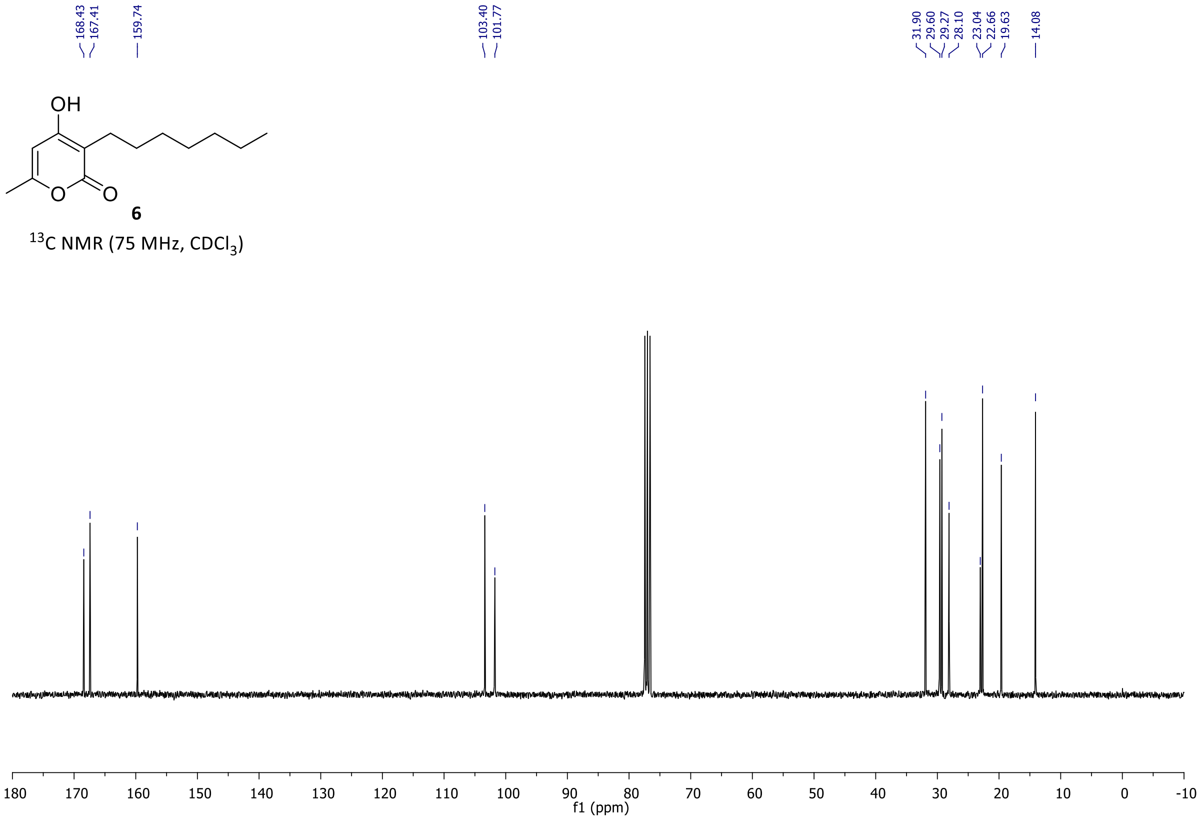

Figure S9: *^{1}H NMR (top) and ^{13}C NMR (bottom) spectra for compound* ***6****.*

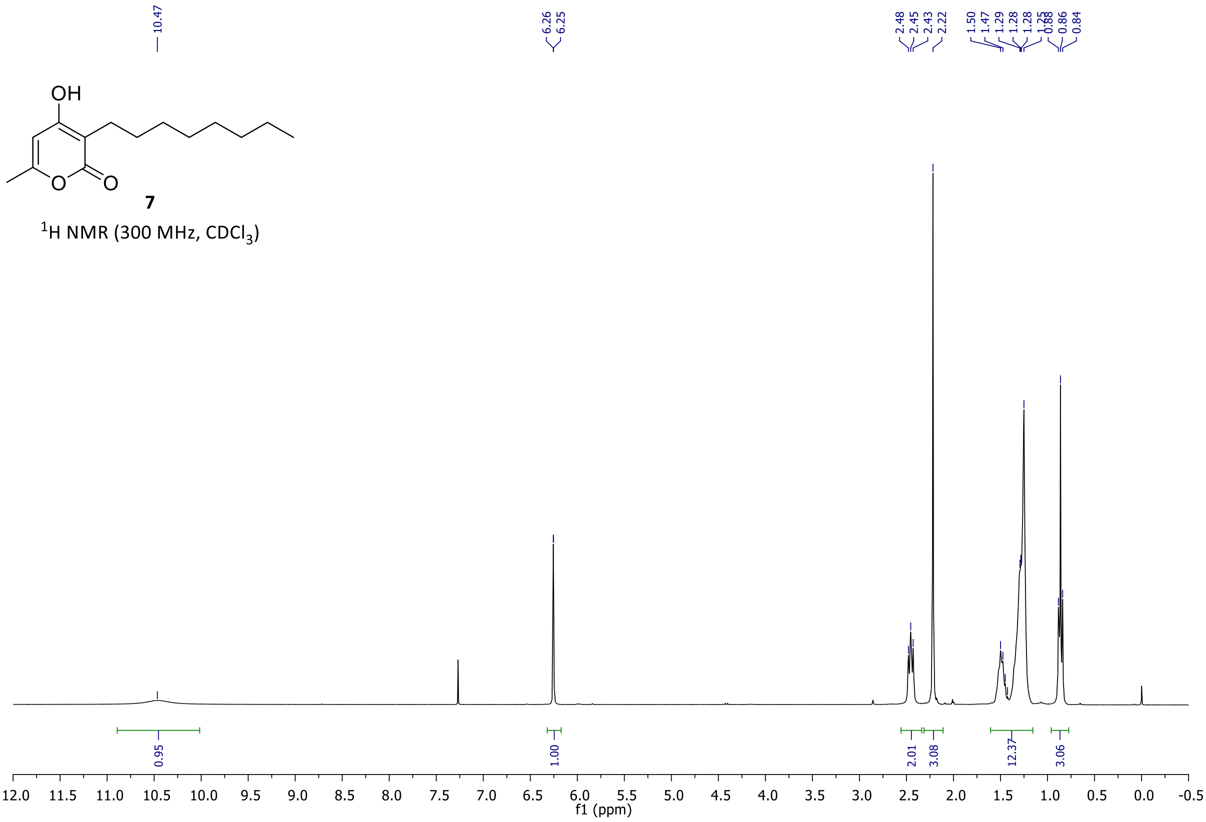

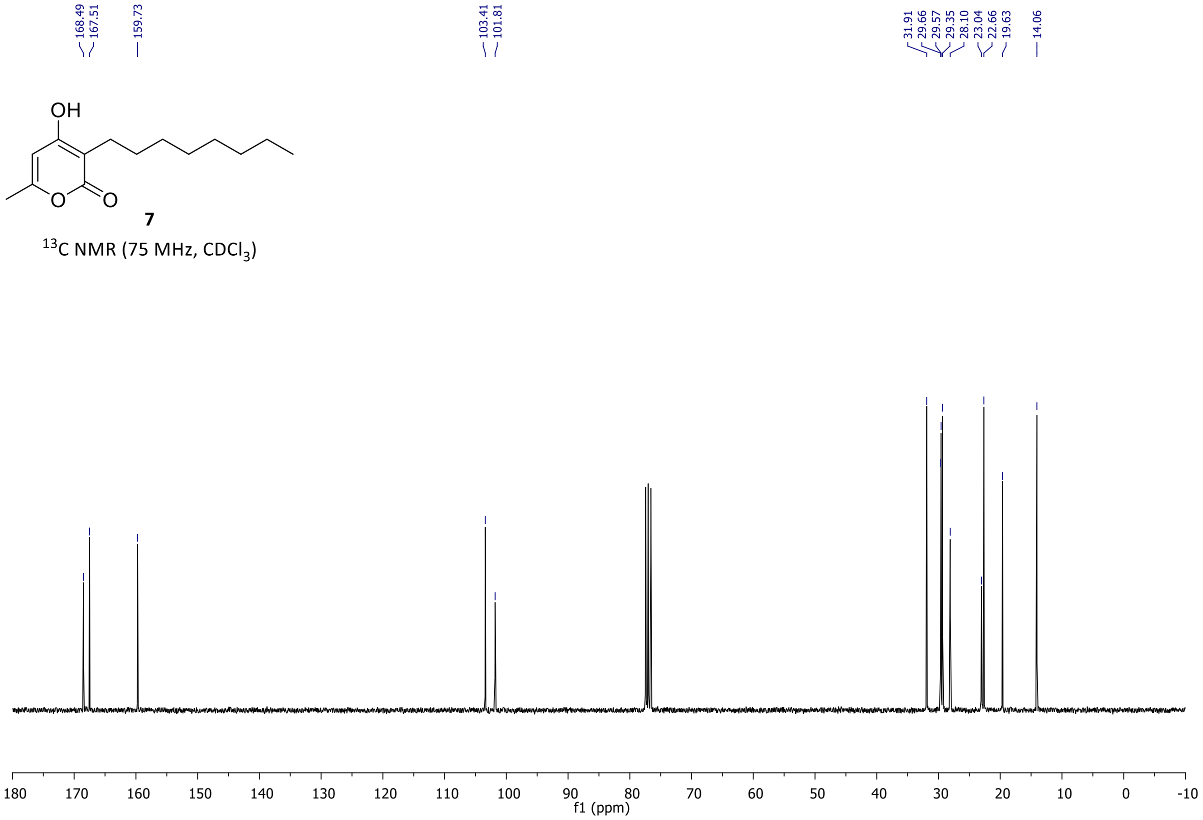

Figure S10: *^{1}H NMR (top) and ^{13}C NMR (bottom) spectra for compound **7**.*

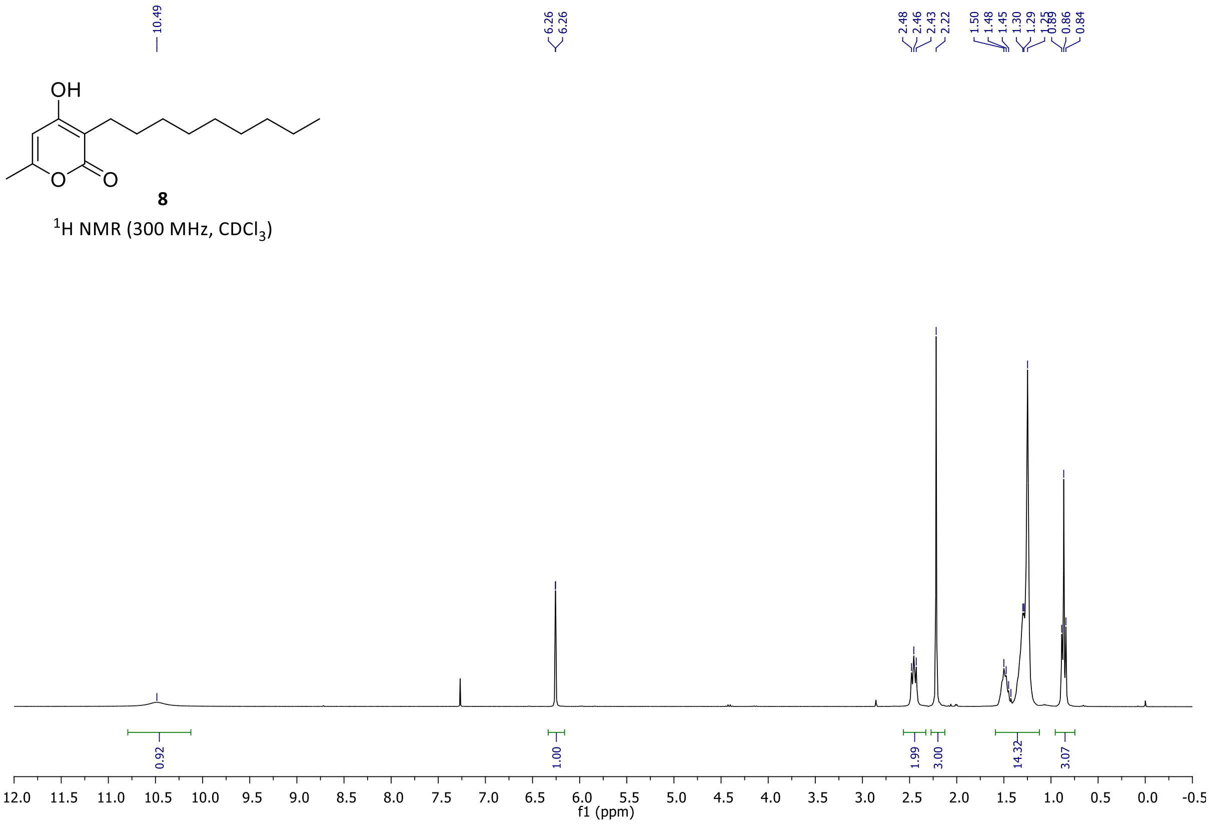

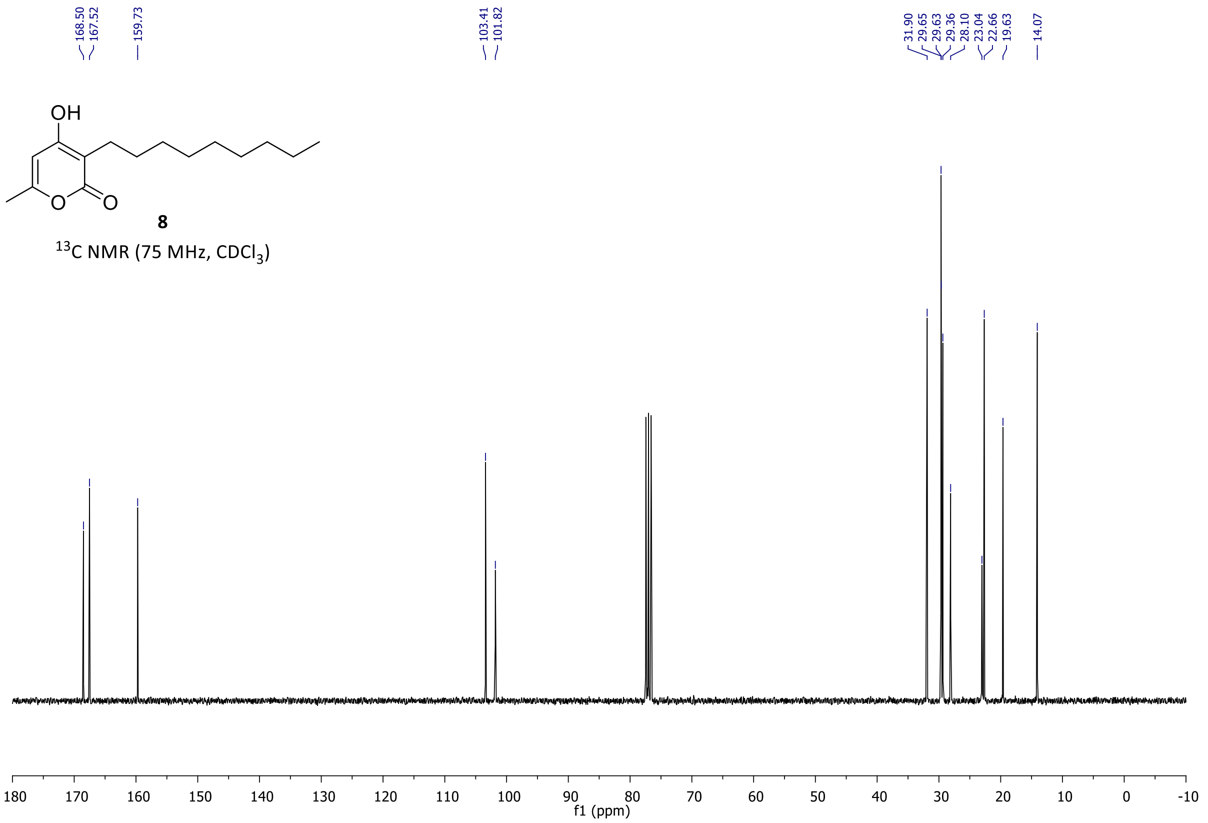

***Figure S11:** ^{1}H NMR (top) and ^{13}C NMR (bottom) spectra for compound **8**.*

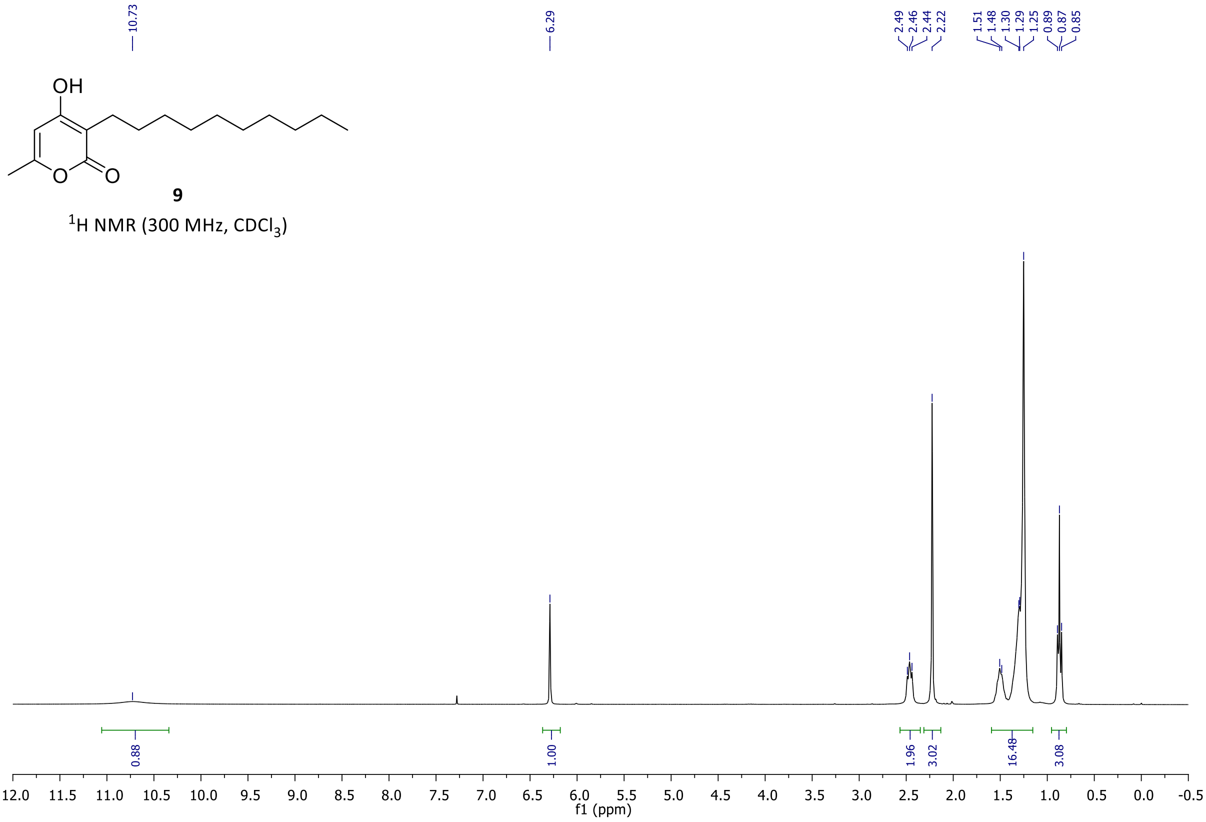

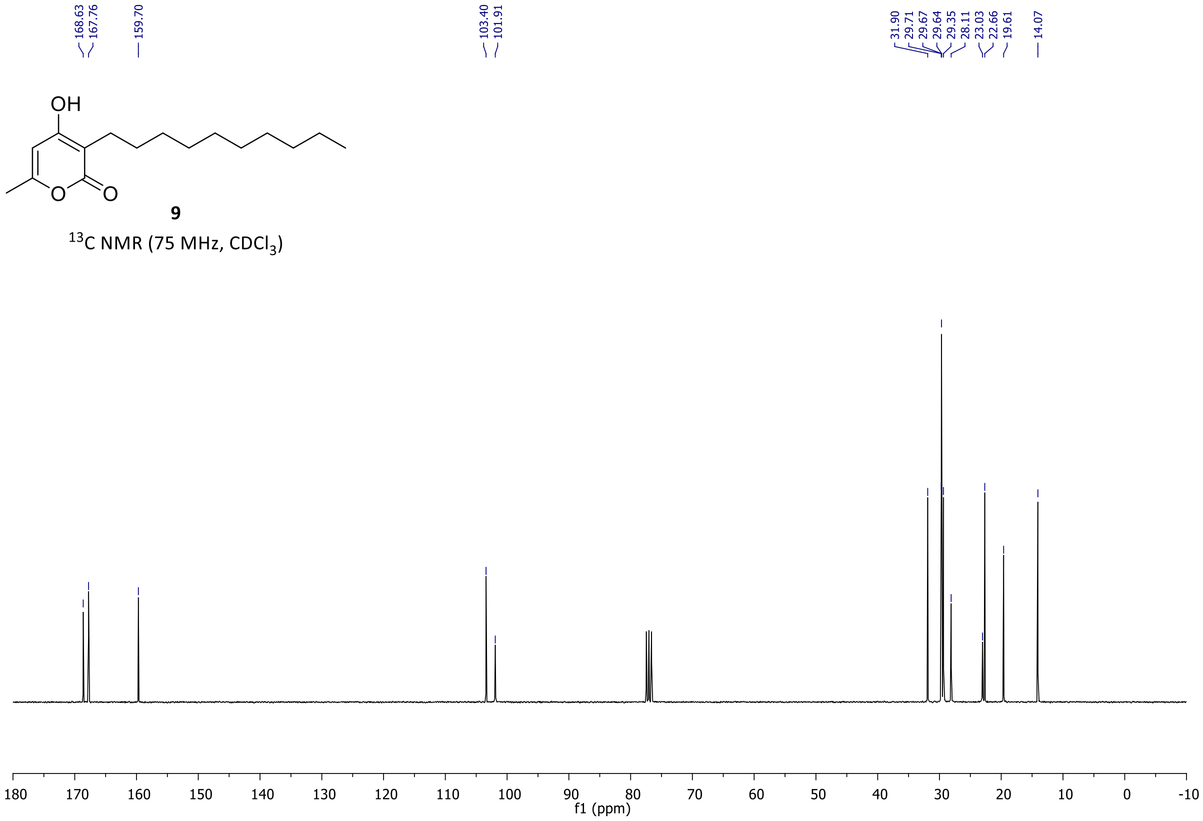

Figure S12: *^{1}H NMR (top) and ^{13}C NMR (bottom) spectra for compound **9**.*

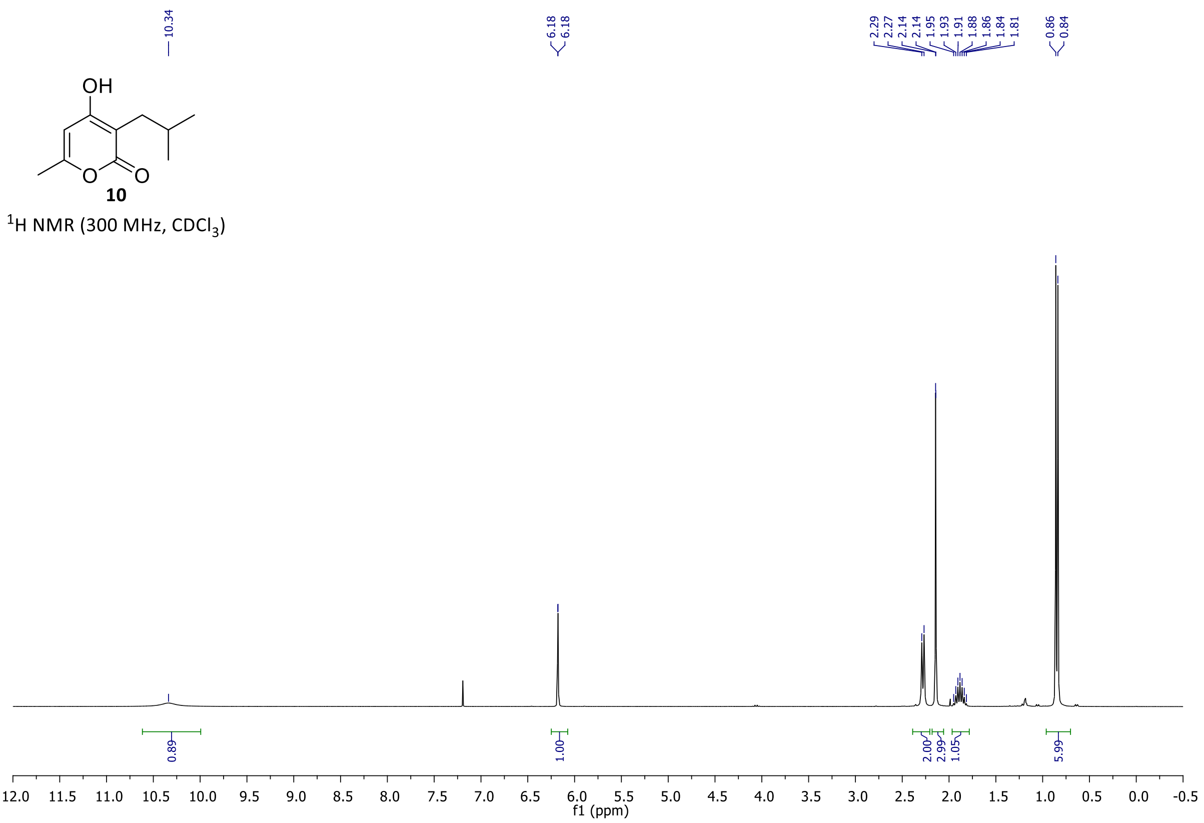

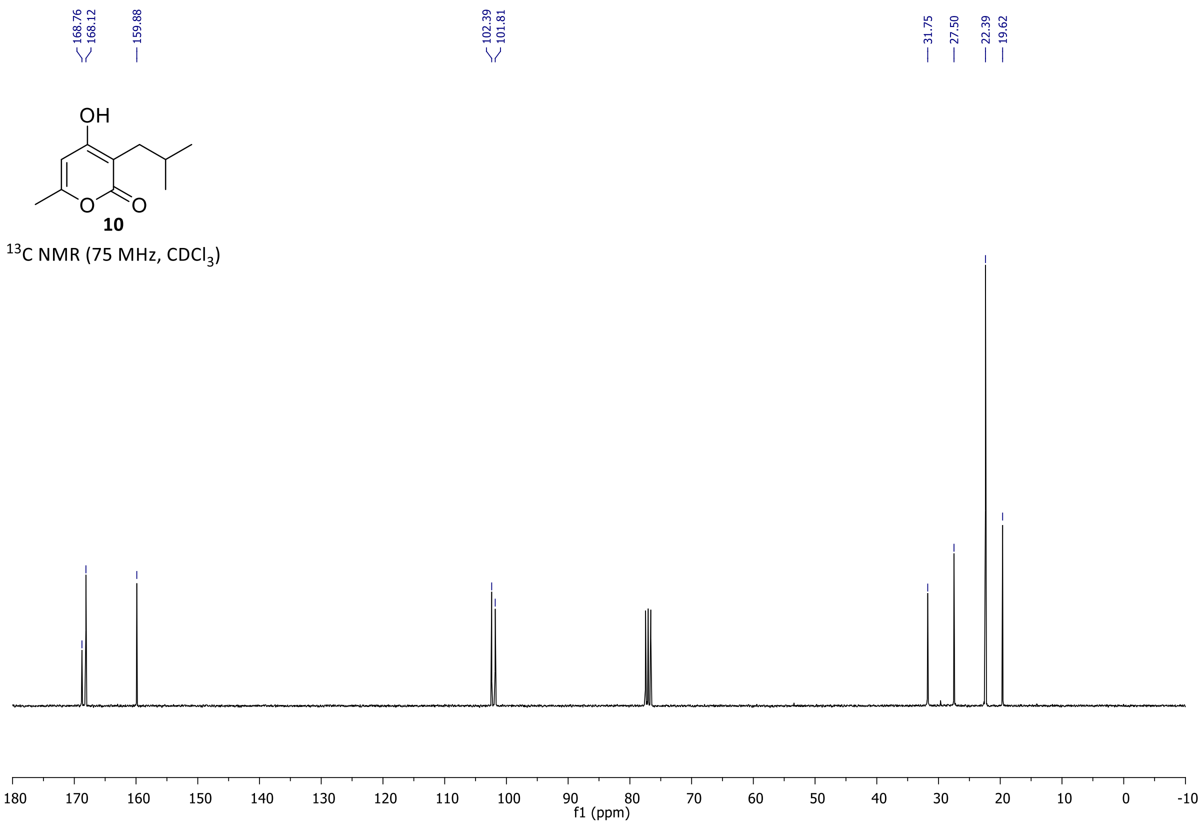

Figure S13: *^{1}H NMR (top) and ^{13}C NMR (bottom) spectra for compound* ***10****.*

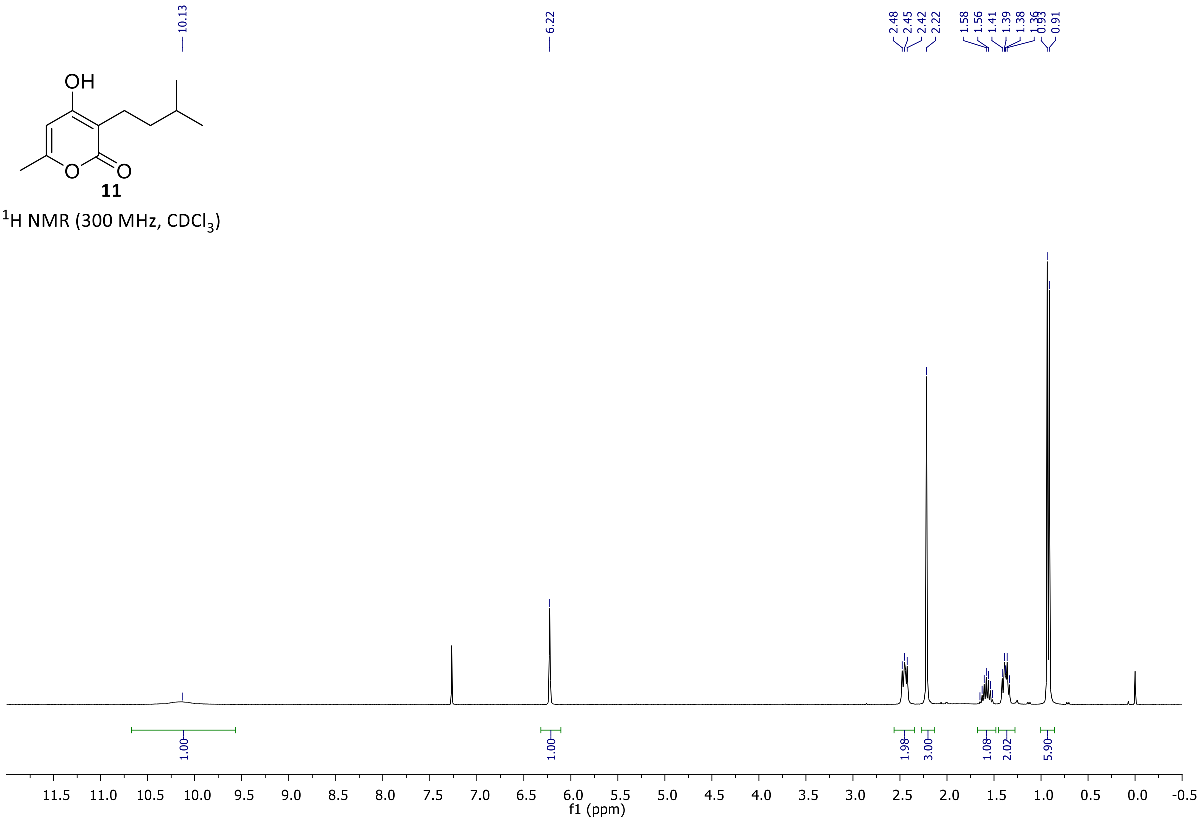

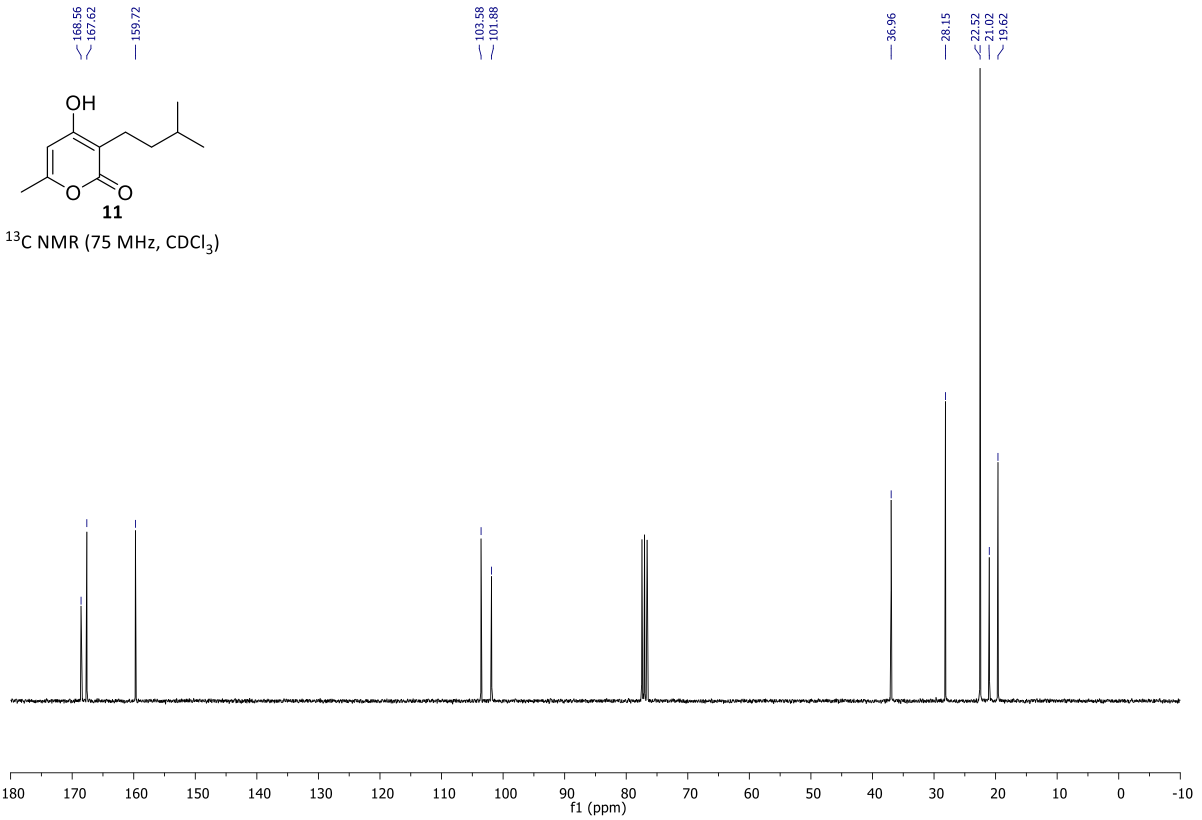

Figure S14: *^{1}H NMR (top) and ^{13}C NMR (bottom) spectra for compound* ***11****.*

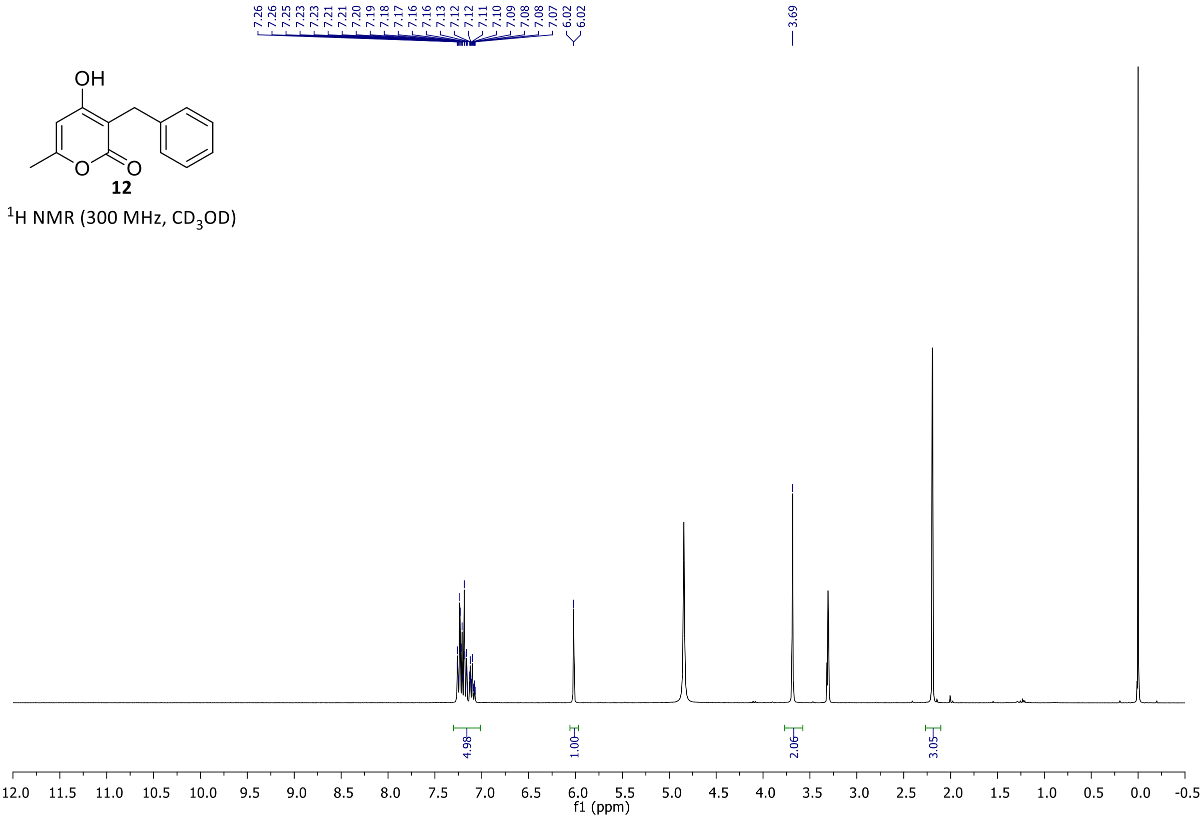

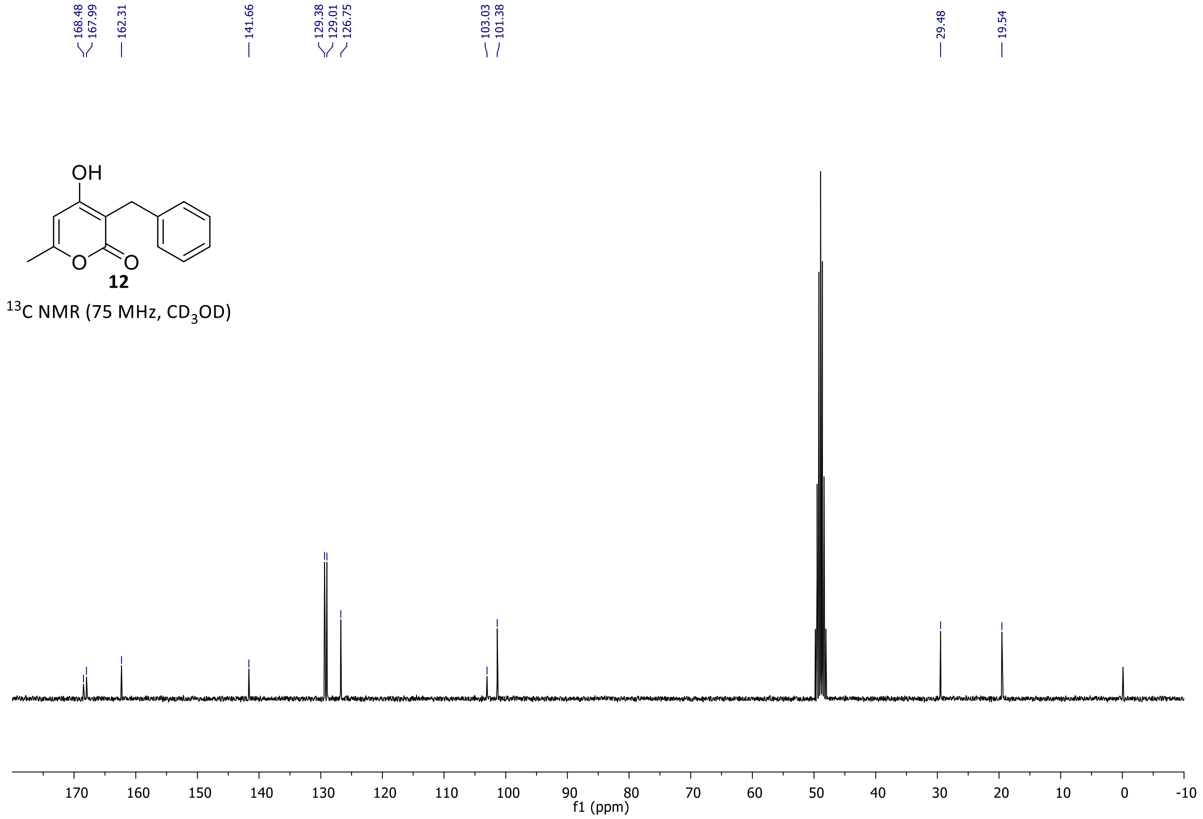

Figure S15: *^{1}H NMR (top) and ^{13}C NMR (bottom) spectra for compound* ***12****.*

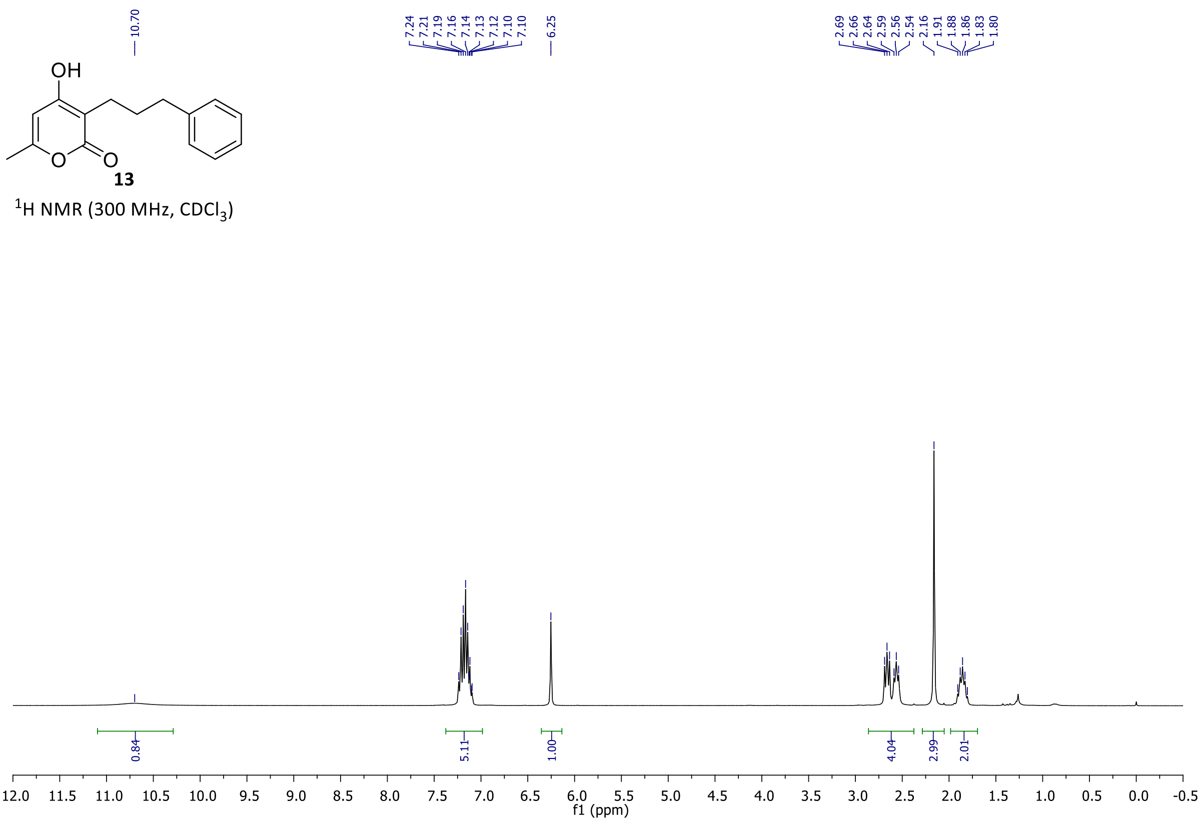

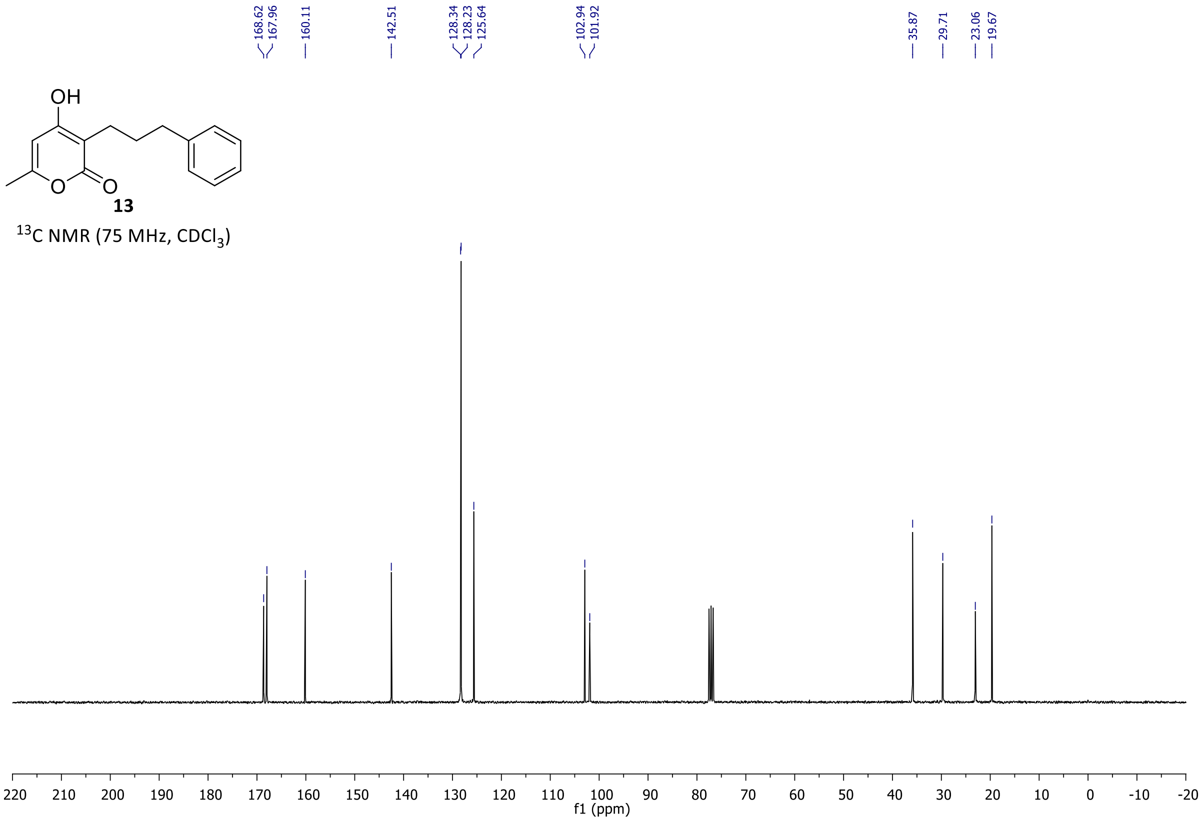

Figure S16: *^{1}H NMR (top) and ^{13}C NMR (bottom) spectra for compound **13**.*

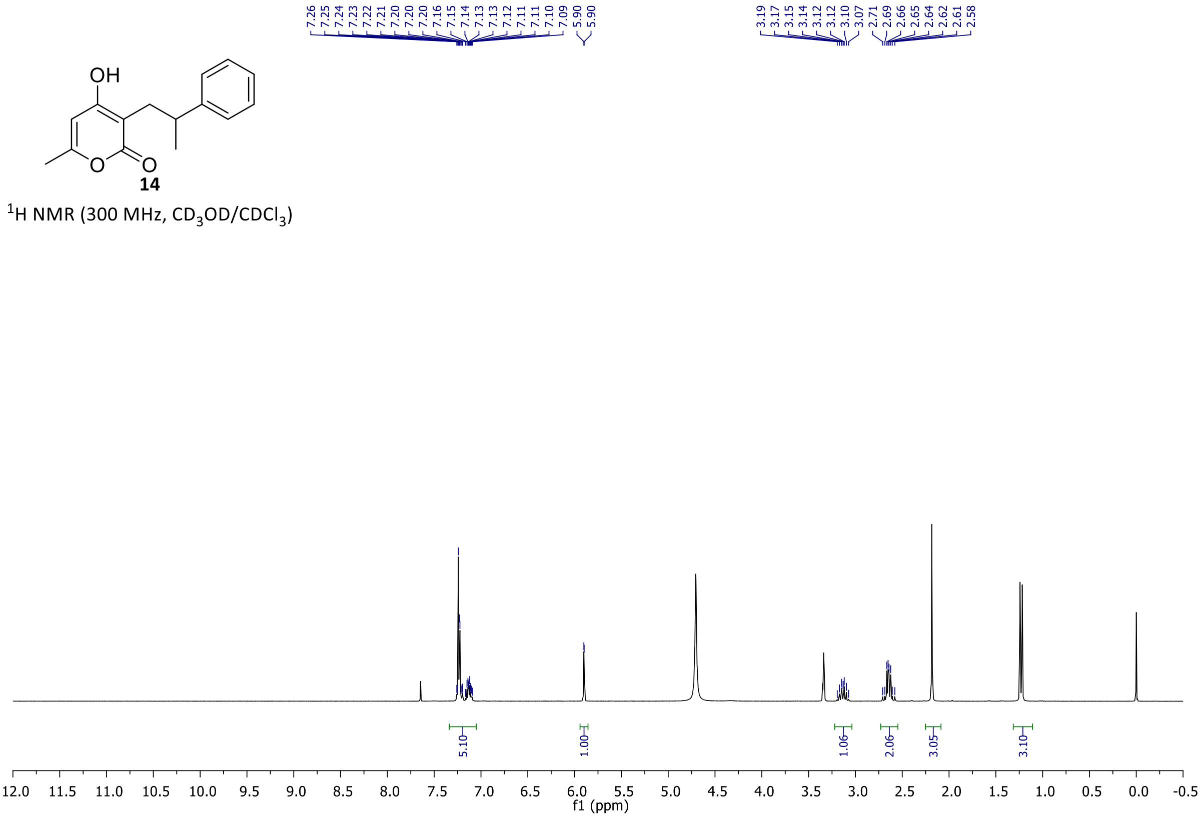

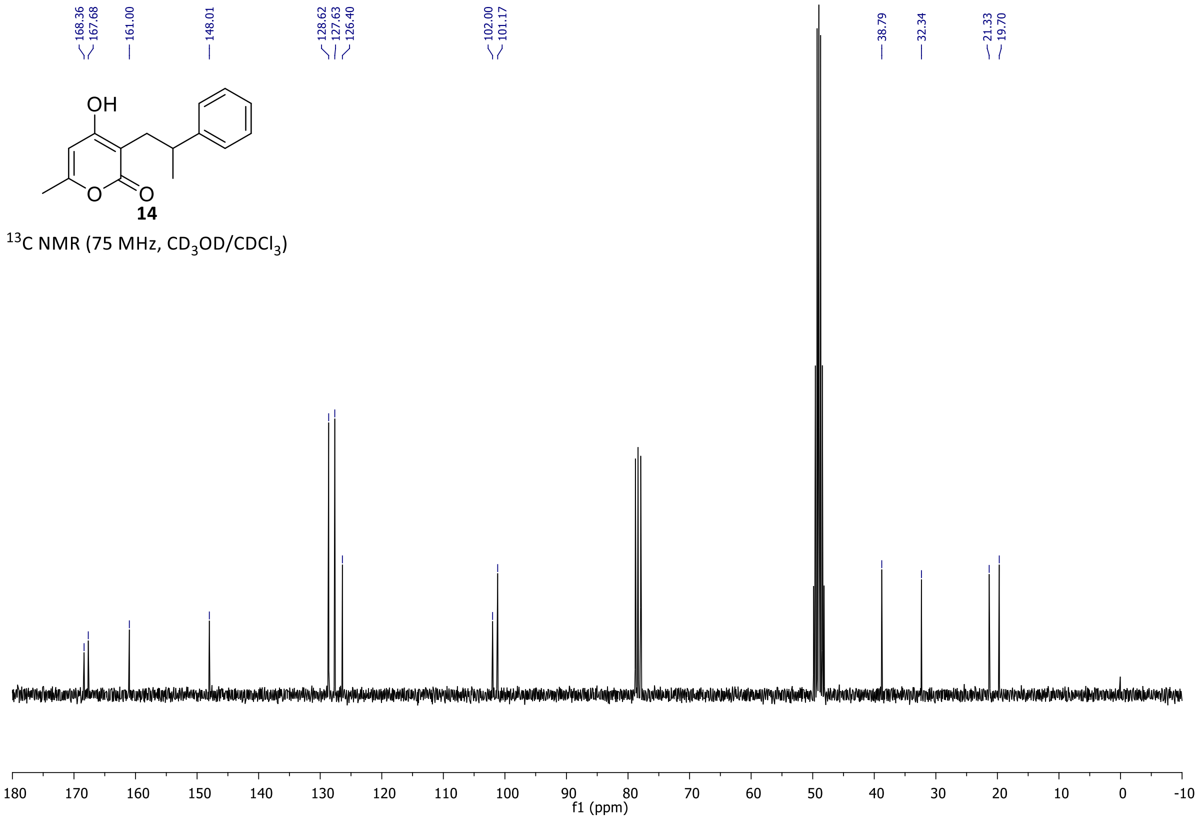

Figure S17: ^{1}H *NMR (top) and* ^{13}C *NMR (bottom) spectra for compound* ***14***.

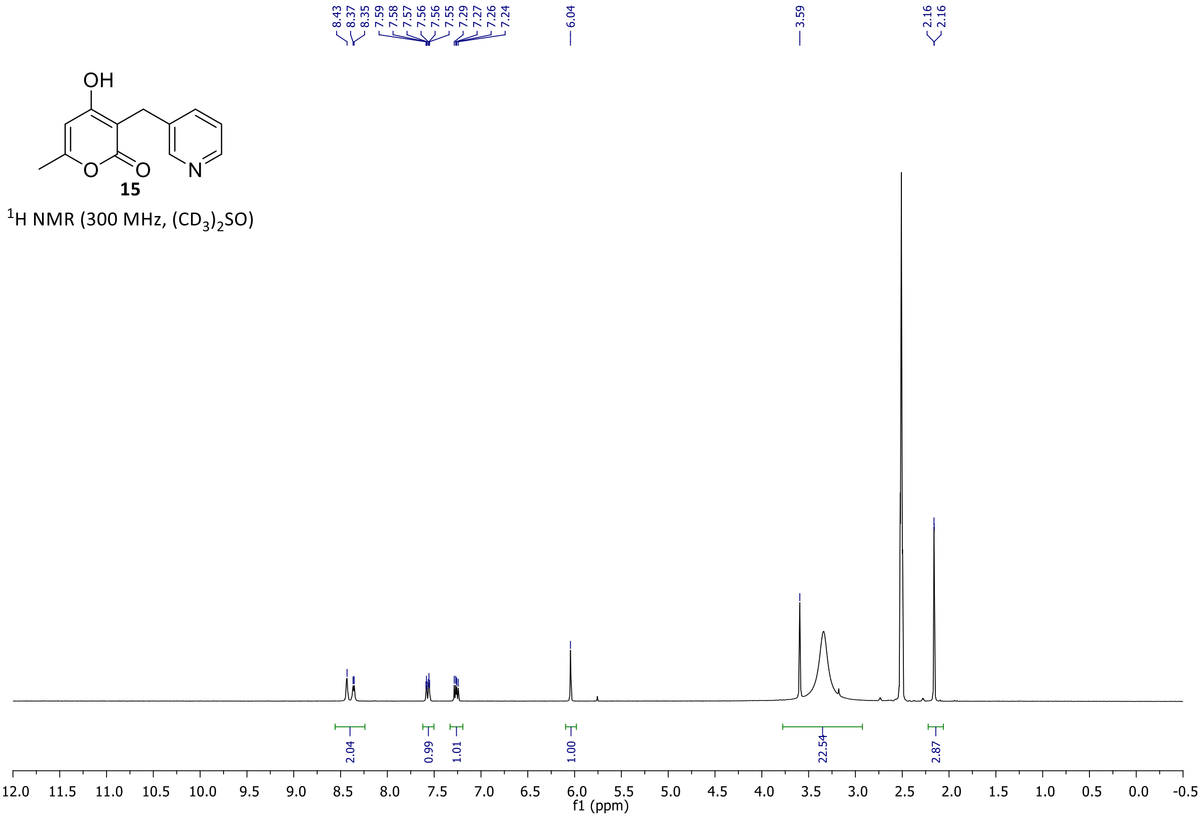

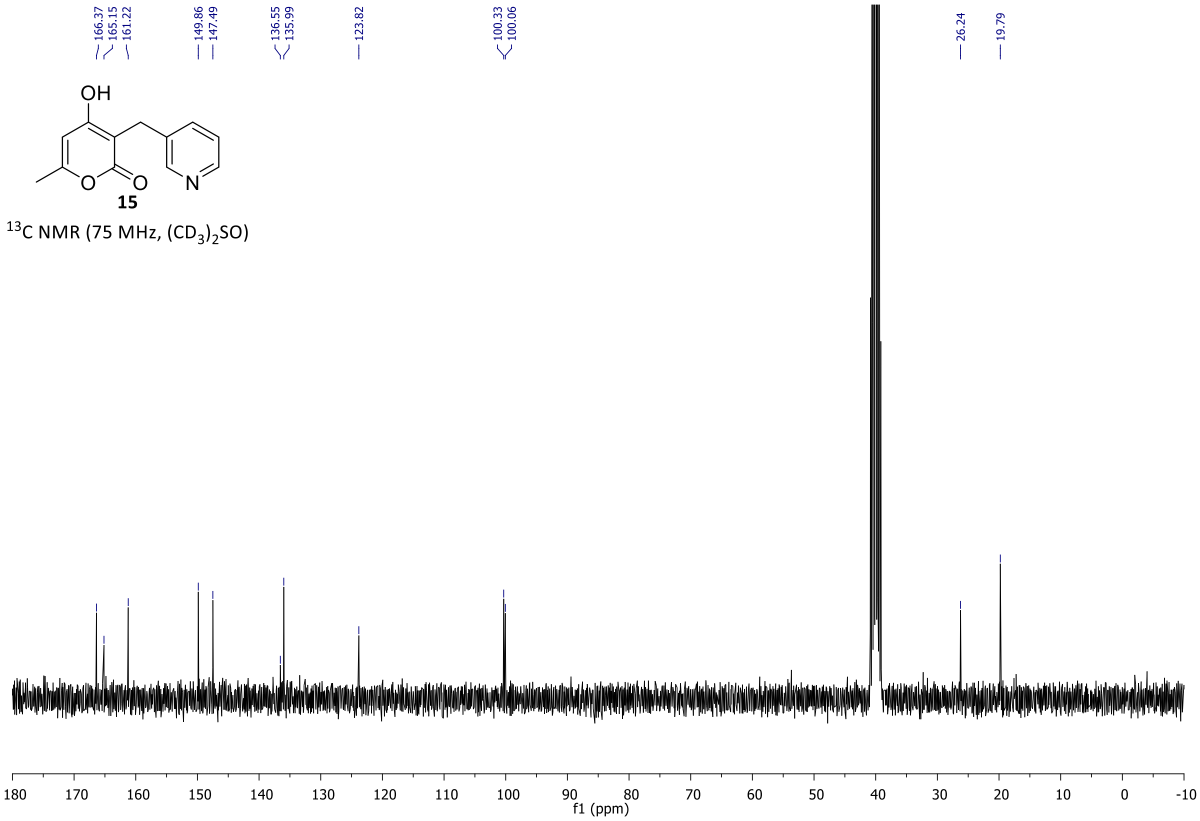

Figure S18: *^{1}H NMR (top) and ^{13}C NMR (bottom) spectra for compound* ***15****.*

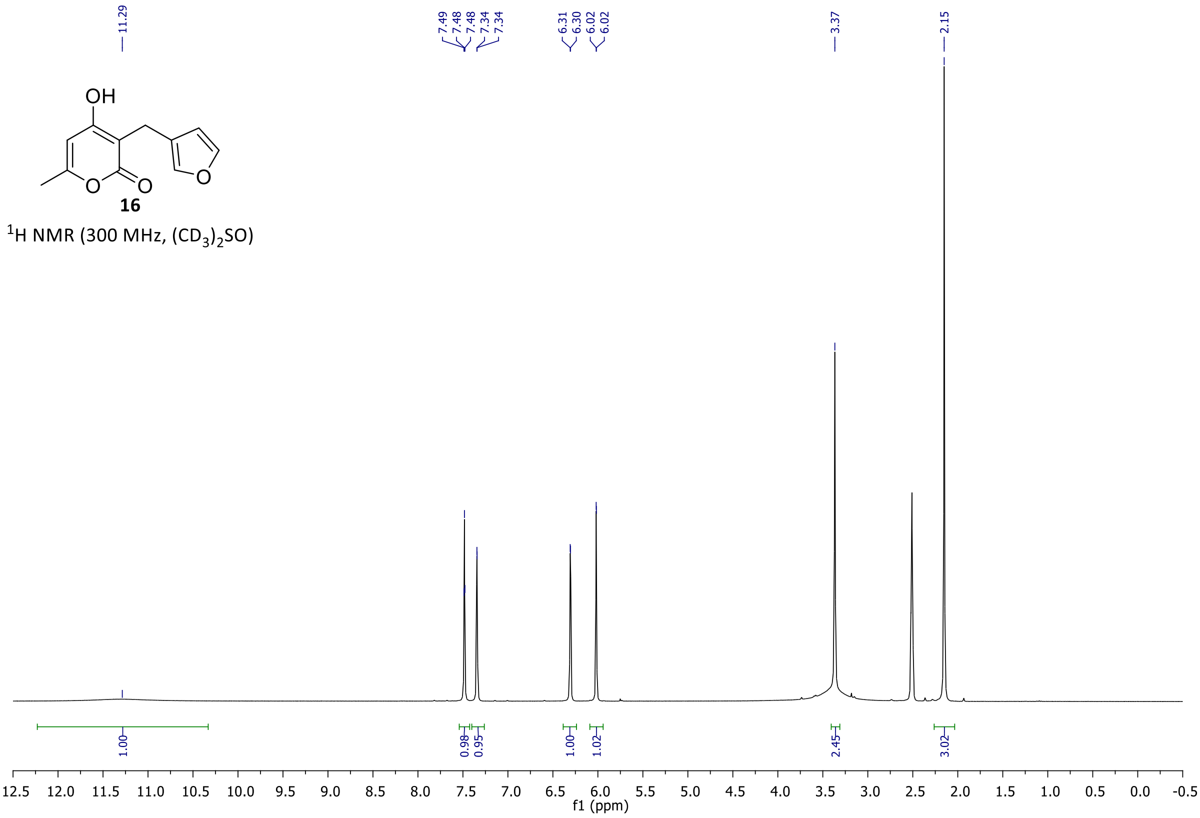

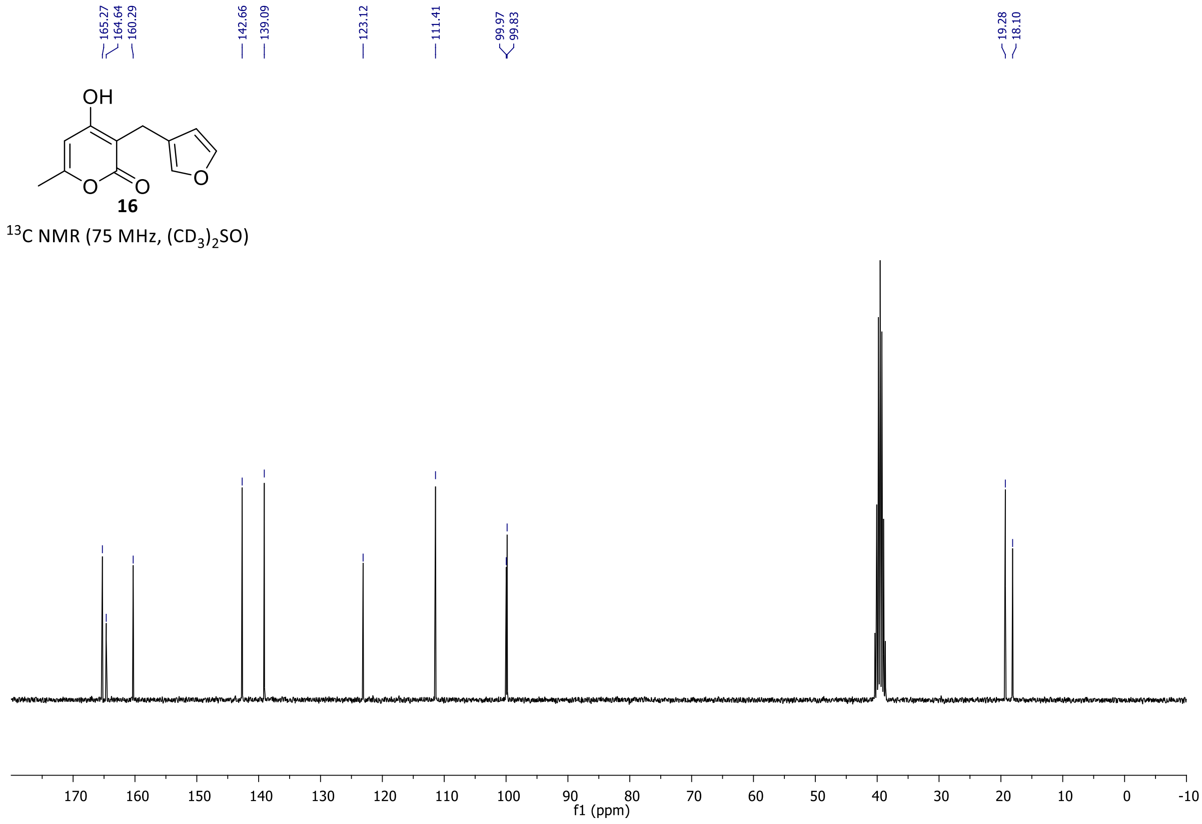

Figure S19: *^{1}H NMR (top) and ^{13}C NMR (bottom) spectra for compound* ***16****.*

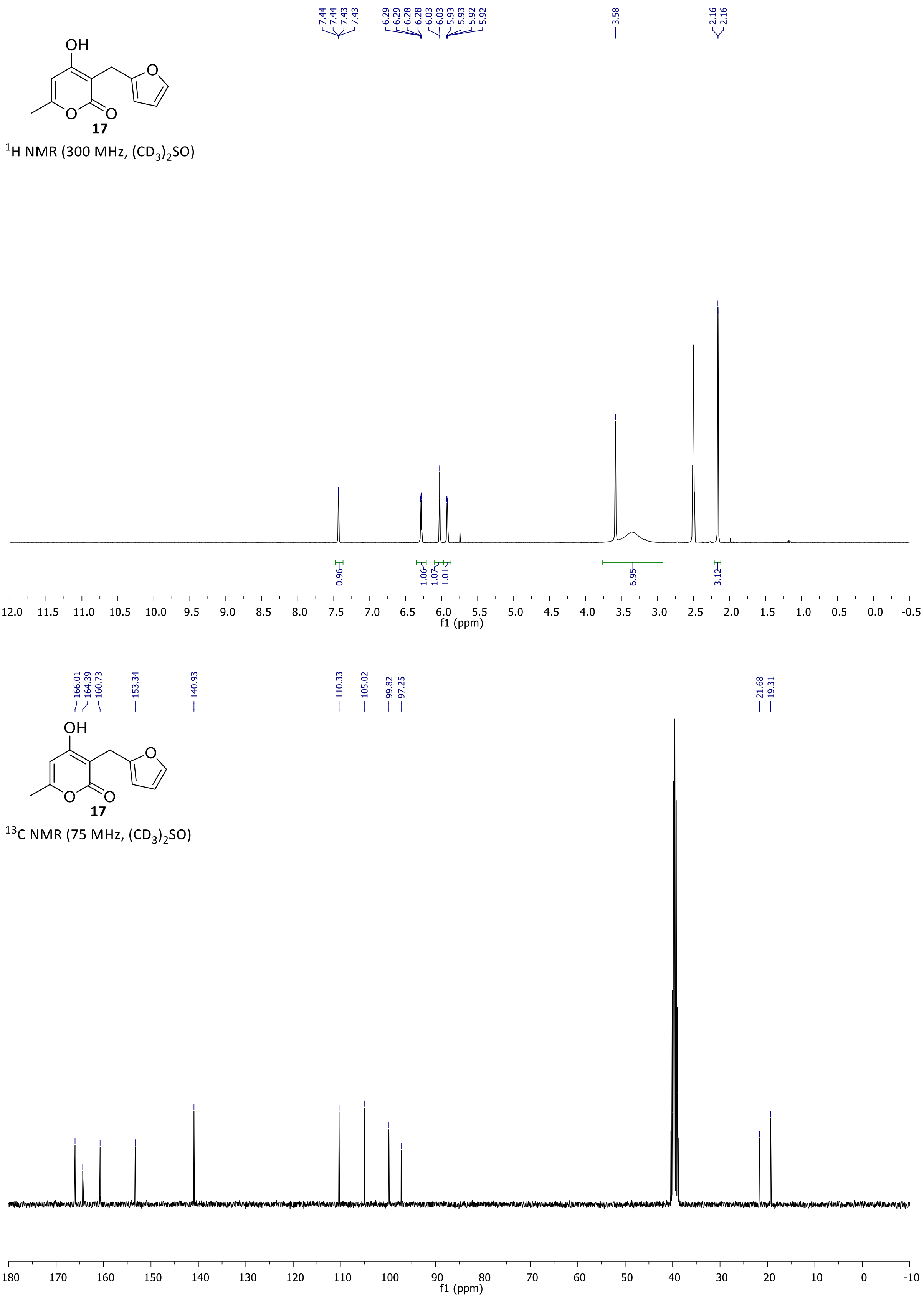

Figure S20: *^{1}H NMR (top) and ^{13}C NMR (bottom) spectra for compound* ***17****.*

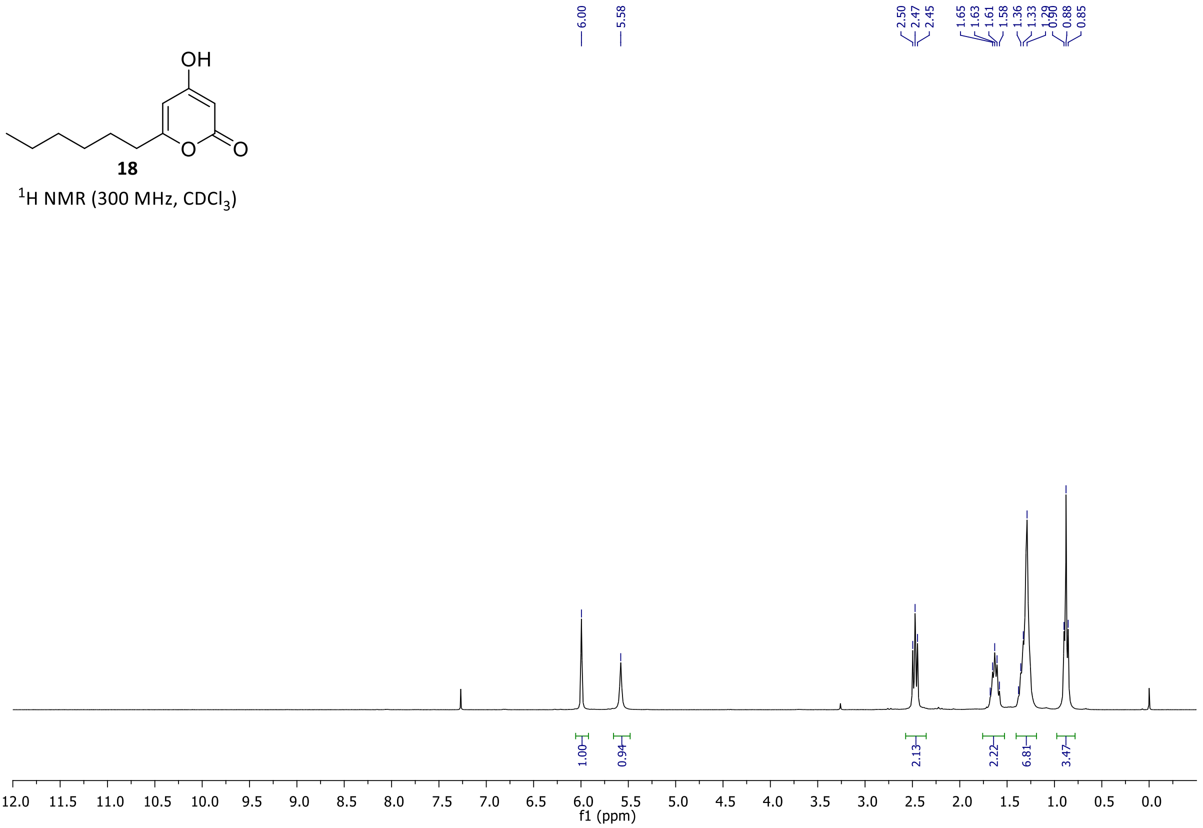

18

^{1}H NMR (300 MHz, $CDCl_3$)

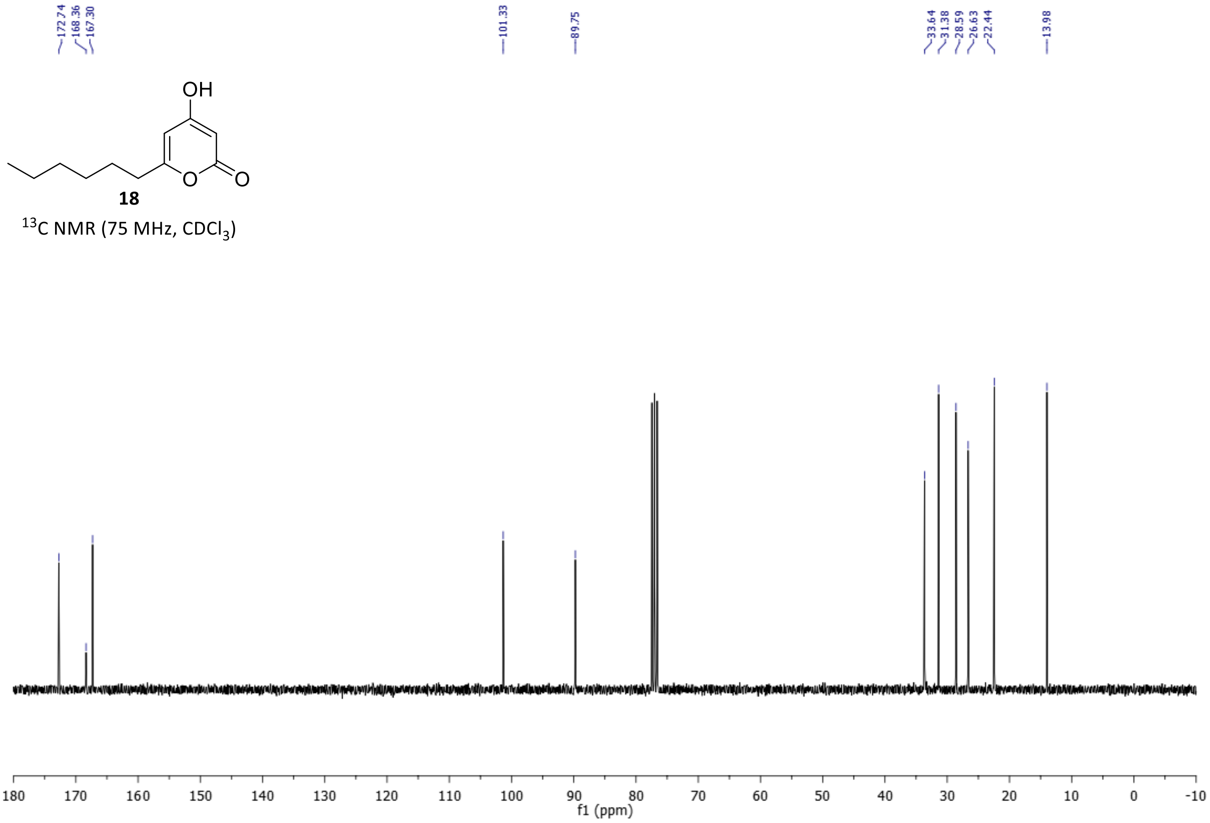

18

^{13}C NMR (75 MHz, $CDCl_3$)

Figure S21: *^{1}H NMR (top) and ^{13}C NMR (bottom) spectra for compound* ***18****.*

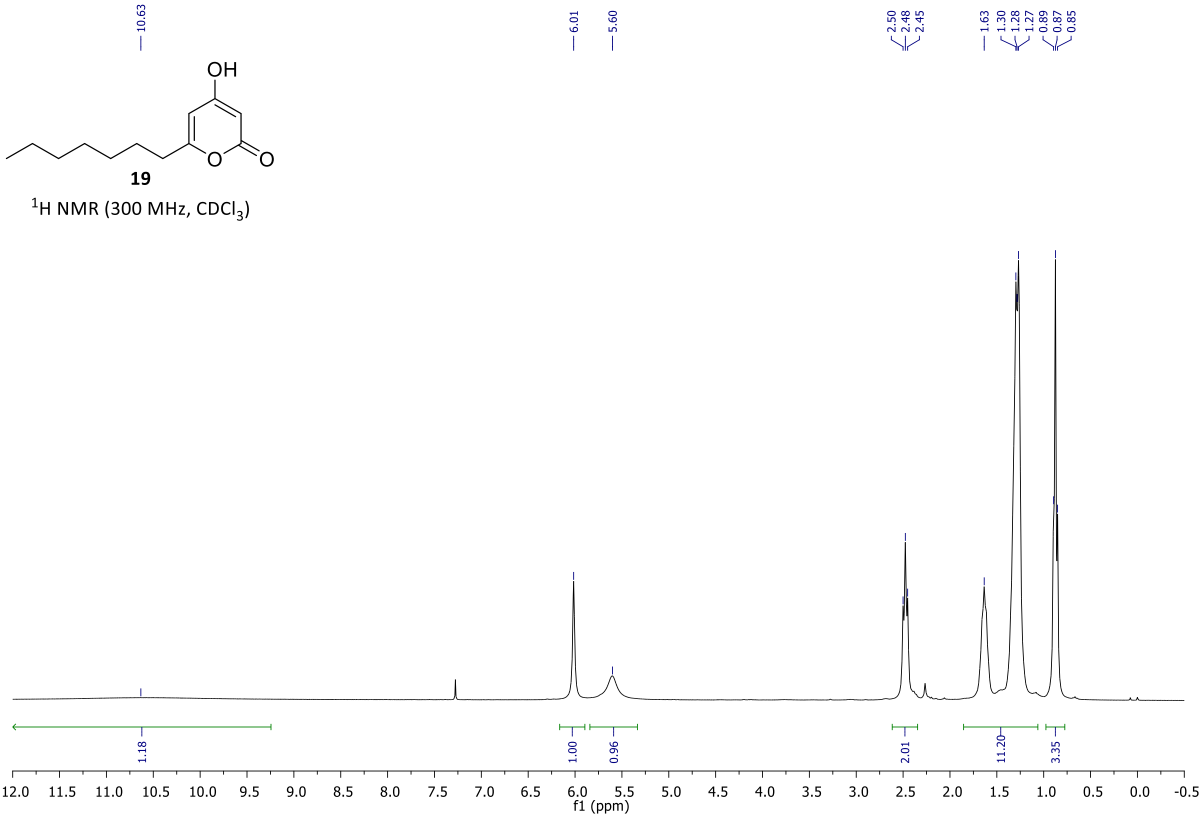

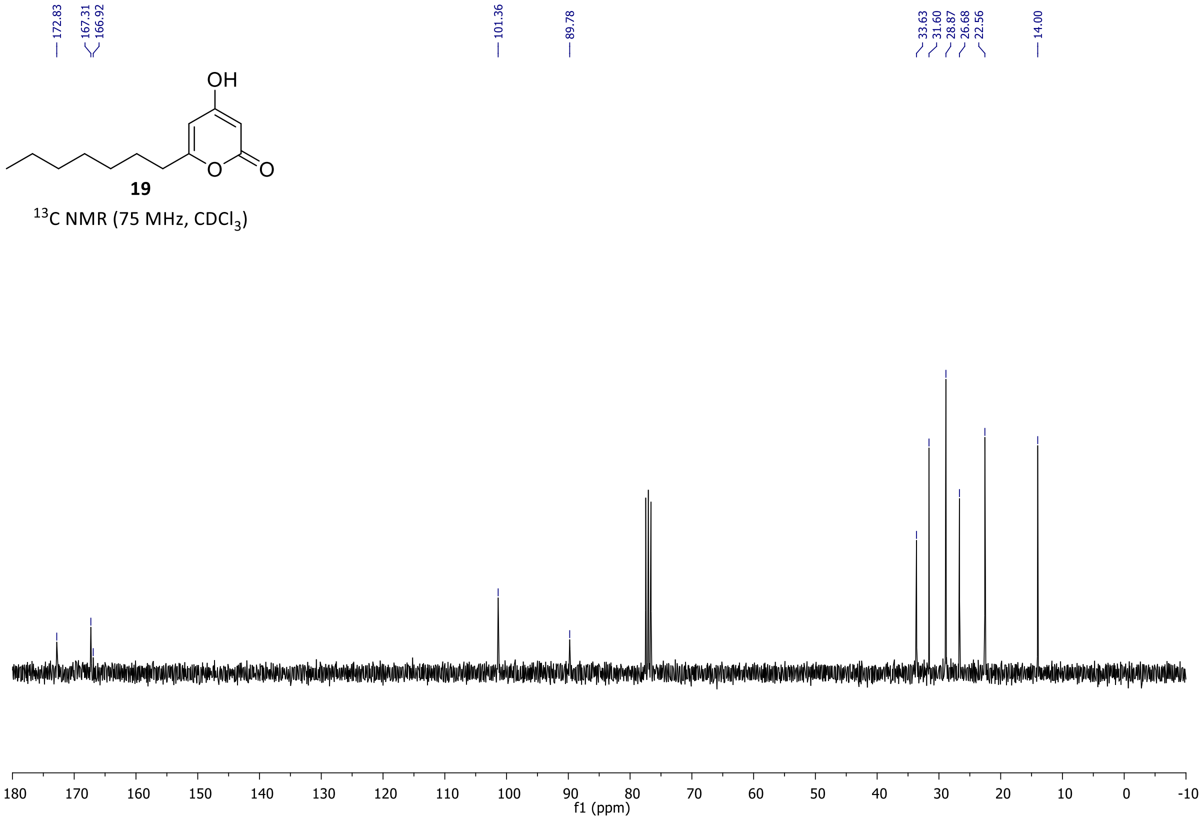

Figure S22: *^{1}H NMR (top) and ^{13}C NMR (bottom) spectra for compound **19**.*

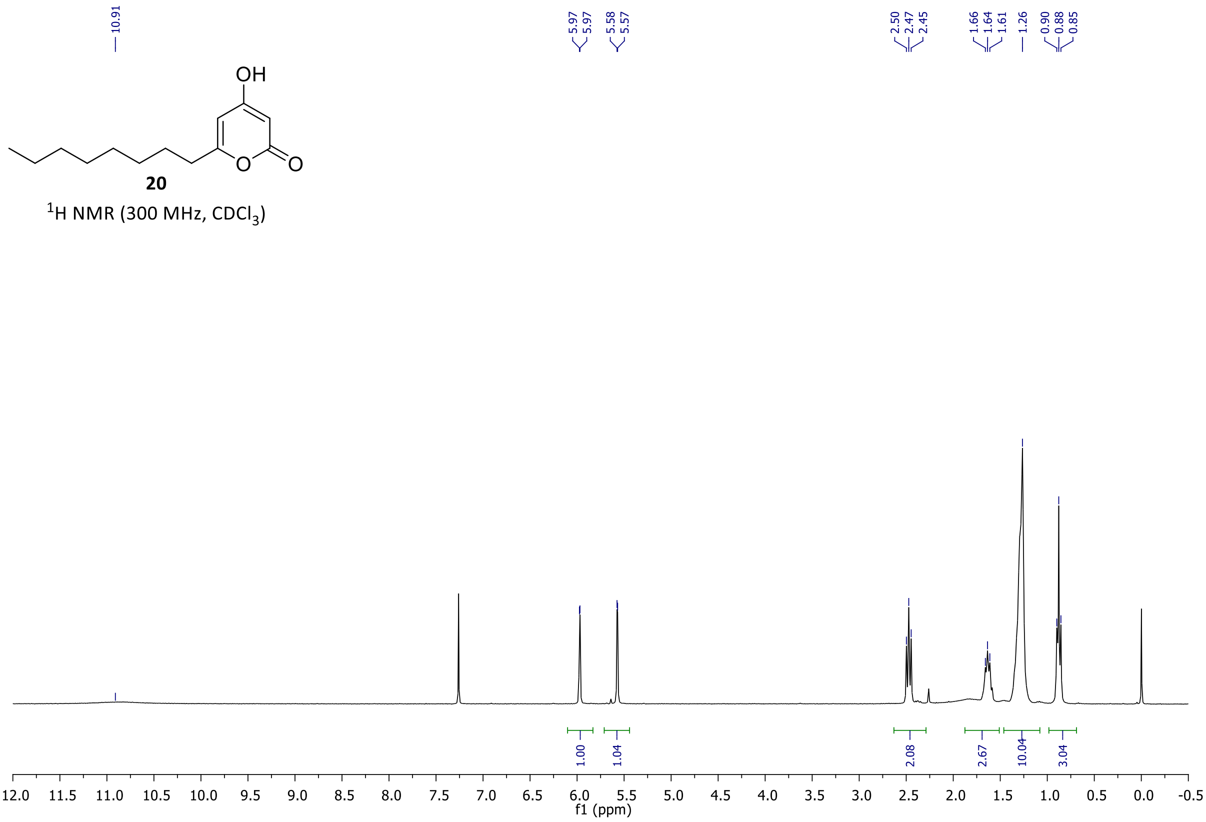

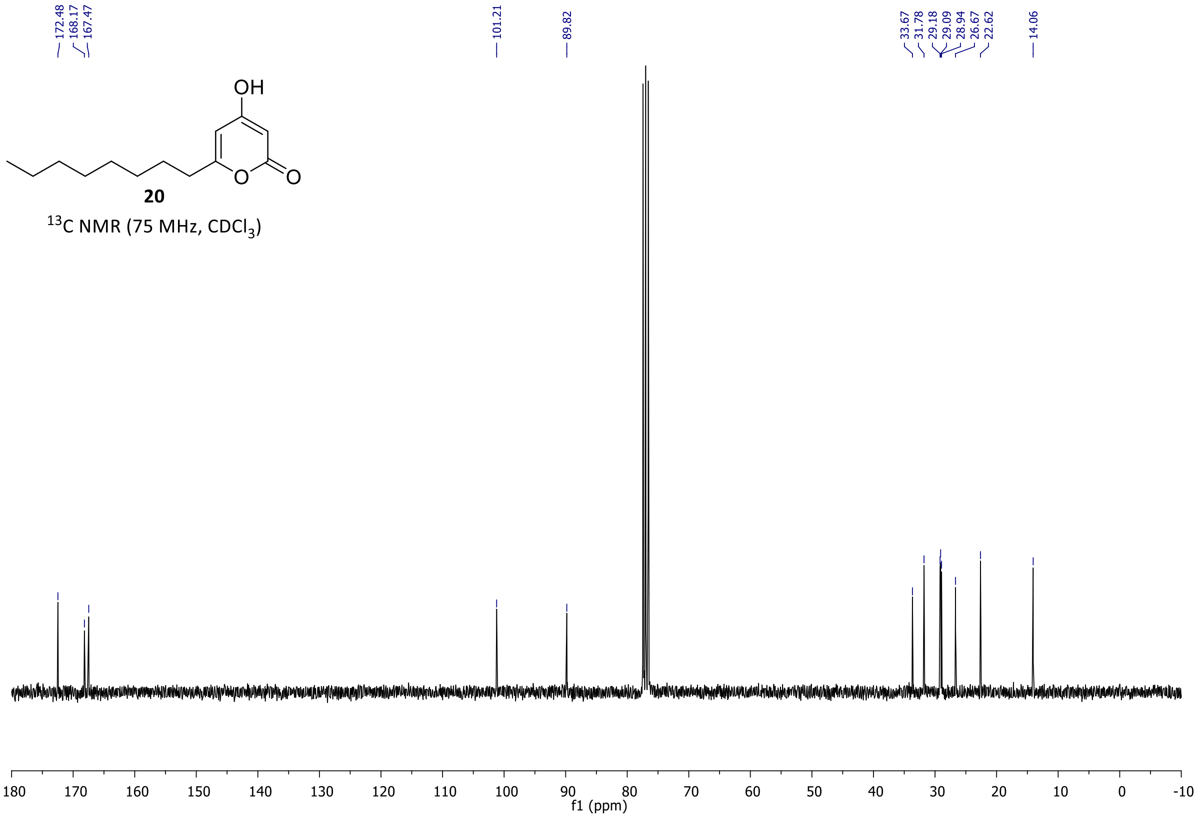

Figure S23: *^{1}H NMR (top) and ^{13}C NMR (bottom) spectra for compound **20**.*

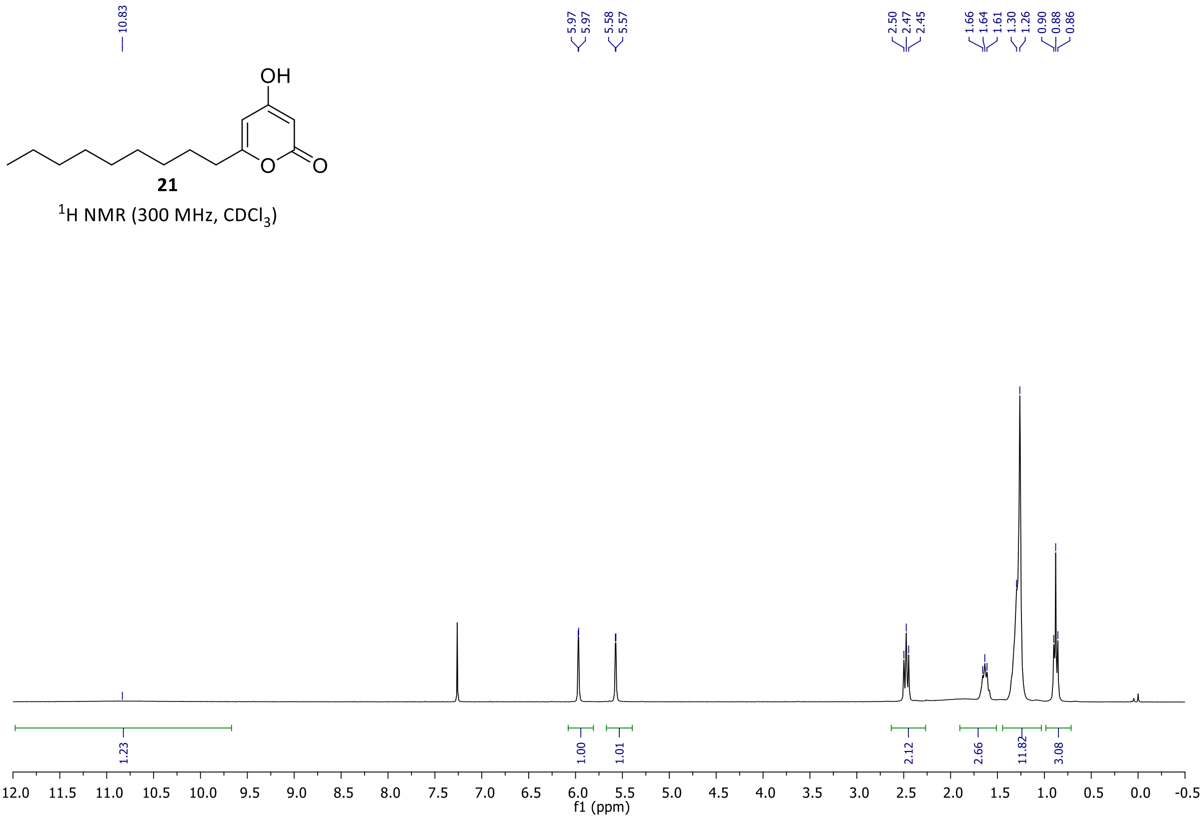

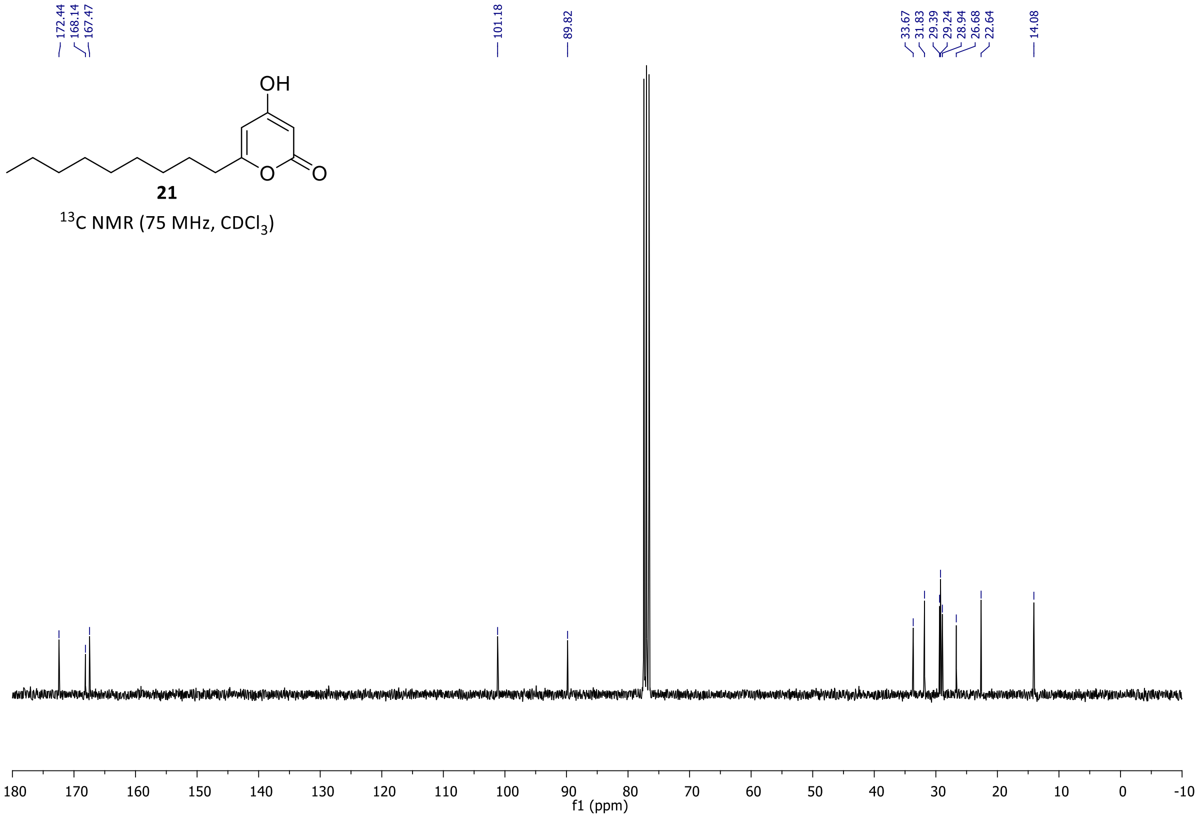

Figure S24: *^{1}H NMR (top) and ^{13}C NMR (bottom) spectra for compound **21**.*

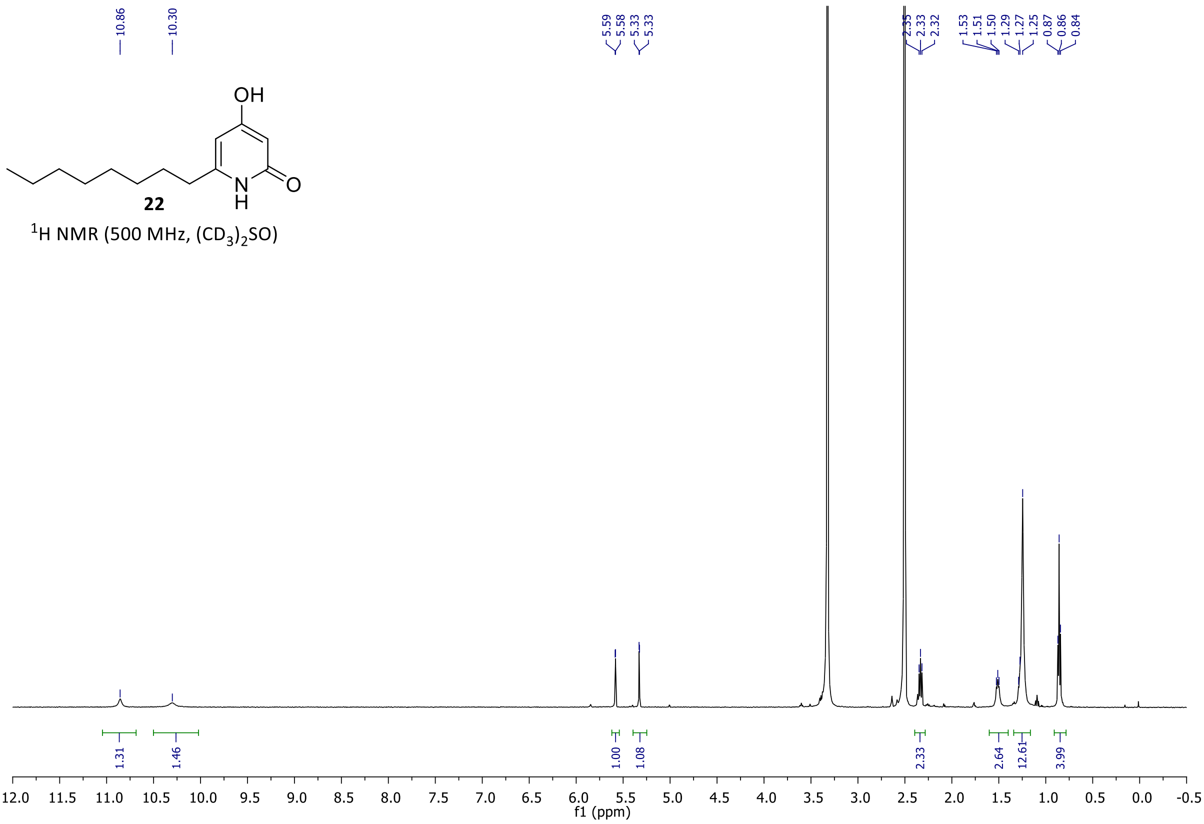

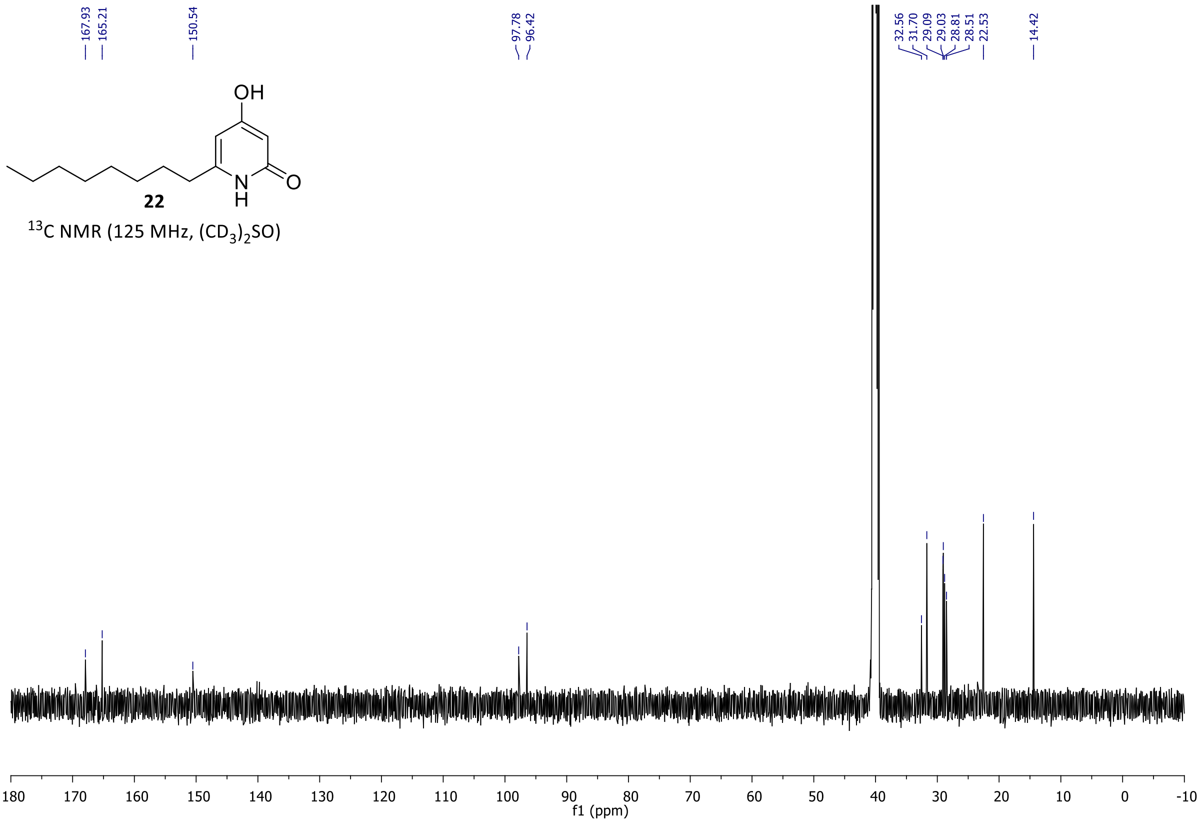

***Figure S25:** ^{1}H NMR (top) and ^{13}C NMR (bottom) spectra for compound **22**.*

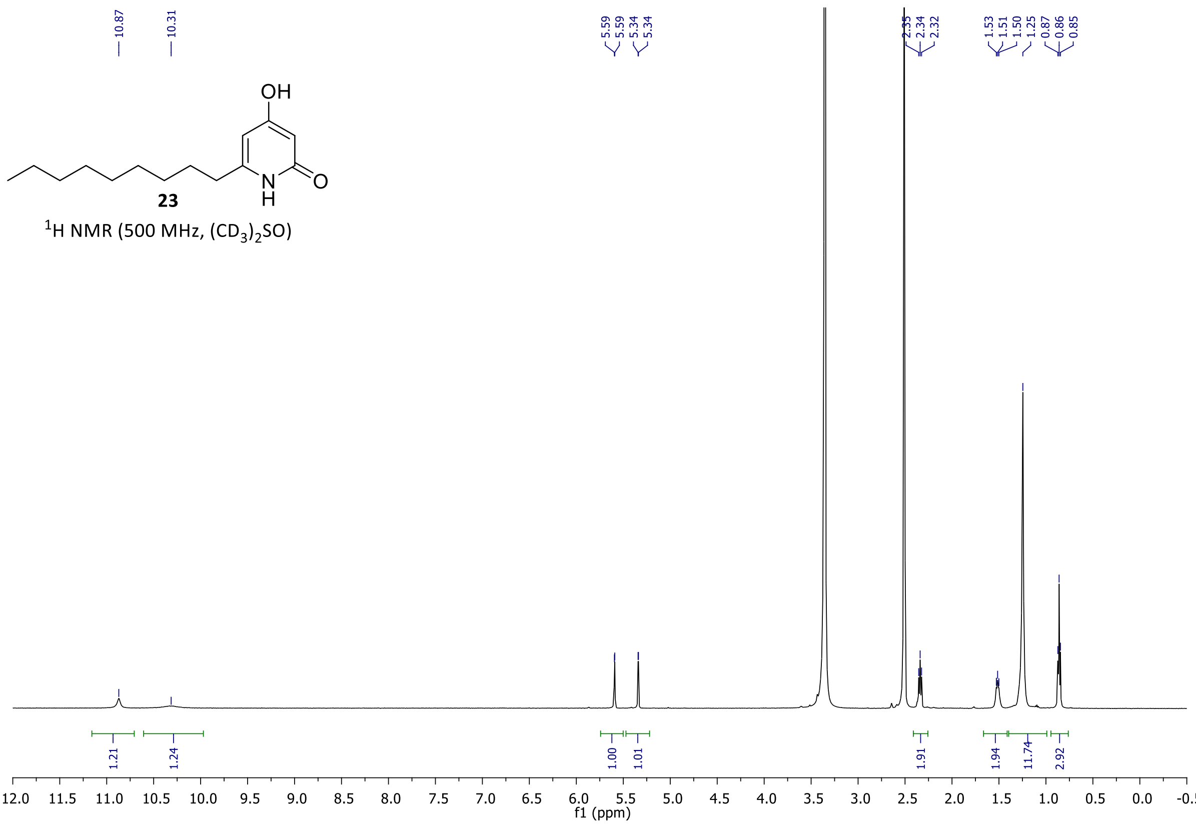

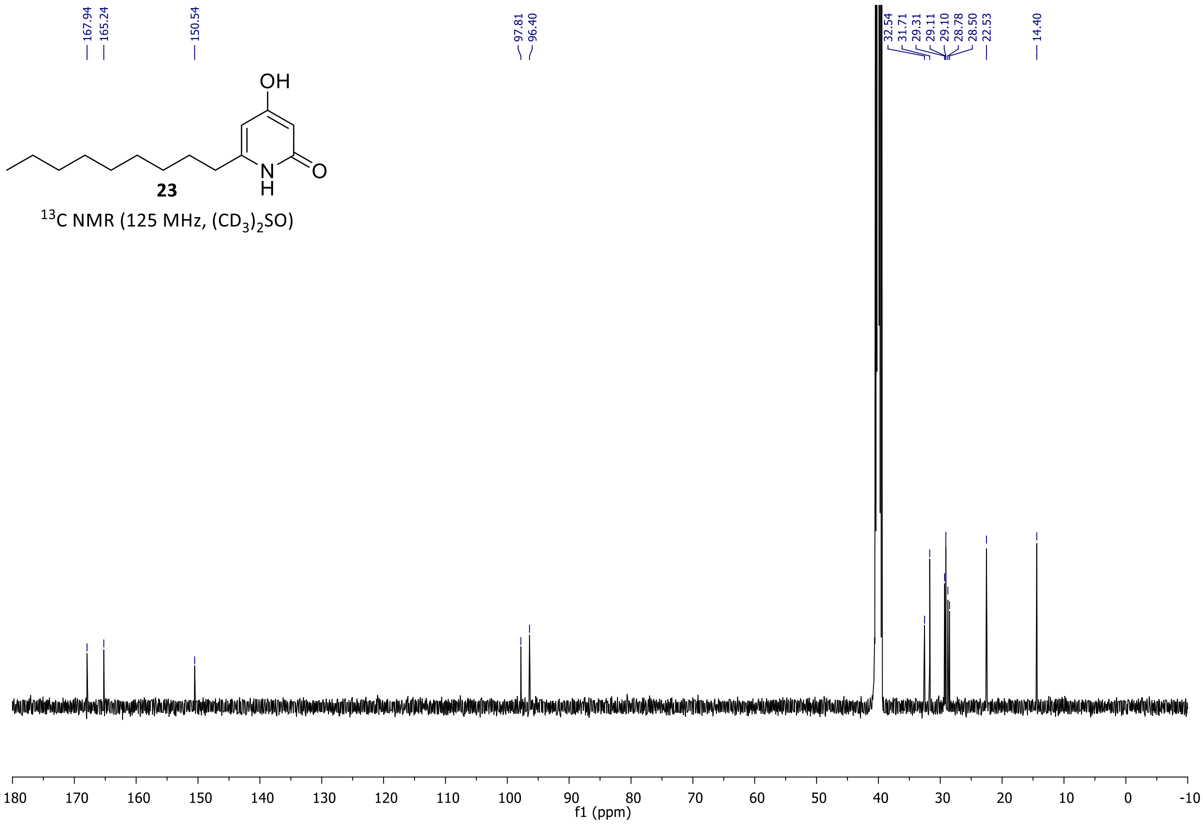

***Figure S26:** ¹H NMR (top) and ¹³C NMR (bottom) spectra for compound **23**.*

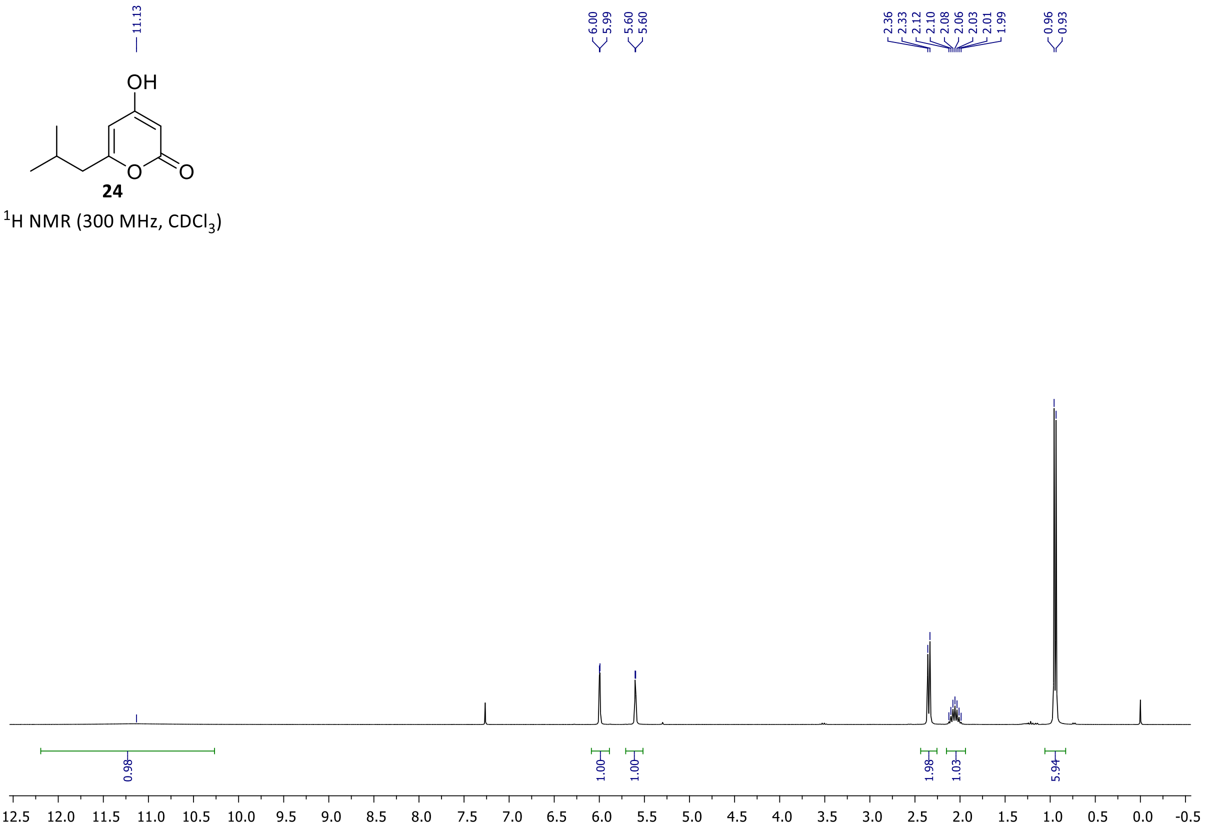

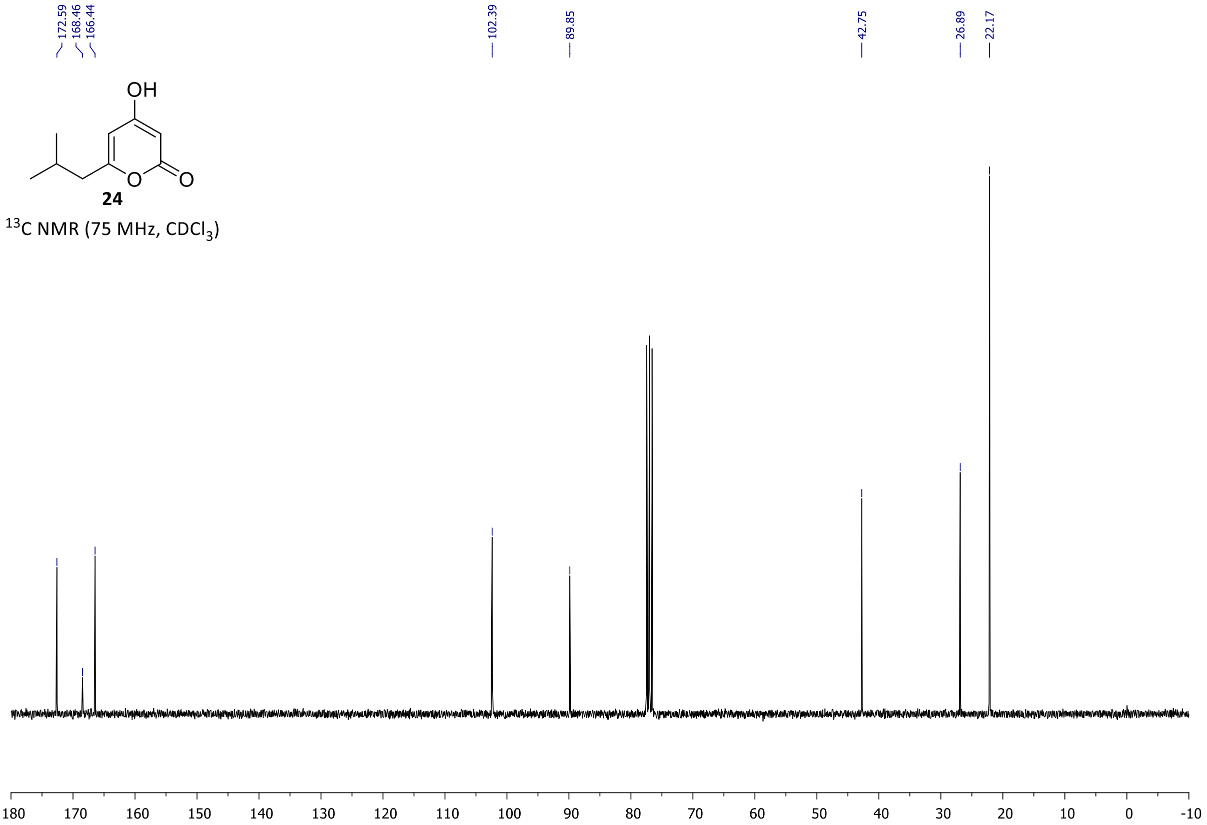

Figure 27: *^{1}H NMR (top) and ^{13}C NMR (bottom) spectra for compound **24**.*

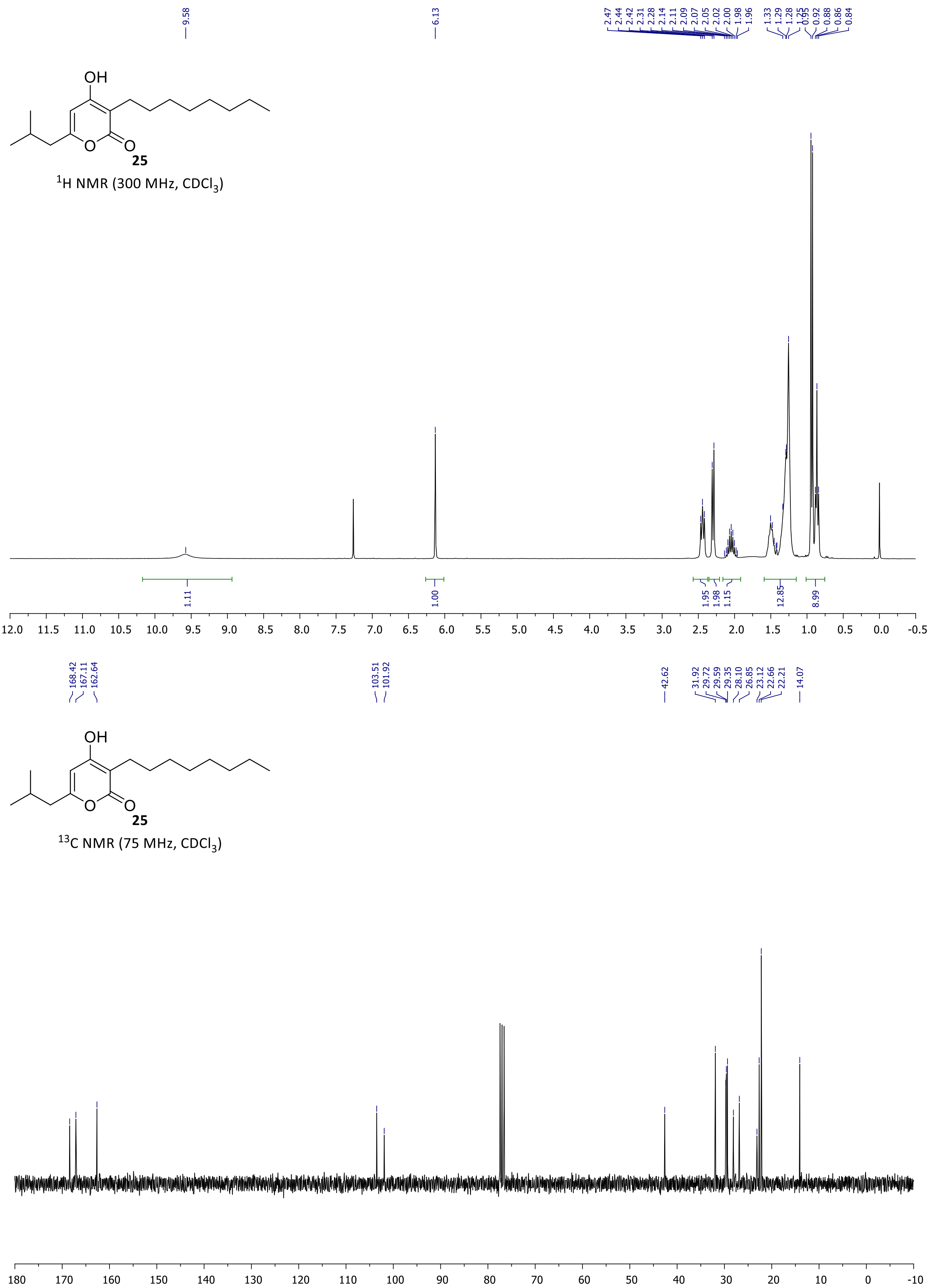

Figure S28: *^{1}H NMR (top) and ^{13}C NMR (bottom) spectra for compound **25**.*

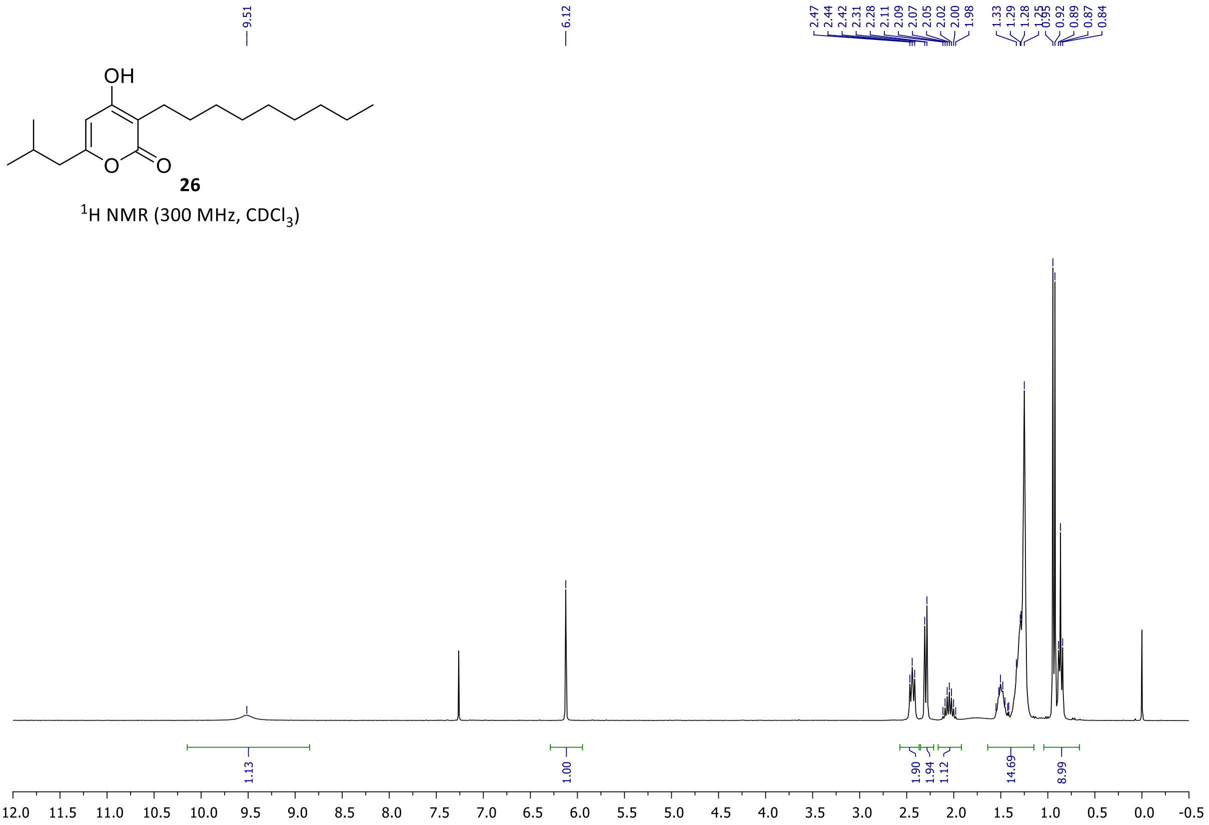

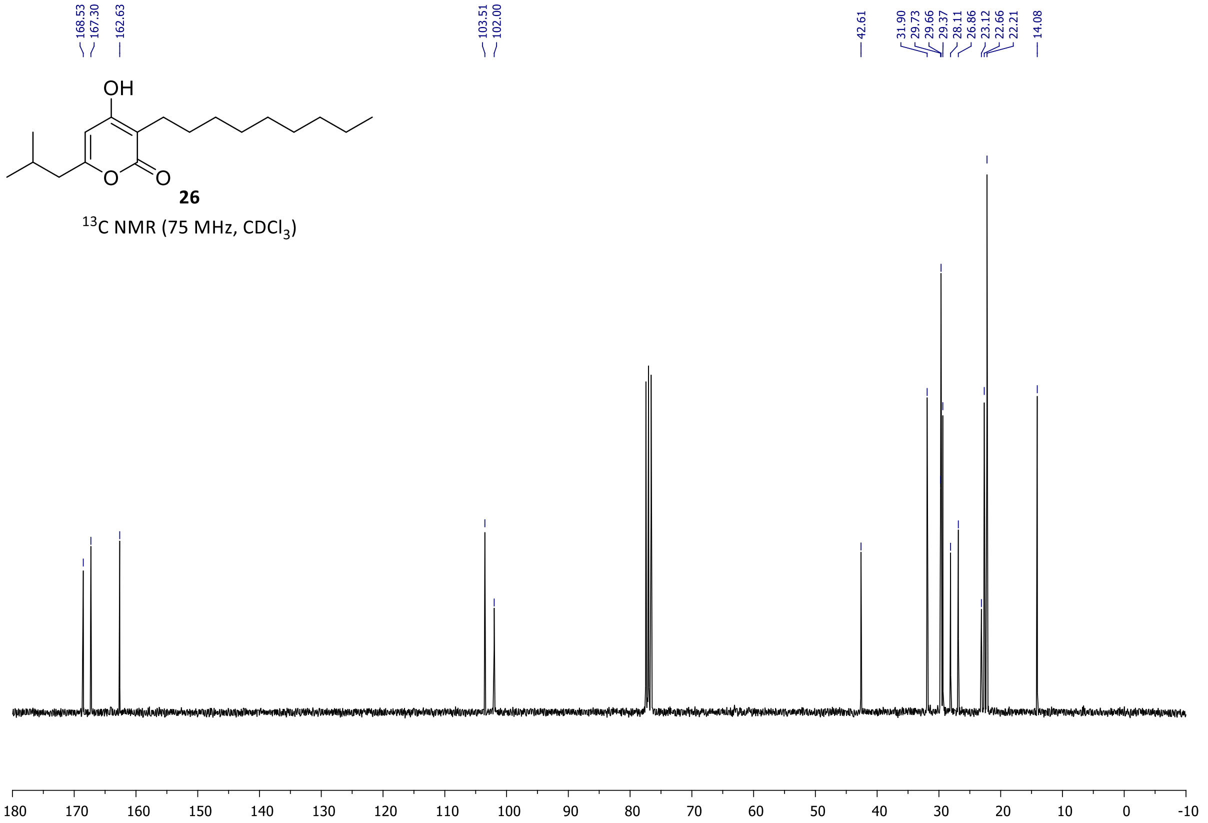

Figure S29: *^{1}H NMR (top) and ^{13}C NMR (bottom) spectra for compound **26**.*

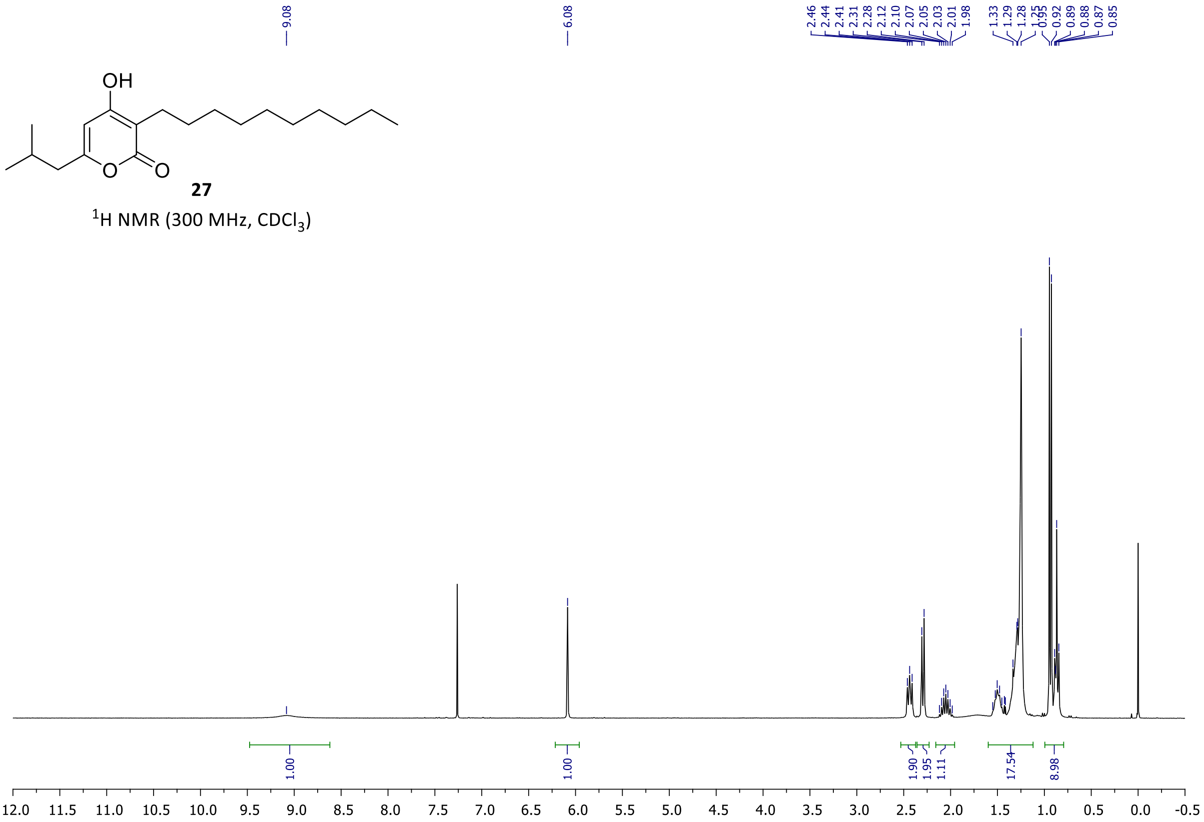

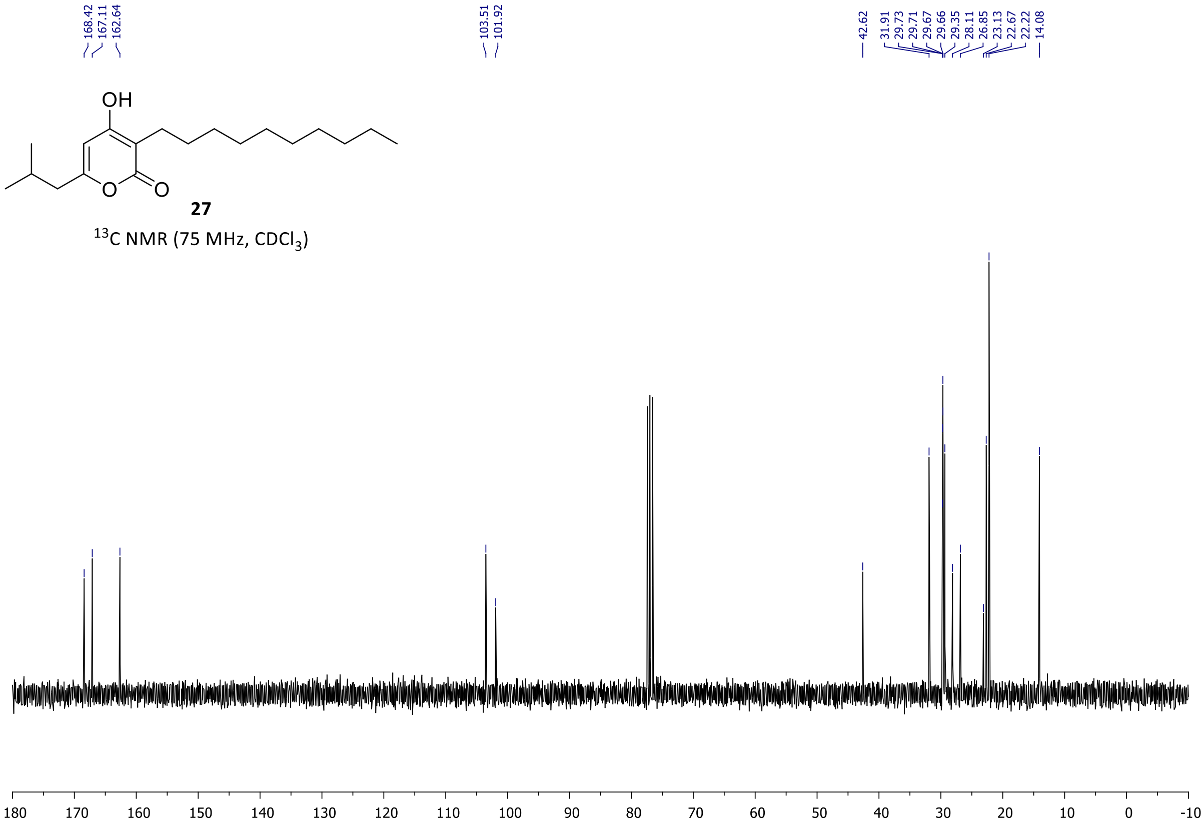

Figure S30: *^{1}H NMR (top) and ^{13}C NMR (bottom) spectra for compound **27**.*

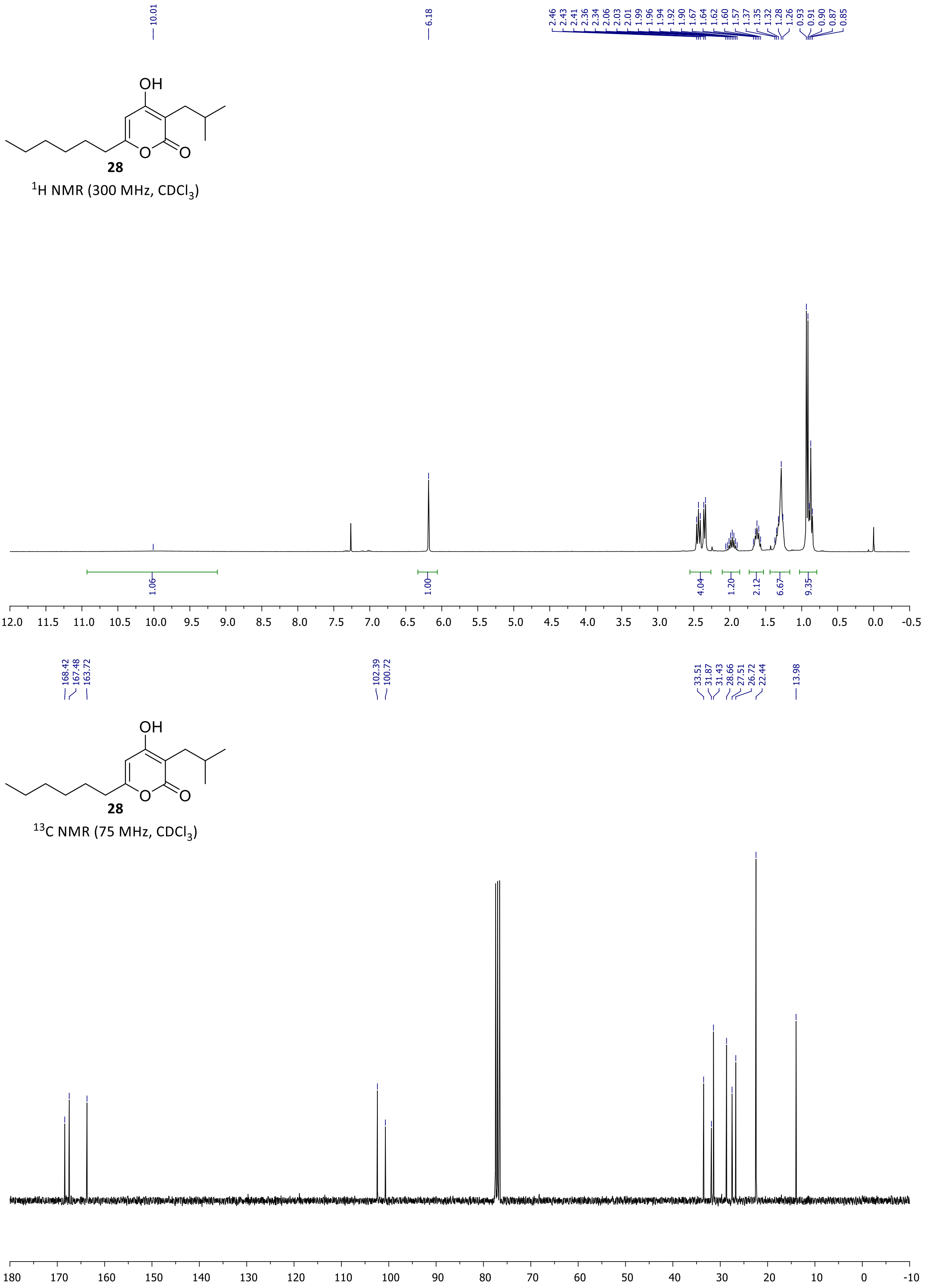

Figure S31: *^{1}H NMR (top) and ^{13}C NMR (bottom) spectra for compound **28**.*

References

1. Kraus, G. A.; Basemann, K.; Guney, T., Selective pyrone functionalization: reductive alkylation of triacetic acid lactone. *Tetrahedron Lett.* **2015**, *56*, 3494-3496.
2. Moreno-Mañas, M.; Pleixats, R., A Method for the Alkylation at C-3 of 4-Hydroxy-6-methyl-2-pyrone (Triacetic Acid Lactone). *Synthesis* **1984**, 430-431.
3. Tempone, A. G.; Ferreira, D. D.; Lima, M. L.; Costa Silva, T. A.; Borborema, S. E. T.; Reimão, J. Q.; Galuppo, M. K.; Guerra, J. M.; Russell, A. J.; Wynne, G. M.; Lai, R. Y. L.; Cadelis, M. M.; Copp, B. R., Efficacy of a series of alpha-pyrone derivatives against Leishmania (L.) infantum and Trypanosoma cruzi. *Eur. J. Med. Chem.* **2017**, *139*, 947-960.
4. Moreno-Manas, M.; Ribas, J.; Virgili, A., Palladium-catalyzed C-alkylations of the highly acidic and enolic triacetic acid lactone. Mechanism and stereochemistry. *J. Org. Chem.* **1988**, *53*, 5328-5335.
5. Swidorski, J. J.; Wang, J.; Hsung, R. P., A Concise Total Synthesis of (−)-Cylindricine C through a Stereoselective Intramolecular Aza-[3 + 3] Annulation Strategy. *Org. Lett.* **2006**, *8*, 777-780.
6. Lokot, I. P.; Pashkovsky, F. S.; Lakhvich, F. A., A new approach to the synthesis of 3,6- and 5,6-dialkyl derivatives of 4-hydroxy-2-pyrone. Synthesis of rac-germicidin. *Tetrahedron* **1999**, *55*, 4783-4792.
7. Poulton, G. A.; Cyr, T. D., Pyrones. IX. Synthetic approaches to the fungal metabolite phacidin and its derivatives. *Can. J. Chem.* **1982**, *60*, 2821-2829.
8. Katritzky, A. R.; Wang, Z.; Wang, M.; Hall, C. D.; Suzuki, K., Facile Syntheses of 2,2-Dimethyl-6-(2-oxoalkyl)-1,3-dioxin-4-ones and the Corresponding 6-Substituted 4-Hydroxy-2-pyrones. *J. Org. Chem.* **2005**, *70*, 4854-4856.
9. Giddens, A. C.; Nielsen, L.; Boshoff, H. I.; Tasdemir, D.; Perozzo, R.; Kaiser, M.; Wang, F.; Sacchettini, J. C.; Copp, B. R., Natural product inhibitors of fatty acid biosynthesis: synthesis of the marine microbial metabolites pseudopyronines A and B and evaluation of their anti-infective activities. *Tetrahedron* **2008**, *64*, 1242-1249.
10. Pardo, L. M.; Prendergast, A. M.; Nolan, M.-T.; Ó Muimhneacháin, E.; McGlacken, G. P., Pd/Pivalic Acid Mediated Direct Arylation of 2-Pyrones and Related Heterocycles. *Eur. J. Org. Chem.* **2015**, 3540-3550.
11. Demuner, A. J.; Valente, V. M. M.; Barbosa, L. C. A.; Rathi, A. H.; Donohoe, T. J.; Thompson, A. L., Synthesis and phytotoxic activity of new pyridones derived from 4-hydroxy-6-methylpyridin-2(1H)-one. *Molecules* **2009**, *14*, 4973-4986.
12. Liu, Y.; Zhang, Q.; Chen, L.-H.; Yang, H.; Lu, W.; Xie, X.; Nan, F.-J., Design and Synthesis of 2-Alkylpyrimidine-4,6-diol and 6-Alkylpyridine-2,4-diol as Potent GPR84 Agonists. *ACS Med. Chem. Lett.* **2016**, *7*, 579-583.
